# *N*-Heterocyclic Carbene-Iridium Complexes as Photosensitizers for In Vitro Photodynamic Therapy to Trigger Non-Apoptotic Cell Death in Cancer Cells

**DOI:** 10.3390/molecules28020691

**Published:** 2023-01-10

**Authors:** Xing Wang, Chen Zhang, Ryma Madji, Camille Voros, Serge Mazères, Christian Bijani, Céline Deraeve, Olivier Cuvillier, Heinz Gornitzka, Marie-Lise Maddelein, Catherine Hemmert

**Affiliations:** 1 Coordination Chemistry Laboratory of the National Centre for Scientific Research (LCC-CNRS), University of Toulouse, CNRS, Université Toulouse III–Paul Sabatier (UPS), 31077 Toulouse, France; 2 Institut de Pharmacologie et de Biologie Structurale (IPBS), Université de Toulouse, CNRS, Université Toulouse III–Paul Sabatier (UPS), 31077 Toulouse, France

**Keywords:** iridium, *N*-heterocyclic carbene, luminescence, photodynamic therapy, cell death, apoptosis, caspase, PARP, Bcl-xL

## Abstract

A series of seven novel iridium complexes were synthetized and characterized as potential photosensitizers for photodynamic therapy (PDT) applications. Among them, four complexes were evaluated in vitro for their anti-proliferative activity with and without irradiation on a panel of five cancer cell lines, namely PC-3 (prostate cancer), T24 (bladder cancer), MCF7 (breast cancer), A549 (lung cancer) and HeLa (cervix cancer), and two non-cancerous cell models (NIH-3T3 fibroblasts and MC3T3 osteoblasts). After irradiation at 458 nm, all tested complexes showed a strong selectivity against cancer cells, with a selectivity index (SI) ranging from 8 to 34 compared with non-cancerous cells. The cytotoxic effect of all these complexes was found to be independent of the anti-apoptotic protein Bcl-xL. The compound exhibiting the best selectivity, complex **4a**, was selected for further investigations. Complex **4a** was mainly localized in the mitochondria. We found that the loss of cell viability and the decrease in ATP and GSH content induced by complex **4a** were independent of both Bcl-xL and caspase activation, leading to a non-apoptotic cell death. By counteracting the intrinsic or acquired resistance to apoptosis associated with cancer, complex **4a** could be an interesting therapeutic alternative to be studied in preclinical models.

## 1. Introduction

According to the World Health Organization (WHO), cancer is a major public health problem worldwide in all populations regardless of wealth or social status and is the second most common cause of death, with nearly 10 million deaths worldwide in 2020 [1,2]. Currently, the metallo anti-cancer drug cisplatin and its derivatives (carboplatin, oxaliplatin, nedaplatin, lobaplatin and heptaplatin) are widely used in the clinic and account for more than 50% of chemotherapy treatments [3]. However, the use of cisplatin and its derivatives is limited by high toxicity to normal cells, resulting in significant side effects such as nausea, vomiting, neurotoxicity, hepatotoxicity, renal toxicity, allergic reactions and intrinsic or acquired resistance [4,5]. In an effort to overcome these limitations, the development of transition metal complexes for cancer therapy is the subject of extensive and ongoing research to obtain effective alternative drugs with a superior toxicity profile [6,7,8,9,10]. The therapeutic potential of transition metal-based compounds in cancer therapy has attracted much interest mainly due to their unique properties. Transition metals exhibit different oxidation states and can interact with a number of negatively charged molecules. Their charge can be modified to generate species in aqueous solutions that can bind to charged biological molecules. The ancillary ligands can be easily modulated to adjust steric and/or electronic properties or can be combined with biologically active molecules to enhance therapeutic and/or synergistic effects. In addition, transition metal complexes often exhibit electrochemical properties and thus participate in various biological redox reactions or photophysical properties that may make them good candidates for photodynamic therapy (PDT). In PDT, non-toxic photosensitizers can be activated by light irradiation, leading to cell death. The only commercially available PDT pro-drugs are all based on porphyrin systems (Photofrin, Foscan, Tookad); only one non-porphyrinic compound, a ruthenium complex TLD-1433, is under clinical trials [11,12,13]. Next to ruthenium, iridium has gained a lot of attention in the last years in the field of PDT research.

Iridium complexes have been widely used in scientific imaging and sensing probes because they exhibit good photostability and cell permeability [14,15]. In the last seven years, they have also become a research hot spot in metallic anti-cancer drugs as theranostic or PDT agents. This is well illustrated by the growing number of articles related to this field and several recent reviews have summarized the main advances [16,17,18,19,20,21,22,23]. Iridium(III) complexes are readily accessible, highly soluble in water and stable in air and moisture. They exhibit tunable reactivity because they are substitutionally inert and possess low reduction potential. Among them, cyclometalated Ir(III) complexes display outstanding photophysical properties, such as excellent phosphorescence, long emission times, relatively high quantum yields, tunable emission colors, large Stokes shifts, two-photon absorption and photostability due to its high metal-centered state and easy modulation of HOMO and LUMO energy levels [24]. In addition, the photophysical and biological properties of iridium(Ⅲ) complexes can be easily tuned by the ligands.

Most anti-cancer iridium complexes are not designed to target a specific biomolecule in cells, and structure–activity relationships remain unclear. However, phosphorescent iridium complexes, exhibiting intrinsic cationic lipophilicity, mainly target mitochondria and trigger apoptosis [25,26,27,28,29,30,31,32,33,34,35,36,37,38,39,40,41,42], involving mitochondrial outer membrane permeabilization (MOMP), release of cytochrome *c* and activation of executioner caspases by mitochondrial ROS. Through structural modifications, iridium complexes can also target other subcellular organelles, such as lysosomes [43,44,45,46,47,48,49,50,51], the endoplasmic reticulum (ER) [52,53,54,55] or the nucleus [56,57]. 

However, regarding the iridium complexes targeting the mitochondria, it has been reported that a number of them can induce non-apoptotic forms of cell death, as recently reviewed [22,23]. Several studies conducted in various cancer cell models have suggested a paraptosis-like cell death [58,59,60] based on the absence of apoptosis markers (e.g., executioner caspases activation, release of cyt *c*, PARP processing) or the lack of response to caspase inhibitors associated with major morphological traits, notably vacuolization of the endoplasmic reticulum (ER) and/or mitochondria, activation of certain MAP kinases or transfer of calcium from the ER to the mitochondria. These are not unique features that may distinguish from other types of cell death, yet taken together they can describe paraptosis [61], even if little is known about the molecular basis of paraptosis 20 years after its original description. 

Others have also described non-apoptotic cell death associated with mitochondria swelling, plasma membrane blebbing and cytosol vacuolization and have suggested the induction of oncosis [62], a term sometimes used to describe necrosis induced by ischemia accompanied by cell and organelle swelling and depletion of ATP, but not biochemically characterized so far.

Ferroptosis, a type of cell death characterized by the accumulation of lipid peroxides and the downregulation of glutathione peroxidase 4 (GPX4), has been proposed as the main mechanism of action of cyclometalated benzothiophenylisoquinoline-derived iridium complexes, notably utilized under anoxic conditions [63]. 

Based on the combination of anti-cancer properties of metal-*N* heterocyclic carbenes (NHCs) and cyclometalated iridium(III) complexes, the group of Mao developed series of cyclometalated Ir(Ⅲ) complexes containing bis-NHC ligands bearing methyl, ethyl or *n*-butyl groups and differing in their C^N ligands as mitochondria-targeted theranostic and photodynamic anti-cancer agents [27,35,64]. The cytotoxicity of the complexes was found to be correlated with their ancillary ligands, lipophilicities and cellular uptake capability. These complexes display high phototoxicity activities. With phenylpyridine as C^N ligands, they display up to three orders of magnitude higher cytotoxicity against cisplatin resistant A549 cells upon irradiation at 365 nm. The authors further extended the π conjugation of the C^N ligands to achieve longer wavelength emitting complexes in order to obtain a higher penetration depth and tested their photocytotoxicity in 450 and 630 nm LED lights. All these complexes induce a series of events associated with mitochondrial damage, including ROS production, permeabilization of MMP and apoptosis. 

Herein, we report the synthesis and the full characterization of six cationic and one neutral new iridium(III) complexes containing two phenylpyridine (ppy) ligands and mono or bis-NHC ligands. These complexes have been studied for their cytotoxic activities (one series for its PDT properties) and mechanistical studies have been conducted on the most potent complex.

## 2. Results and Discussion

### 2.1. Synthesis and Characterization of Cyclometalated Iridium (NHC) Complexes

**Figure 1:**
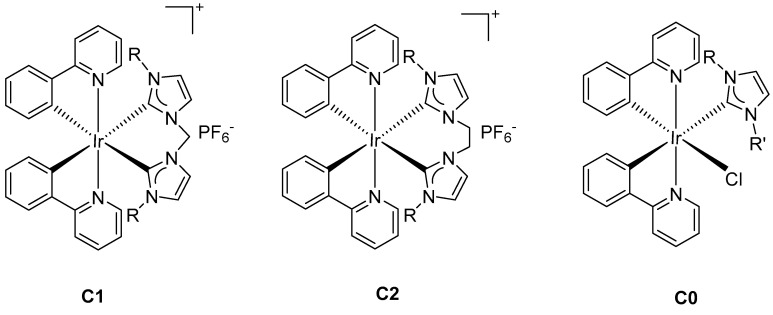
Three designed families of iridium(III) NHC complexes.

The three designed families of cyclometalated iridium(III) NHC complexes involving ppy C^N ligands and bearing different NHC ligands are represented in Figure 1. The first two families contain a bis-carbene ligand, in which the two NHCs are bridged by a methylene (C1) and an ethylene (C2), giving rise to cationic complexes, while the other one (C0) contains only one mono-carbene ligand and one chloride, leading to a neural complex. The NHC ligands contain aliphatic or aromatic groups.

**Scheme 1:**
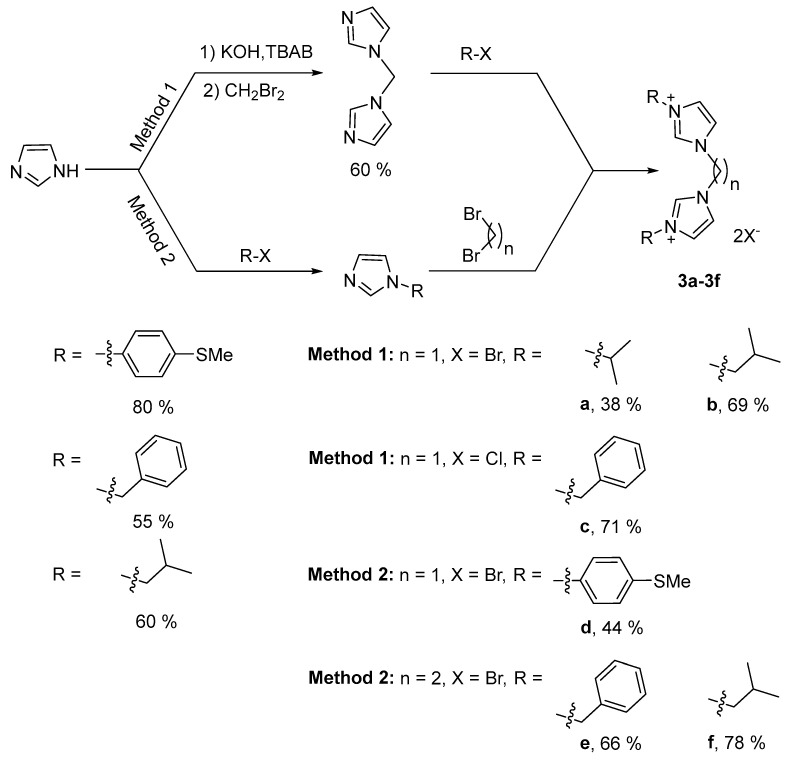
Synthesis of proligands **3a**–**3f**.

The bis(imidazolium) salts **3a** to **3f** were synthesized by two different methods (Scheme 1). Method 1: Firstly, 1,1′-methylene bis(1*H*-imidazole) was prepared using a modified literature procedure, with a yield of 60% [65,66]. It was then reacted with 2.2 equivalents of an alkyl halide, 2-bromopropane (**a**) or 1-bromo-2-methylpropane (**b**) under reflux for 48 h and benzyl chloride (**c**) in CH_3_CN under reflux for 10 h, respectively, to afford the carbene precursors **3a–3c** with yields ranging from 38% to 71%. Method 2: Mono-substituted imidazoles were readily obtained by *N*-alkylation of imidazole with 4-bromo-thioanisole (**d**) in the presence of K_2_CO_3_ and a catalytic amount of CuSO_4_ at 205 °C for 10 h, benzyl chloride (**e**) in the presence of K_2_CO_3_ in DMF at 50 °C for 2 h and isobutyl bromide (**f**) in the presence of KOH in DMSO at room temperature for 2 h, with yields ranging from 55% to 80%. These substituted imidazoles were then reacted with an excess of dibromomethane under reflux for 10 h (**3d**), dibromoethane in toluene under reflux for 24 h (**3e**) or dibromoethane at 110 °C for 72 h (**3f**), to give the desired proligands **3d**–**3f** with yields ranging from 44% to 78% for this second step.

The proligands **3a** to **3f** were characterized by ^1^H, ^13^C NMR spectroscopy, elementary analysis and mass spectrometry. The most notable feature in the ^1^H and ^13^C NMR spectra of the bis(imidazolium) salts are the resonances for imidazolium protons (H2) located between 9.06–10.31 ppm and the corresponding imidazolium carbons (C2) in the range of 137.0–138.4 ppm. The mass spectra of the proligands exhibit the classical peaks corresponding to [M − X^−^]^+^ and/or [M − 2X^−^ − H^+^]^+^ cations.

**Scheme 2:**
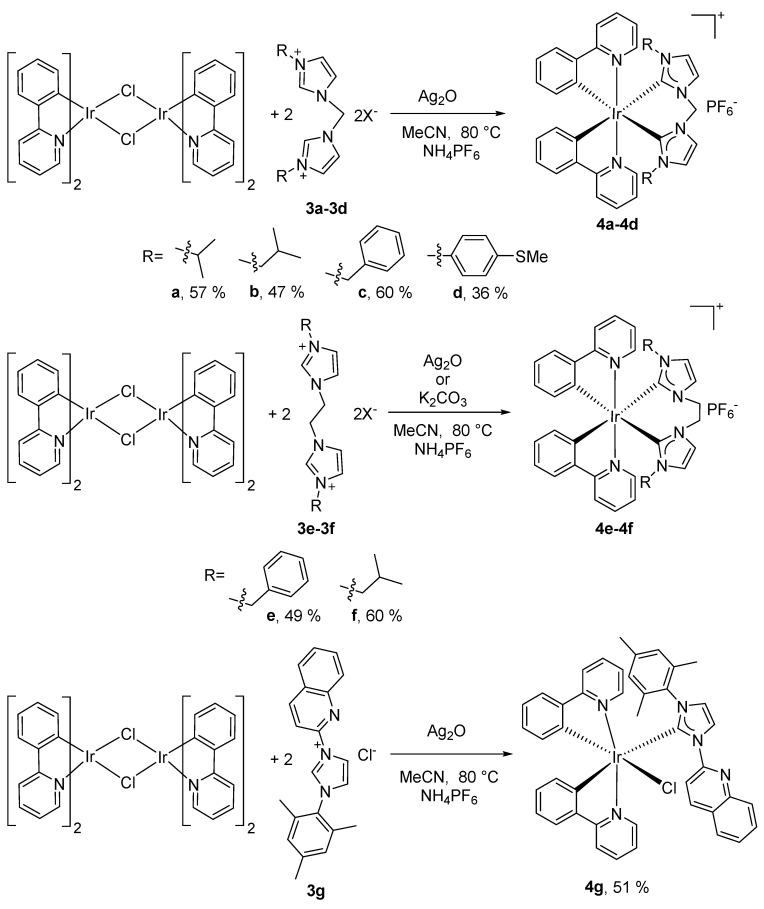
Synthesis of cyclometalated iridium(III) NHC complexes **4a**–**4g**.

The iridium precursor [IrCl(ppy)_2_]_2_ and the carbene precursor **3g** were prepared according to the literature procedures [67,68]. The 4-methylthio-benzyl and quinolinyl substituents have been chosen due to interesting biological results obtained with NHC-gold complexes containing such substituents. The iridium(III) NHC complexes **4a**–**4e** and **4g** (Scheme 2) were synthetized by reacting [IrCl(ppy)_2_]_2_ with two equivalents of carbene precursors **3a** to **3e** or **3g** in the presence of the mild base Ag_2_O in dry acetonitrile at 80 °C for 10 h. In the case of complex **4f**, the use of Ag_2_O led to some by-products, so we turned to use K_2_CO_3_ to form the carbene. An anion metathesis was performed by adding an excess of NH_4_PF_6_. The iridium complexes bis-NHC **4a**–**4f** were purified by column chromatography and obtained as yellow powders with yields ranging from 36 to 60%. All the synthesized iridium complexes are soluble in CH_3_CN, MeOH and DMSO. NMR spectroscopy unequivocally manifested the formation of the iridium(III) complexes with the absence of the proton resonance of the acidic imidazolium and the ^13^C NMR spectra showed the resonance for the carbenic carbons in the range of 162.7–164.9 ppm for complexes **4a** to **4f** in good agreement with reported values for iridium(III) bis-NHC complexes [64] and 180.4 ppm for complex **4g**, in line with the reported values for iridium mono-NHC complexes [69]. It should be noted that, since the structure of complex **4g** is not symmetrical, all the signals of the protons and carbon of the ppy ligands are split. The elemental analyses of the iridium complexes correspond to the general formula [Ir(ppy)_2_L][PF_6_] for **4a**–**4f** and Ir(ppy)_2_(L)Cl for **4g**. The high-resolution mass spectra displayed the classical peaks corresponding to the cationic fragment [M − PF_6_^−^]^+^ for **4a**–**4f** and the cationic fragment [M − Cl^−^]^+^ for **4g**. For the configuration of iridium complexes, we obtained two different isomers, Λ (lambda) and Δ (delta), which cannot be distinguished by NMR spectroscopy.

Lipophilicities of all complexes have been measured by the classical shake-flask method, using UV absorption [70]. The *Log p* values range from -0.063 to 0.861 in increasing order –0.063 (**4a**), 0.035 (**4d**), 0.519 (**4f**), 0.581 (**4c**), 0.842 (**4b**), 0.847 (**4e**) and 0.861 (**4g**). 

### 2.2. Crystal Structures of Complexes **4a**–**4c** and **4e**–**4f**

Crystals of **4a** to **4c** and **4e** to **4f** have been obtained by gas phase diffusion of diethyl ether in a dichloromethane solution of these complexes.

**Figure 2:**
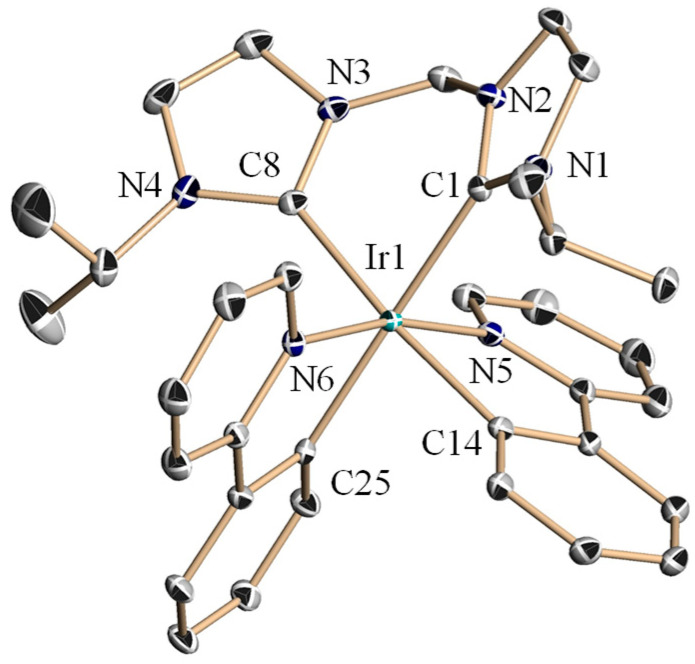
Molecule structure of **4a** in the solid state. Hydrogen atoms and non-coordinating anions are omitted for clarity. Selected distances [Å] and angles [°]: Ir-C1 2.120(4), Ir-C8 2.102(4), Ir-C14 2.057(4), Ir-C25 2.049(4), Ir-N5 2.069(3), Ir-N6 2.056(3), C25-Ir1-N6 79.85(11), C25-Ir1-C14 86.27(12), N6-Ir1-C14 91.88(12), C25-Ir1-N5 90.95(12), N6-Ir1-N5 167.82(10), C14-Ir1-N5 79.47(12), C25-Ir1-C8 97.75(12), N6-Ir1-C8 94.16(12), C14-Ir1-C8 173.25(13), N5-Ir1-C8 94.99(12), C25-Ir1-C1 177.74(13), N6-Ir1-C1 98.53(11), C14-Ir1-C1 92.22(12), N5-Ir1-C1 90.43(11), C8-Ir1-C1 83.91(12).

**Figure 3:**
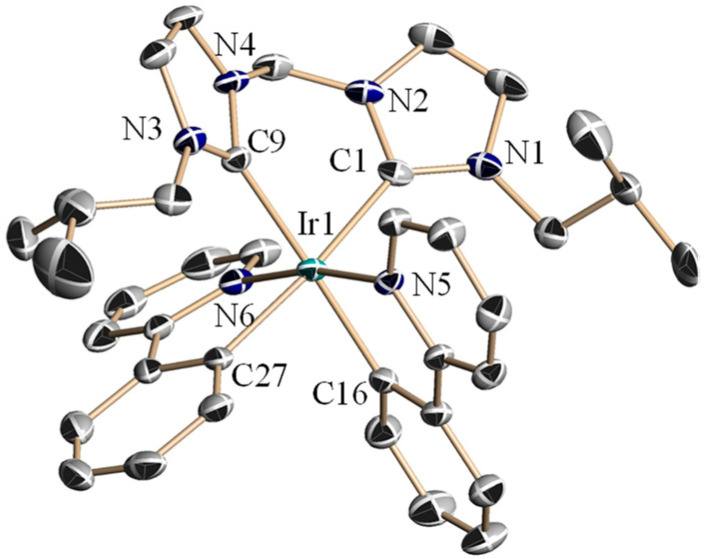
Molecule structure of **4b** in the solid state. Hydrogen atoms, disorder of the side arms and non-coordinating anions are omitted for clarity. Selected distances [Å] and angles [°]: Ir1-C1 2.107(3), Ir1-C9 2.130(3), Ir1-C16 2.049(3), Ir1-C27 2.052(3), Ir1-N5 2.051(2), Ir1-N6 2.058(2), C16-Ir1-N5 80.00(11), C16-Ir1-C27 84.74(11), N5-Ir1-C27 94.39(12), C16-Ir1-N6 91.27(11), N5-Ir1-N6 169.93(10), C27-Ir1-N6 79.69(12), C16-Ir1-C1 98.77(12), N5-Ir1-C1 92.36(11), C27-Ir1-C1 172.84(12), N6-Ir1-C1 93.95(11), C16-Ir1-C9 175.17(12), N5-Ir1-C9 96.41(10), C27-Ir1-C9 92.34(12), N6-Ir1-C9 91.99(10), C1-Ir1-C9 84.56(12).

**Figure 4:**
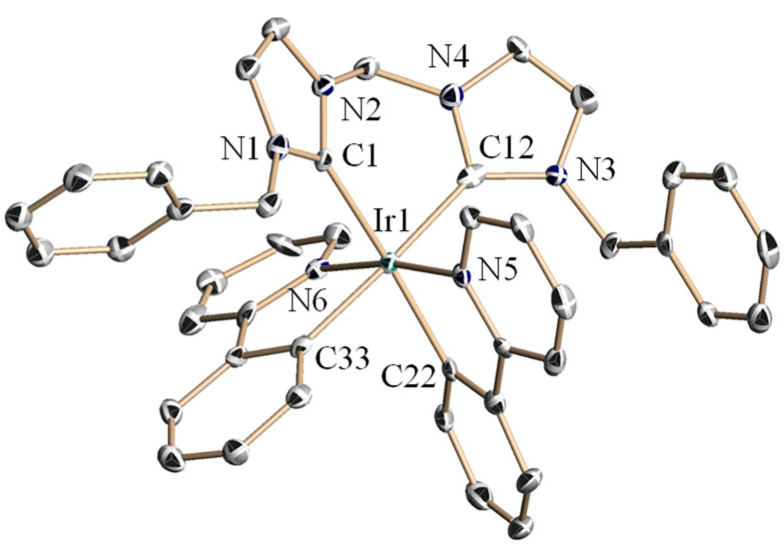
Molecule structure of **4c** in the solid state. Hydrogen atoms, non-coordinating anions and disordered dichloromethane are omitted for clarity. Selected distances [Å] and angles [°]: Ir1-C1 2.113(8), Ir1-C12 2.086(10), Ir1-C22 2.055(8), Ir1-C33 2.066(9), Ir1-N5 2.055(8), Ir1-N6 2.056(7), C22-Ir1-N5 80.2(3), C22-Ir1-N6 92.4(3), N5-Ir1-N6 171.5(3), C22-Ir1-C33 81.1(3), N5-Ir1-C33 95.4(4), N6-Ir1-C33 79.3(3), C22-Ir1-C12 100.4(3), N5-Ir1-C12 89.1(3), N6-Ir1-C12 96.4(3), C33-Ir1-C12 175.5(4), C22-Ir1-C1 172.8(3), N5-Ir1-C1 95.0(3), N6-Ir1-C1 91.9(3), C33-Ir1-C1 94.0(3), C12-Ir1-C1 84.9(3).

**Figure 5:**
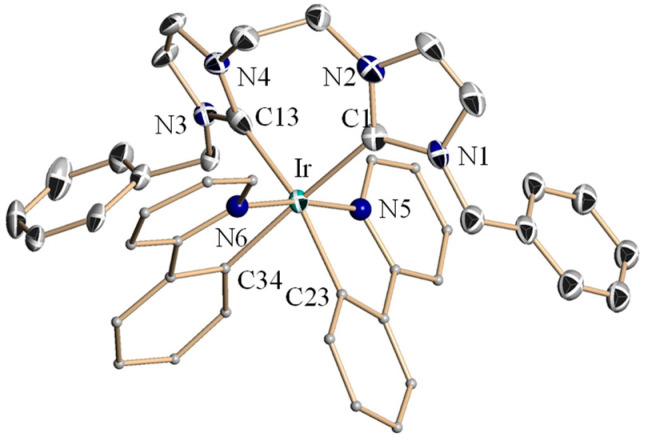
Molecule structure of **4e** in the solid state. Only one of two cations presented in the asymmetric unit is shown. Hydrogen atoms, anions, disordered ether molecules and a disorder of one benzyl substituent are omitted and ppy ligands are simplified for clarity. Selected distances [Å] and angles [°]: Ir-C1 2.087(10), Ir-C13 2.161(9), Ir-C23 2.049(8), Ir-C34 2.043(9), Ir-N5 2.041(7), Ir-N62.027(7), N6-Ir1-N5 172.7(3), N6-Ir1-C34 80.4(3), N5-Ir1-C34 92.7(3), N6-Ir1-C23 96.9(3), N5-Ir1-C23 79.1(3), C34-Ir1-C23 77.3(3), N6-Ir1-C1 95.9(3), N5-Ir1-C1 90.1(3), C34-Ir1-C1 165.0(3), C23-Ir1-C1 88.8(3), N6-Ir1-C13 88.7(3), N5-Ir1-C13 94.9(3), C34-Ir1-C13 99.0(3), C23-Ir1-C13 172.7(3), C1-Ir1-C13 95.4(3).

**Figure 6:**
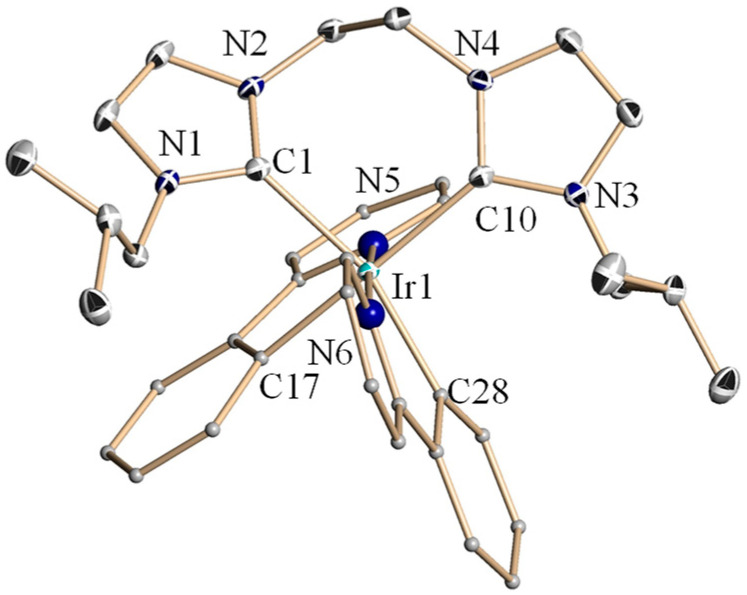
Molecule structure of **4f** in the solid state. Hydrogen atoms, anions and a disorder of one side arm are omitted and ppy ligands are simplified for clarity. Selected distances [Å] and angles [°]: Ir-C1 2.145(2), Ir-C10 2.134(2), Ir-C17 2.069(2), Ir-C28 2.043(2), Ir-N5 2.065(2), Ir-N6 2.044(2), C28-Ir1-N6 79.75(9), C28-Ir1-N5 92.85(9), N6-Ir1-N5 172.41(8), C28-Ir1-C17 80.25(9), N6-Ir1-C17 98.14(9), N5-Ir1-C17 78.80(10), C28-Ir1-C10 100.25(9), N6-Ir1-C10 90.65(9), N5-Ir1-C10 92.33(9), C17-Ir1-C10 171.12(10), C28-Ir1-C1 163.02(9), N6-Ir1-C1 98.16(9), N5-Ir1-C1 88.43(9), C17-Ir1-C1 83.40(9), C10-Ir1-C1 96.61(9).

All the presented iridium structures (Figure 2, Figure 3, Figure 4, Figure 5 and Figure 6) show some common features: the iridium atom is coordinated by a bis-NHC ligand and two phenylpyridine ligands, and the four carbon atoms and the two nitrogen atoms linked to iridium form a distorted octahedral geometry. It is notable that the carbene carbon atoms are in trans positions to the deprotonated carbon atoms of the ppy ligands and the two nitrogen atoms are in all cases also in trans positions related to the central iridium. The Ir-C(carbene) distances are in the range of 2.09 and 2.16 Å. The Ir-C distances (2.04 to 2.07 Å) and the Ir-N distances (2.03 to 2.07) of the ppy ligands are nearly the same in all cases. The ppy ligands show in all complexes very similar N-Ir-C bite angles between 78.8 and 80.4°. Only one remarkable difference could be observed for the bis-NHC systems. The C-Ir-C angles in the C1 ligands is between 83.9 and 84.6°, while it is much more open for the C2 ligands with angles between 95.4 and 96.6°, a significant difference of about 10° between the C1 and the C2 systems.

### 2.3. Photophysical Properties

**Figure 7:**
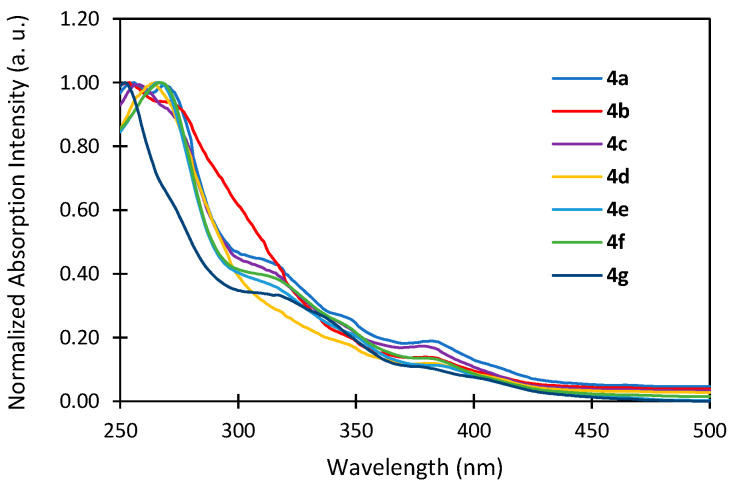
Normalized absorption spectra of complexes **4a** to **4g** in CH_2_Cl_2_ at 298 K.

All complexes have been photophysically investigated at standard pressure in CH_2_Cl_2_ at 298 K. The absorption spectra of complexes **4a** to **4g** in CH_2_Cl_2_ at 298 K are shown in Figure 7. The strong absorption bands below 300 nm correspond to spin-allowed ^1^π→π* electronic ligand-centered (LC) transitions [71]. The structureless bands at 300–360 nm are attributed to ppy-to-ppy ^1^π→π* ligand-centered charge transfer (LLCT) and Ir-to-ppy metal-to-ligand charge transfer (MLCT) transitions [71,72]. The lowest-lying bands in the visible region can be assigned to both singlet and triplet MLCT transitions [71,72,73]. No significant difference in the absorption spectra of complexes **4a** to **4g** can be found in the CH_2_Cl_2_ solution.

**Figure 8:**
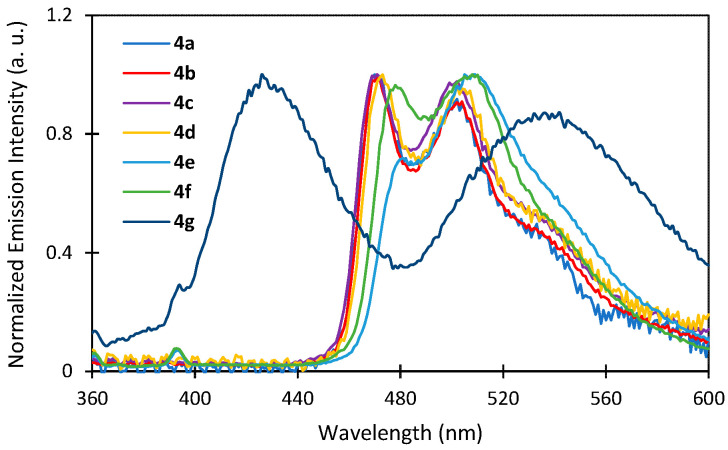
Normalized emission spectra of iridium complexes **4a**–**4g** in CH_2_Cl_2_ at 298 K (λ_ex_ = 360 nm).

The luminescence spectra of complexes **4a** to **4f** in CH_2_Cl_2_ at 298 K are shown in Figure 8 and their photophysical data are summarized in Table 1. After excitation at 360 nm, all the complexes, except **4g**, showed similar blue–green emission, with two maximum wavelengths around 470 and 500 nm, respectively. The neutral iridium complex **4g** displayed a dual blue and green emission, with two maxima at 425 nm and 535 nm. All the complexes showed vibronically structured phosphorescence spectra in the CH_2_Cl_2_ solution at room temperature, which indicated that the emissive excited state had both LC π→π* and MLCT characters [72]. 

The relative emission quantum yields (Φ_PL_) of these complexes in CH_2_Cl_2_ at room temperature were measured by using POPOP and quinine sulfate in 1 N H_2_SO_4_ as standard references (Φ_PL_ = 97% and Φ_PL_ = 54%, respectively) [74]. They are in the range of Φ_PL_ = 1.1–1.6% for the C1 family (**4a** to **4d**) and fall to 0.2% for both the C2 and C0 systems (**4e–4g**) (Table 1), suggesting a strong dependence of the quantum yields of the iridium complexes according to the used bis-NHC or NHC ligands. In addition, the excited-state lifetimes of complexes **4a** to **4d** are in the range of 166.4–190.7 ns, indicating the phosphorescence nature of the emissions.

### 2.4. Subcellular Localization

**Figure 9:**
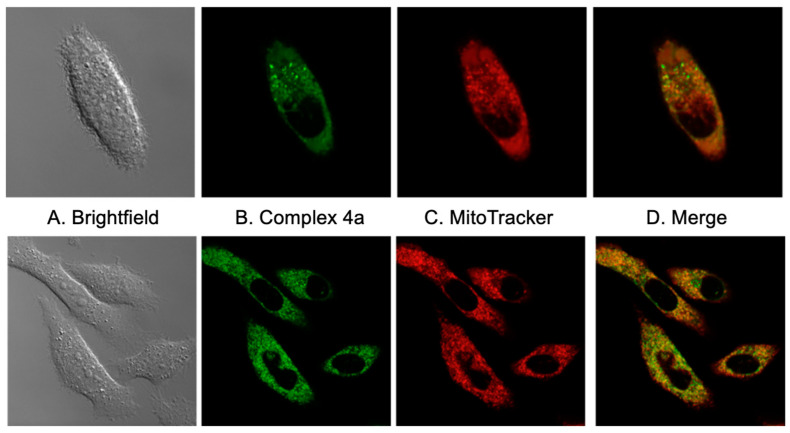
Determination of mitochondrial subcellular localization of complex **4a** by confocal microscopy. PC-3 cells were incubated with complex **4a** (500 nM) for 24 h and then co-incubated with MitoTracker Deep Red (200 nM) for 10 min at 37 °C. Iridium complex **4a** was excited at 720 nm (two-photon) and emission was collected at 440–544 nm. MitoTracker was excited at 633 nm and the emission was collected at 644–700 nm. The full image for zoom 4 (**up**) was 53.14 μm × 53.14 μm and 106.27 μm × 106.27 μm for zoom 2 (**down**).

The intracellular localization of complex **4a** was studied by confocal laser scanning microscopy. The treatment of PC-3 (human prostate) cells with complex **4a** led to the development of intense fluorescence in the cytoplasm (Figure 9B). Specific subcellular location was investigated by staining the organelles with the specific fluorescent probe MitoTracker Deep Red (Figure 9C). As shown in Figure 9D, complex **4a** localized mainly to the mitochondria with a Pearson colocalization coefficient value of 88%. However, an accumulation of complex **4a** in lysosomes or the endoplasmic reticulum cannot be excluded. Taken together, and based on the photophysical data, these results show that the C1 series of iridium complexes have potential as mitochondria-targeting agents. 

### 2.5. In Vitro Cytotoxicity

The in vitro cytotoxicity of iridium(III) complexes **4a**–**4g** was first evaluated against cancer cell lines PC-3 (human prostate) and T24 (human bladder) and the non-cancerous NIH-3T3 cell line (murine fibroblasts) using colorimetric MTT tests after 48 h of treatment. The dimeric iridium precursor [IrCl(ppy)_2_]_2_ was used as reference and the results are summarized in Table S1 (Supplementary Materials) in the supporting information file. All the complexes displayed high anti-proliferative activity against the two tested cancer cell lines (PC-3 and T24), with GI_50_ values in the nanomolar range from 0.25 to 0.95 μM. The complexes were also evaluated for their cytotoxicity towards healthy NIH-3T3 cells, giving access to the selectivity index (SI). They present moderate to good selectivities for cancer cells, since they were found to be less active against non-cancerous NIH-3T3 cells than cancer cells (T24 and PC-3), with SI values ranging from 4.5 to 17.3. No linear correlation between lipophilicity and anti-cancer activity was found for the iridium(III) complexes **4a**–**4g**. For the following biological studies, we focused on the C1 series. 

The cytotoxicity and photodynamic activities of the four *N*-heterocyclic carbene-iridium complexes **4a**–**4d** were further examined in two non-cancerous cell models (NIH-3T3 fibroblasts and MC3T3 osteoblasts) and in a panel of five cancer cell models (T24 bladder, PC-3 prostate, MCF7/FasR breast, A549 lung and HeLa cervix), exhibiting a representative array of gene mutations found in cancer cells including TP53 (cancer protein p53), PTEN (phosphatase and tensin homolog), HRAS and KRAS (rat sarcoma virus) family members, PIK3CA (phosphatidylinositol-4, 5-bisphosphate 3-kinase, catalytic subunit alpha) or CDKN2A (cyclin-dependent kinase inhibitor 2A). 

**Figure 10:**
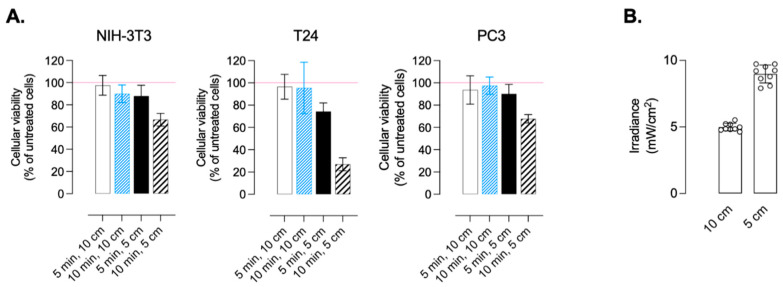
Validation of the conditions of irradiation (**A**) NIH-3T3, T24 and PC-3 cells were incubated for 72 h without drug, irradiated or not for 5 or 10 min, at 5 or 10 cm of distance. Cell death was assessed by MTT assay. Data are expressed as percentage (%) of cell viability compared with non-irradiated cells and are the means of 3 independent experiments. (**B**) Irradiance was quantified at a distance of 5 or 10 cm between the cell layer and the light source (458 nm) and expressed per unit of surface (mW/cm^2^).

To ascertain that irradiation conditions did not per se affect cell viability, NIH-3T3, T24 and PC-3 cells were irradiated or not at 458 nm for different times (5 and 10 min) at different distances between the cell layer and the light source (5 and 10 cm). Cell viability was measured after 72 h of incubation. A minimal toxicity was observed for a distance of 10 cm between the light source and the cells, with no significant difference in toxicity between 5 or 10 min of irradiation (Figure 10A). Based on these results, all further experiments were conducted with the following irradiation conditions: 10 min of irradiation at a distance of 10 cm. As shown in Figure 10B, under these conditions, the measured irradiance was 5.0 mW/cm^2^ after the illumination of 48-well cell culture plates.

The GI_50_ values of complexes **4a**–**4d** under dark and light irradiation conditions are summarized in Table 2. First, as expected, the dimeric iridium precursor [IrCl(ppy)_2_]_2_ showed no cytotoxic effect, with GI_50_ values ranging from 26 to 40 µM in the dark and 14 to 22 µM after irradiation in NIH-3T3, T24 and PC-3 cell lines. In the dark, the complexes **4a**–**4d** showed modest toxicity towards non-cancerous NIH-3T3 and MC3T3 cells, with GI_50_ values ranging from 0.979 to 3.67 µM (mean GI_50_ = 2.2 µM). In cancer cells, the complexes showed greater efficiency, with GI_50_ values ranging from 211 nM to 1.54 µM depending on the cell type (T24, mean GI_50_ = 352 nM, mean SI = 6.5-fold; PC-3, mean GI_50_ = 334 nM, mean SI = 7.2-fold; MCF7, mean GI_50_ = 259 nM, mean SI = 8.8-fold; A549, mean GI_50_ = 657 nM, mean SI = 4.8-fold); HeLa cells being the least sensitive (mean GI_50_ = 987 nM, mean SI = 2.5-fold). Overall, we observed a six-fold higher sensitivity of cancer cells compared with NIH-3T3 and MC3T3 cells in the dark treatment condition (SI Dark vs. NIH-3T3 and SI Dark vs. MC3T3, Table 2). Interestingly, the complexes did not show statistically significant photosensitizing effects against non-cancerous cells, with a mean GI_50_ = 1.9 µM (0.704–2.79 µM) reflected by a mean photosensitizing index (PI) of 1.15. On the contrary, we observed a notable photosensitizing effect for all complexes towards cancer cells, with GI_50_ values ranging from 50 nM to 817 nM depending on the cell type (T24, GI_50_ ranging from 90 to 165 nM, mean PI = 3.3-fold; PC-3, GI_50_ ranging from 57 to 157 nM, mean PI = 3.5-fold; MCF7, GI_50_ ranging from 50 to 117 nM, mean PI = 3.9-fold; A549, GI_50_ ranging from 84 to 617 nM, mean PI = 3.1-fold); HeLa cells being the least sensitive (GI_50_ ranging from 275 to 817 nM, mean PI = 2.1-fold).

Collectively, all complexes showed strong specificity for cancer cells under irradiation compared with non-cancerous cells (Table 2). Complex **4b** and complex **4d** showed an average selectivity index (SI) of 18 and 13.2 against NIH-3T3 cells and 8.3 and 8.6 against MC3T3 cells, respectively. Complex **4c** exhibited an average SI of 24.3 against NIH-3T3 cells and 12.1 against MC3T3 cells. Complex **4a** showed the highest specificity for cancer cells with an average SI of 34.0 (5.3–55.8) against NIH-3T3 cells and 21.2 (3.3–34.8) against MC3T3 cells.

### 2.6. Mechanistic Studies

**Figure 11:**
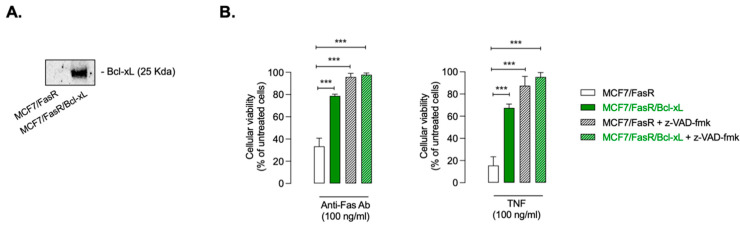
Bcl-xL and z-VAD-fmk inhibited by Fas- or TNFα-induced cell death in MCF7 cells. (**A**) Cellular proteins from MCF7/FasR and MCF7/FasR/Bcl-xL cells were separated by SDS-PAGE and immunoblotted using anti-Bcl-xL antibody. (**B**) MCF7/FasR and MCF7/FasR/Bcl-xL treated with 100 ng/mL anti-Fas or 100 ng/mL TNFα for 72 h in presence or in absence of 10 µM pan-caspase inhibitor z-VAD-fmk. Cell death was assessed by MTT assay. Results are means ± SD of 3 independent experiments (***, *p* < 0.0001).

To determine the mechanism of cell death, we used MCF7 cells transfected to express the human Fas receptor (FasR) or doubly transfected to express the human Fas receptor and human Bcl-xL [75]. Since MCF7 cells express the TNF receptor endogenously, this allowed the study of both Fas- and TNFα-mediated apoptosis in the same cell model to assess whether cell death was dependent on the Bcl-2 family of mitochondrial proteins such as Bcl-xL. As shown in Figure 11A, MCF7/FasR/Bcl-xL cells overexpress the Bcl-xL protein [76]. Fas or TNFα cross-linking was associated with a 65–85% reduction in cell viability, which was markedly inhibited by Bcl-xL overexpression, but not completely (Figure 11B). This is consistent with findings that death receptors such as FasR and TNFR can mediate apoptosis through both the extrinsic pathway (mitochondria-independent by directly activating executioner caspases via upstream caspase-8) and the intrinsic (mitochondrial) pathway controlled by the Bcl-2 family of proteins [75,77,78]. Having demonstrated that Bcl-xL was functional in our cell model, we next examined the effect of the pan-caspase inhibitor z-VAD-fmk on Fas- and TNFα-mediated cell death. Treatment with z-VAD-fmk completely abolished cell death induced by the Fas antibody and TNFα in both MCF7/FasR and MCF7/FasR/Bcl-xL, indicating that our pan-caspase inhibitor is fully functional in this cell model (Figure 11B).

**Figure 12:**
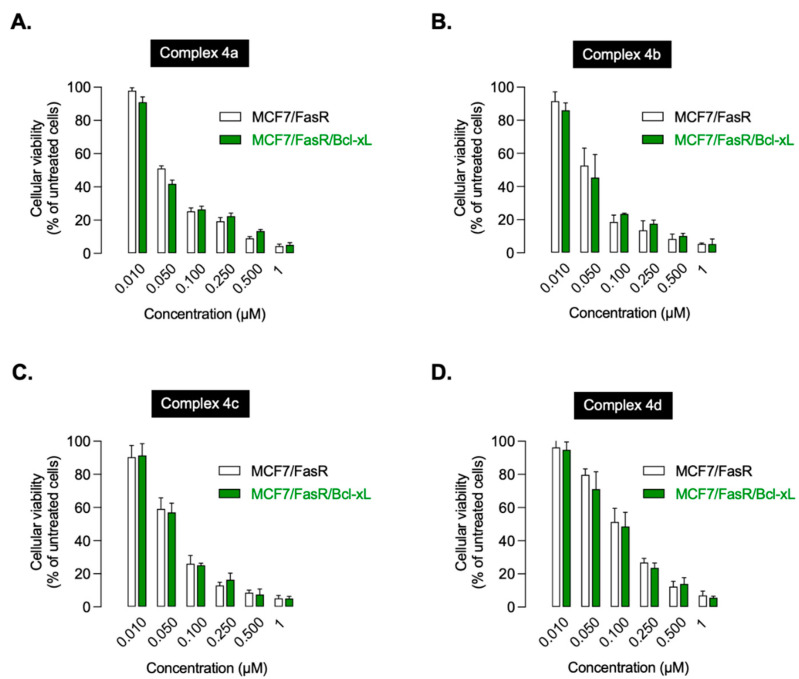
Bcl-xL does not inhibit cell death induced by complexes **4a**–**d**. MCF7/FasR and MCF7/FasR/Bcl-xL cells were irradiated at 458 nM in the presence of the indicated concentration of complex **4a** (**A**), complex **4b** (**B**), complex **4c** (**C**) and complex **4d** (**D**). After 72 h of incubation, cell death was assessed by MTT assay. Data are expressed as percent (%) of cell viability compared with untreated cells and are the means of at least 3 independent experiments (*p* values not significant).

Mitochondrial outer membrane permeabilization (MOMP), which leads to rapid cell death through the release of mitochondrial proteins (including cytochrome *c*) that activate executioner caspases, is the critical event in intrinsic apoptosis [79]. This is an irreversible event, which can only be antagonized by the anti-apoptotic members of the Bcl-2 family, including Bcl-xL, the only anti-apoptotic Bcl-2 member exclusively associated to the mitochondrial outer membrane [80]. We then assessed whether the cell death induced by our complexes could be abrogated by the overexpression of Bcl-xL. As shown in Figure 12, the effects of complexes **4a** to **4d** with respect to loss of cell viability were not affected by Bcl-xL, regardless of the drug concentration used (from 10 nM to 1 µM), suggesting that cell death induced by our complexes is not based on a Bcl-xL-dependent mitochondrial mechanism.

**Figure 13:**
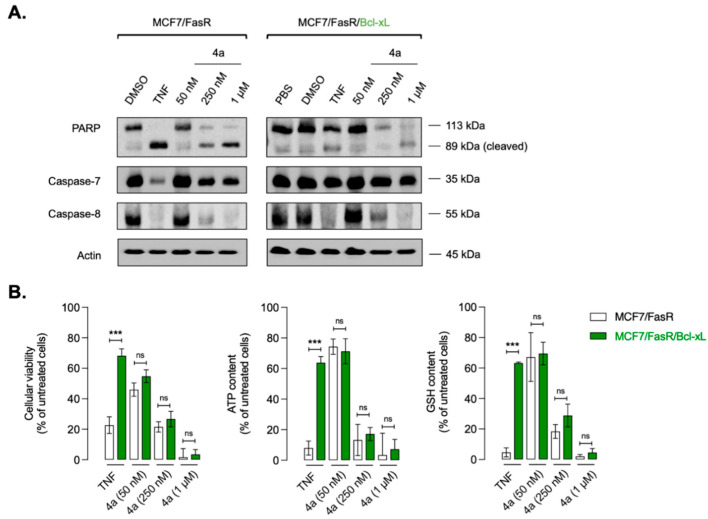
Evaluation of apoptotic biomarkers. MCF7/FasR and MCF7/FasR/Bcl-xL cells were irradiated at 458 nm in presence of the indicated concentration of complex **4a** or treated with 100 n/mL TNFα for 72 h. (**A**) Cytosolic extracts from MCF7/FasR and MCF7/FasR/Bcl-xL cells were separated by SDS-PAGE and probed with anti-PARP, anti-caspase-7 and anti-caspase-8. Migrations indicated are: full-length PARP p113, cleaved PARP p89, full-length caspase-7 and full-length caspase-8. (**B**) MTT, ATP and GSH assays were evaluated in duplicate samples. Data are expressed as percent (%) of untreated cells and are the means of 3 independent experiments (***, *p* < 0.0001, NS, not significant).

Complex **4a** presented the best profile in terms of specificity, with GI_50_ values ranging from 50 to 107 nM in our different cancer models (bladder, prostate, breast, lung), with the exception of ovarian cells, which were not very sensitive (GI_50_ of 525 nM). Its effects on apoptotic biochemical markers by Western blotting analysis in MCF7/FasR cells and its Bcl-xL-overexpressed counterpart in comparison with the apoptotic inducer TNFα were examined (Figure 13). Cleavage-mediated inactivation of DNA repair enzyme poly(ADP-ribose) polymerase (PARP) by executioner caspases (e.g., caspase-3, caspase-7) has been considered as a marker of apoptotic cell death [81]. The treatment with TNFα was associated with the proteolytic cleavage of PARP (decreased in 113 kDa full-length, increased in apoptotic 89 kDa fragment) in MCF7/FasR cells (Figure 13A). Since the MCF7 cell line lacks caspase-3 due to the functional deletion of the *CASP-3* gene [82], caspase-7 is the only executioner caspase in these cells, as evidenced by the decreased content in the 35 kDa full-length pro-caspase-7 observed upon TNFα treatment (Figure 13A). The upstream caspase-8 that can directly process caspase-7 (extrinsic pathway) was also activated (loss of 55 kDa full-length pro-caspase-8). As expected, the overexpression of Bcl-xL rendered MCF7/FasR cells resistant to TNFα-induced PARP and caspase-7 processing but did not affect caspase-8 enzymatic activity, because Bcl-xL functions downstream of caspase-8 (Figure 13A). Regarding complex **4a**, when used at its GI_50_ concentration (50 nM), no processing of PARP, caspase-7 and caspase-8 were observed in both MCF7/FasR and MCF7/FasR/Bcl-xL (Figure 13A). However, when used at a much higher concentration (250 nM and 1 µM), treatment with complex **4a** in MCF7/FasR was associated with cleavage of PARP and processing of caspase-7 and caspase-8, suggesting the activation of the extrinsic (mitochondria-independent) pathway. As shown in Figure 13A, Bcl-xL overexpression did not appear to affect PARP, caspase-7 and caspase-8 processing. These data suggest that treatment with high doses (250 nM or 1 µM, respectively, 5- and 20-fold the GI_50_ concentration) of complex **4a** is associated with the classical biochemical markers of apoptosis, namely cleavage of PARP and upstream caspases’ activation, in contrast to the GI_50_ concentration. Importantly, the overexpression of Bcl-xL had no effect on apoptotic markers, regardless of the concentration of complex **4a** used, implying that a MOMP-dependent mechanism is unlikely to be involved. In parallel experiments, cell viability and quantification of ATP and GSH levels were assessed. In agreement with data presented in Figure 11B and Figure 12A, TNFα-induced loss of cell viability was strongly inhibited by Bcl-xL, whereas complex **4a**-induced cell death was not affected by Bcl-xL overexpression (Figure 13B). The early transient elevation of cytosolic ATP level is a necessary condition for the apoptotic cell death process, followed by a drop that correlates with the time course of cell death [83]. Under our experimental conditions (72 h of treatment), the loss of ATP observed in cells treated with an increasing concentration of complex **4a** cannot distinguish the type of cell death, the ATP measurement then acting as a surrogate marker of viability (Figure 13B). The level of reduced GSH, which plays a central role in the defense of cells against ROS in apoptotic and non-apoptotic cell death, was decreased by TNFα and all doses of complex **4a**, yet Bcl-xL overexpression did not affect the decrease in GSH induced by complex **4a** (Figure 13B).

**Figure 14:**
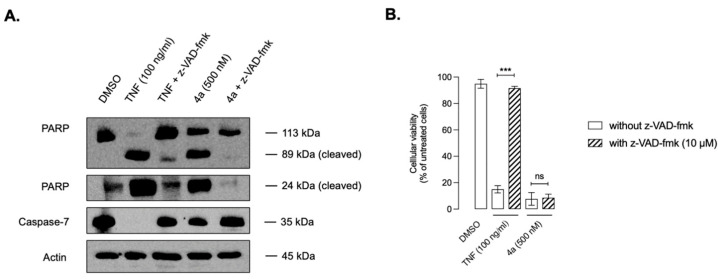
Evaluation of apoptotic biomarkers. MCF7/FasR cells were irradiated at 458 nm with 500 nM of complex **4a** or 100 n/mL TNFα for 72 h in presence or not of 10 µM z-VAD-fmk. (**A**) Cytosolic extracts from MCF7/FasR cells were separated by SDS-PAGE and probed with full-length and p24 cleaved anti-PARP and anti-caspase-7. Migrations indicated are: full-length PARP p113, cleaved PARP p89, cleaved PARP p24 and full-length caspase-7. (**B**) Cell viability was evaluated in duplicate samples. Data are expressed as percent (%) of untreated cells and are the means of 3 independent experiments (***, *p* < 0.0001, NS, not significant).

We next evaluated the effect of the pan-caspase inhibitor z-VAD-fmk on caspase signaling. As expected, the treatment with z-VAD-fmk rendered MCF7/FasR cells resistant to TNFα-induced PARP and caspase-7 processing (Figure 14A) and to the loss of cell viability (Figure 14B). In line with data shown in Figure 13, a high dose of complex **4a** (500 nM or 10-fold the GI_50_) triggered a significant loss of procaspase-7 expression correlated with the activation of PARP (decreased p113 form and appearance of both p89 and p24 cleaved forms). As expected, z-VAD-fmk remarkably inhibited the processing of both caspase-7 and PARP (Figure 14A) yet could not prevent cell death induced by complex **4a** (Figure 14B).

Collectively, the data shown in Figure 13 and Figure 14 suggest activation of an unexpected extrinsic (mitochondria-independent) apoptotic signaling (caspase-8/caspase-7/PARP), with a high concentration of complex **4a** (5- to 20-fold the GI_50_ concentration). As anticipated, while this sequential activation of caspases cannot be blocked by Bcl-xL, treatment with z-VAD-fmk is able to completely block this extrinsic apoptotic signaling. Such activation of the extrinsic apoptotic signaling might be the consequence of a bystander effect of necrotic dying cells that could release factors activating the TNF superfamily of receptors, leading to caspase-8 activation, and subsequent activation of caspase-7, leading to PARP cleavage. Alternatively, it could also be a consequence of a potential feedback amplification of upstream apoptosis signaling directly via caspase-8 activation independently of TNF/TNF receptor family members’ interaction. Importantly, regardless of the mechanism of action leading to caspase-8 activation, z-VAD-fmk, while blocking the activation of this signaling pathway, did not have any effect on cell viability, suggesting a futile activation of apoptosis signaling driven by caspase-8 and not a major determinant of the behavior of complex **4a**-exposed cells, which has been reported in the past with DNA damaging agents [84].

## 3. Material and Methods

### 3.1. Chemistry

#### 3.1.1. General Information

**Figure 15:**
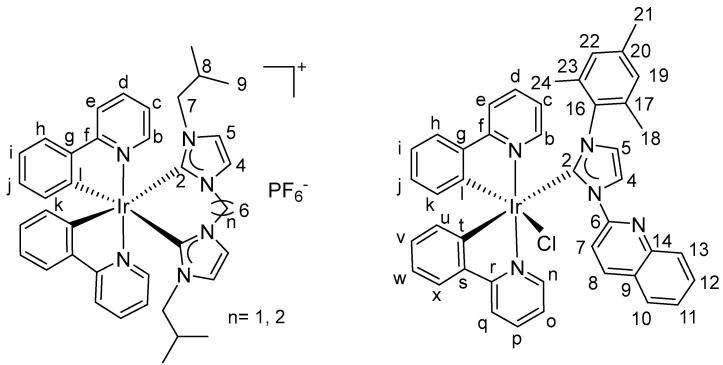
Numbering of H (^1^H NMR) and C (^13^C NMR) used for the NMR attributions.

Unless stated, all reactions were performed under air. 3-mesityl-1-(quinolin-2-yl)-1*H*-imidazol-3-ium chloride (**3g**) [68] and [IrCl(ppy)_2_]_2_ [67] were prepared according to the literature procedures. All other reagents were used as received from commercial suppliers. Reactions involving silver compounds were performed with the exclusion of light. ^1^H (400 or 500 MHz) and ^13^C NMR spectra (100 or 125 MHz) were recorded at 298 K on Bruker AV400 or Bruker Avance 500 spectrometers in CDCl_3_, CD_3_CN and DMSO-*d_6_* as solvents. All chemical shifts for ^1^H and ^13^C are relative to TMS using ^1^H (residual) or ^13^C chemical shifts of the solvent as a secondary standard. The temperature was set at 298 K. All the ^1^H and ^13^C signals were assigned on the basis of chemical shifts, spin–spin coupling constants, splitting patterns and signal intensities and by using ^1^H-^1^H COSY45, ^1^H-^13^C HSQC, ^1^H-^13^C HMBC, ^13^C and ^1^H experiments. Gradient-enhanced ^1^H COSY45 experiments were realized, including 2 scans per increment. ^1^H-^13^C correlation spectra, using a gradient-enhanced HSQC sequence (delay was optimized for ^1^J_CH_ of 145 Hz), were obtained with 2 scans per increment. Gradient-enhanced HMBC experiments were performed, allowing 62.5 ms for long-range coupling evolution (8 scans were accumulated). Typically, 1024 t2 data points were collected for 256 t1 increments. The NMR attribution follows the numbering scheme presented in Figure 15. Elemental analyses were carried out by the Service de Micro-analyse du Laboratoire de Chimie de Coordination (Toulouse). Mass spectrometry (MS) and high resolution mass spectrometry (HRMS) analysis were performed with a Xévo G2 QTOF Waters spectrometer using electrospray ionization (ESI) by the Service de Spectrométrie de Masse de l’ICT (Toulouse).

#### 3.1.2. Preparation of Imidazolium Salts

*1,1′-methylenebis(3-isopropyl-1H-imidazol-3-ium) dibromide* (**3a**). A flask was charged with a mixture of imidazole (1.36 g, 20 mmol), tetrabutylammonium bromide (193 mg, 0.6 mmol) and powdered potassium hydroxide (2.24 g, 40 mmol) and stirred for 1 h at room temperature. To the resulting liquid was slowly added dibromomethane (0.7 mL, 10 mmol). After 5 h, a solid was obtained. Purification of the crude product was performed by sublimation at 180 °C to yield a white product (885 mg, 60% yield): ^1^H NMR (400 MHz, CDCl_3_): *δ* 7.67 (s, 2H, H_2_), 7.14 (s, 2H, H_5_), 7.14 (s, 2H, H_4_), 6.02 (s, 2H, H_6_). 1,1′-methylene bis(1*H*-imidazole) (50 mg, 0.34 mmol) was dissolved in 2 mL of 2-bromopropane and the reaction mixture was refluxed for 48 h. After cooling down to room temperature, the precipitate formed was filtered, washed with Et_2_O and dried under vacuum to obtain 1,1′-methylene bis(1*H*-imidazole) as a white solid (50 mg, 38% yield). Anal. Calcd. for C_13_H_22_Br_2_N_4_: C, 39.61; H, 5.63; N, 14.21. Found: C, 39.45; H, 5.54; N, 14.05. ^1^H NMR (400 MHz, DMSO-*d_6_*): *δ* 9.68 (s, 2H, H_2_), 8.12 (d, *J* = 1.9 Hz, 2H, H_5_), 8.06 (d, *J* = 1.9 Hz, 2H, H_4_), 6.67 (s, 2H, H_6_), 4.74–4.68 (m, 2H, H_7_), 1.51 (d, 12H, *J* = 6.4 Hz, H_8_). ^13^C NMR (100 MHz, DMSO-*d_6_*): *δ* 137.0 (2C, C_2_), 122.9 (2C, C_5_), 121.8 (2C, C_4_), 58.5 (1C, C_6_), 53.3 (2C, C_7_), 22.6 (4C, C_8_). MS (ES^+^): calcd. for C_13_H_22_BrN_4_ 313.10; found m/z = 312.92 [M − Br^−^]^+^. Calcd. for C_13_H_21_N_4_ 233.18; found 233.17 [M − 2Br^−^ − H^+^]^+^.

*1,1′-methylenebis(3-isobutyl-1H-imidazol-3-ium) dibromide* (**3b**). 1,1′-methylene bis(1*H*-imidazole) (100 mg, 0.67 mmol) was dissolved in 2 mL of 1-bromo-2-methylpropane and the reaction mixture was refluxed for 48 h. After cooling down to room temperature, the precipitate formed was filtered, washed with Et_2_O and dried under vacuum to obtain a white solid (195 mg, 69% yield). Anal. Calcd. for C_15_H_26_Br_2_N_4_: C, 42.67; H, 6.21; N, 13.27. Found: C, 42.64; H, 6.13; N, 13.10. ^1^H NMR (400 MHz, DMSO-*d_6_*): *δ* 9.62 (s, 2H, H_2_), 8.11 (s, 2H, H_5_), 7.90 (s, 2H, H_4_), 6.74 (s, 2H, H_6_), 4.09 (d, 4H, *J* = 7.2 Hz, H_7_), 2.16–2.07 (m, 2H, H_8_), 0.89 (d, 12H, *J* = 6.4 Hz, H_9_). ^13^C NMR (100 MHz, DMSO-*d_6_*): *δ* 138.1 (2C, C_2_), 124.0 (2C, C_5_), 122.7 (2C, C_4_), 58.7 (1C, C_6_), 56.4 (2C, C_7_), 29.0 (2C, C_8_), 19.6 (4C, C_9_). MS (ES^+^): calcd. for C_15_H_26_BrN_4_ 341.13; found 341.25 [M − Br^−^]^+^. Calcd. for C_15_H_25_N_4_ 261.21; found 261.25 [M − 2Br^−^ − H^+^]^+^.

*1,1′-methylenebis(3-benzyl-1H-imidazol-3-ium) dichloride* (**3c**). A mixture of 1,1′-methylene bis(1*H*-imidazole) (100 mg, 0.67 mmol) and benzyl chloride (188 mg, 1.48 mmol) was refluxed in 5 mL of MeCN for 10 h. After cooling to room temperature, the precipitate formed was filtered, washed with Et_2_O and dried under vacuum to obtain a pale-yellow solid (190 mg, 71% yield). Anal. Calcd. for C_21_H_22_Cl_2_N_4_: C, 62.85; H, 5.53; N, 13.96. Found: C, 62.56; H, 5.34; N, 13.72. ^1^H NMR (400 MHz, DMSO-*d_6_*): *δ* 9.98 (s, 2H, H_2_), 8.26 (s, 2H, H_5_), 7.92 (s, 2H, H_4_), 7.52–7.34 (m, 10H, H_bn_), 6.83 (s, 2H, H_6_), 5.51 (s, 4H, H_7_). ^13^C NMR (100 MHz, DMSO-*d_6_*): *δ* 138.4 (2C, C_2_), 134.7 (2C, C_bn_), 129.5 (4C, C_bn_), 129.4 (2C, C_bn_), 129.0 (4C, C_bn_), 123.5 (2C, C_5_), 123.1 (2C, C_4_), 58.5 (1C, C_6_), 52.7 (2C, C_7_). MS (ES^+^): calcd. for C_21_H_21_N_4_ 329.18; found 329.42 [M − 2Cl^−^ − H^+^]^+^.

*1,1′-methylenebis(3-(4-(methylthio)phenyl)-1H-imidazol-3-ium) dibromide* (**3d**). Imidazole (3.24 g, 47.6 mmol), 4-bromothioanisole (4.83 g, 23.8 mmol), K_2_CO_3_ (3.29 g, 23.8 mmol) and a catalytic amount of CuSO_4_ were stirred in a closed pressure flask at 205 °C for 10 h. After cooling to room temperature, the crude product was extracted by CH_2_Cl_2_, filtered and evaporated to dryness. The resulting residue was washed three times with Et_2_O (20 mL) to give 1-(4-methylthiophenyl)-1*H*-imidazole as a grey solid (3.6 g, 80% yield). ^1^H NMR (400 MHz, CDCl_3_): *δ* 7.84 (t, 1H, *J* = 1.2 Hz, H_2_), 7.38–7.32 (m, 4H, H_Ph_), 7.27 (t, 1H, *J* = 1.2 Hz, H_5_), 7.22 (t, 1H, *J* = 1.2 Hz, H_4_), 2.54 (s, 3H, H_SMe_). 1-(4-(methylthiophenyl)-1*H*-imidazole (200 mg, 1.05 mmol) was dissolved in 2 mL of dibromomethane and the reaction mixture was refluxed for 10 h. After cooling down, the precipitate formed was filtered, washed with Et_2_O and dried under vacuum to obtain a white solid (255 mg, 44% yield). Anal. Calcd. for C_21_H_22_Br_2_N_4_S_2_: C, 45.50; H, 4.00; N, 10.11. Found: C, 45.29; H, 3.93; N, 10.50. ^1^H NMR (400 MHz, DMSO-*d_6_*): *δ* 10.31 (s, 2H, H_2_), 8.42 (s, 2H, H_5_), 8.38 (s, 2H, H_4_), 7.76 (d, 4H, *J* = 8.8 Hz, H_Ph_), 7.56 (d, 4H, *J* = 8.8 Hz, H_Ph_), 6.90 (s, 2H, H_6_), 2.56 (s, 6H, H_SMe_). ^13^C NMR (100 MHz, DMSO-*d_6_*): *δ* 141.9 (2C, C_Ph_), 137.6 (2C, C_2_), 131.6 (2C, C_Ph_), 127.2 (4C, C_Ph_), 123.4 (2C, C_5_), 122.9 (4C, C_Ph_), 122.1 (2C, C_4_), 58.9 (1C, C_6_), 14.9 (2C, C_SMe_). MS (ES^+^): calcd. for C_21_H_22_BrN_4_S_2_ 473.05; found 473.25 [M − Br^−^]^+^. Calcd. for C_21_H_21_N_4_S_2_ 393.12; found 393.33 [M − 2Br^−^ − H^+^]^+^.

*1,1′-(1,2-ethanediyl)bis[(3-benzyl-1H-imidazol-3-ium) dibromide* (**3e**). A mixture of imidazole (6.201 g, 91 mmol) and K_2_CO_3_ (15.420 g, 112 mmol) in 60 mL of DMF was stirred for 20 min, then benzyl chloride (12.6 mL, 109 mmol) was slowly added to the solution. The reaction mixture was stirred at 50 °C for 2 h. After cooling to room temperature, water (100 mL) was added. The aqueous layer was extracted twice with EtOAc (100 mL) and the combined organic layers were washed with water and brine and dried over Na_2_SO_4_. The solvent was evaporated to give 1-benzyl-1*H*-imidazole as a beige solid (7.910 g, 55% yield). ^1^H NMR (400 MHz, CD_3_Cl): *δ* 7.57 (s, 1H, H_2_), 7.40–7.33 (m, 3H, H_Bn_), 7.17 (m, 2H, H_Bn_), 7.11 (d, *J* = 1.8 Hz, 1H, H_5_), 6.92 (d, *J* = 1.8 Hz, 1H, H_4_), 5.14 (s, 2H, H_7_). A mixture of 1-benzyl-1*H*-imidazole (105 mg, 0.64 mmol) and 1,2-dibromoethane (63.9 mg, 0.34 mmol) in 5 mL of toluene was stirred under reflux for 24 h. After cooling to room temperature, the solvent was evaporated, the crude was washed with Et_2_O, filtered and dried under vacuum to obtain a beige solid (105 mg, 66% yield). ^1^H NMR (400 MHz, DMSO-*d*_6_): *δ* 9.32 (s, 2H, H_2_), 7.83 (s, 2H, H_5_), 7.72 (s, 2H, H_4_), 7.48–7.36 (m, 10H, H_bn_), 5.44 (s, 4H, H_6_), 4.75 (s, 4H, H_7_). ^13^C NMR (100 MHz, DMSO-*d*_6_): *δ* 137.2 (2C, C_2_), 135.0 (2C, C_bn_), 129.5 (4C, C_bn_), 129.3 (2C, C_bn_), 128.8 (4C, C_bn_), 123.4 (2C, C_5_), 123.3 (2C, C_4_), 52.5 (2C, C_6_), 48.9 (2C, C_7_). MS (ES^+^): calcd. for C_22_H_24_BrN_4_ 423.12; found 423.42 [M − Br^−^]^+^. Calcd. for C_22_H_23_N_4_ 343.19; found 343.75 [M − 2Br^−^ − H^+^]^+^.

*1,1′-(1,2-ethanediyl)bis[(3-isobutyl-1H-imidazol-3-ium) dibromide* (**3f**). A mixture of imidazole (497 mg, 7.3 mmol) and potassium hydroxide (617.2 mg, 11 mmol) was dissolved in 5 mL of DMSO and stirred for 30 min at room temperature. Then, isobutyl bromide (1000.3 mg, 7.3 mmol) was slowly added at 0 °C. After 2 h stirring at room temperature, the mixture was diluted with water and EtOAc. The organic layer was washed with brine and dried over Na_2_SO_4_. The solvent was evaporated to give 1-(2-methylpropyl)-1*H*-imidazole as a white solid (547 mg, 60% yield). ^1^H NMR (400 MHz, CDCl_3_): *δ* 7.46 (s, 1H, H_2_), 7.07 (s, 1H, H_5_), 6.89 (s, 1H, H_4_), 3.74 (d, *J* = 7.2 Hz, 2H, H_7_), 2.10–1.98 (m, 1H, H_8_), 0.93 (d, *J* = 6.7 Hz, 6H, H_9_). A mixture of 1-(2-methylpropyl)-1*H*-imidazole (123.5 mg, 1 mmol) and 1,2-dibromoethane (93.4 mg, 0.5 mmol) in 5 mL of toluene was stirred in a closed pressure flask at 110 °C for 72 h. After cooling to room temperature, the solvent was evaporated and the crude was dissolved in 10 mL of MeCN. The mixture was filtered and the solvent was removed. The solid was washed with a mixture of MeCN and Et_2_O (1:5) and dried under vacuum to afford a white solid (170 mg, 78% yield). ^1^H NMR (400 MHz, DMSO-*d*_6_): *δ* 9.06 (s, 2H, H_2_), 7.80 (s, 2H, H_5_), 7.70 (s, 2H, H_4_), 4.71 (s, 4H, H_6_), 3.98 (d, *J* = 7.1 Hz, 4H, H_7_), 2.10–1.95 (m, 2H, H_8_), 0.83 (d, *J* = 6.6 Hz, 12H, H_9_). ^13^C NMR (100 MHz, DMSO-*d*_6_): *δ* 137.1 (2C, C_2_), 123.8 (2C, C_5_), 123.0 (2C, C_4_), 56.1 (2C, C_6_), 48.9 (2C, C_7_), 29.1 (2C, C_8_), 19.5 (4C, C_9_). MS (ES^+^): calcd. for C_16_H_27_N_4_ 275.22; found 275.17 [M − 2Br^−^ − H^+^]^+^.

*3-mesityl-1-(quinolin-2-yl)-1H-imidazol-3-ium chloride* (**3g**). ^1^H NMR (400 MHz, CDCl_3_): *δ* 12.15 (s, 1H, H_2_), 9.43 (d, *J* = 8.8 Hz, 1H, H_8_), 9.07 (d, *J* = 1.7 Hz, 1H, H_5_), 8.59 (d, *J* = 8.8 Hz, 1H, H_7_), 8.08 (d, *J* = 8.4 Hz, 1H, H_13_), 7.97 (d, *J* = 8.2 Hz, 1H, H_10_), 7.88–7.79 (m, 1H, H_12_), 7.70–7.64 (m, 1H, H_11_), 7.38 (d, *J* = 1.7 Hz, 1H, H_4_), 7.07 (s, 2H, H_Mes_), 2.37 (s, 3H, CH_3_), 2.24 (s, 6H, CH_3_). ^13^C NMR (100 MHz, CDCl_3_): *δ* 146.0 (1C, C_6_), 144.9 (1C, C_14_), 141.8 (1C, C_8_), 141.5 (1C, C_Mes_), 138.0 (1C, C_2_), 134.0 (2C, C_Mes_), 131.3 (1C, C_12_), 130.7 (2C, C_Mes_), 130.1 (2C, C_Mes_), 128.7 (1C, C_13_), 128.6 (1C, C_9_), 128.2 (1C, C_10_), 128.1 (1C, C_11_), 123.7 (1C, C_7_), 119.8 (1C, C_5_), 114.1 (1C, C_4_), 21.2 (1C, C_Mes_), 17.9 (2C, C_Mes_).

#### 3.1.3. Synthesis of Iridium(III) Complexes

*Complex (***4a**). A mixture of **3a** (35 mg, 0.09 mmol), silver oxide (20.6 mg, 0.09 mmol) and [IrCl(ppy)_2_]_2_ (43 mg, 0.04 mmol) in 10 mL of MeCN was refluxed for 10 h. After cooling down to room temperature, an aqueous solution of NH_4_PF_6_ (200 mg in 10 mL deionized water) was slowly added into the reaction mixture under stirring, resulting in a yellow suspension. The suspension was then filtered and the resulting precipitate was washed with deionized water and dried under vacuum at 70 °C for 12 h. The crude product was purified by column chromatography on silica gel with CH_2_Cl_2_/MeOH (20:1) as the eluent, yielding a yellow solid (40 mg, 57% yield). Anal. Calcd. for C_35_H_36_F_6_IrN_6_P: C, 47.89; H, 4.13; N, 9.57. Found: C, 47.45; H, 4.04; N, 9.79. ^1^H NMR (400 MHz, CD_3_CN): *δ* 8.33 (d, 2H, *J* = 5.6 Hz, H_b_), 8.09 (d, 2H, *J* = 7.6 Hz, H_e_), 7.91 (ddd, 2H, *J* = 7.6, 7.6, 1.2 Hz, H_d_), 7.74 (d, 2H, *J* = 7.6 Hz, H_k_), 7.37 (d, 2H, *J* = 2.4 Hz, H_5_), 7.13–7.09 (m, 4H, H_c_/H_4_), 6.89 (ddd, 2H, *J* = 7.6, 7.6, 1.2 Hz, H_j_), 6.79 (ddd, 2H, *J* = 7.6, 7.6, 1.2 Hz, H_i_), 6.30 (d, 2H, *J* = 7.6 Hz, H_h_), 5.98 (s, 2H, H_6_), 4.16–4.10 (m, 2H, H_7_), 1.02 (d, 6H, *J* = 6.4 Hz, H_8_), 0.27 (d, 6H, *J* = 6.4 Hz, H_8′_). ^13^C NMR (100 MHz, CDCl_3_): *δ* 169.7 (2C, C_f_), 162.7 (2C, C_2_), 162.2 (2C, C_l_), 153.0 (2C, C_b_), 143.9 (2C, C_g_), 136.7 (2C, C_d_), 131.0 (2C, C_h_), 129.6 (2C, C_i_), 124.4 (2C, C_5_), 123.9 (2C, C_k_), 121.9 (2C, C_c_), 121.4 (2C, C_4_), 119.8 (2C, C_e_), 116.6 (2C, C_j_), 61.5 (1C, C_6_), 49.9 (2C, C_7_), 22.7 (2C, C_8_), 22.6 (2C, C_8′_). HRMS (ES^+^): calcd. for C_35_H_36_N_6_Ir 733.2631; found 733.2632 [M − PF_6_^−^]^+^.

*Complex (***4b**). A mixture of **3b** (43.3 mg, 0.10 mmol), silver oxide (23.8 mg, 0.10 mmol) and [IrCl(ppy)_2_]_2_ (50 mg, 0.05 mmol) in 10 mL of MeCN was refluxed for 10 h. After cooling down to room temperature, an aqueous solution of NH_4_PF_6_ (200 mg in 10 mL deionized water) was slowly added into the reaction mixture under stirring, resulting in a yellow suspension. The suspension was then filtered and the resulting precipitate was washed with deionized water and dried under vacuum at 70 °C for 12 h. The crude product was purified by column chromatography on silica gel with CH_2_Cl_2_/MeOH (30:1) as the eluent, yielding a yellow solid (40 mg, 47% yield). Anal. Calcd. for C_37_H_40_F_6_IrN_6_P: C, 49.05; H, 4.45; N, 9.28. Found: C, 49.50; H, 4.14; N, 9.33. ^1^H NMR (400 MHz, CDCl_3_): *δ* 8.13 (d, 2H, *J* = 6.0 Hz, H_b_), 7.95 (d, 2H, *J* = 8.0 Hz, H_e_), 7.81 (dd, 2H, *J* = 7.2, 7.2 Hz, H_d_), 7.65–7.58 (m, 4H, H_k_/H_5_), 7.05 (dd, 2H, *J* = 7.2, 7.2 Hz, H_c_), 6.89 (dd, 2H, *J* = 7.2, 7.2 Hz, H_j_), 6.84 (s, 2H, H_4_), 6.78 (dd, 2H, *J* = 7.2, 7.2 Hz, H_i_), 6.24 (d, 2H, *J* = 8.0 Hz, H_h_), 6.20 (s, 2H, H_6_), 3.31 (dd, 2H, *J* = 8.0, 5.2 Hz, H_7_), 3.07 (dd, 2H, *J* = 8.0, 5.2 Hz, H_7′_), 1.25–1.20 (m, 2H, H_8_), 0.47 (d, 6H, *J* = 6.4 Hz, H_9_), 0.12 (d, 6H, *J* = 6.4 Hz, H_9′_). ^13^C NMR (100 MHz, CDCl_3_): *δ* 169.7 (2C, C_f_), 163.6 (2C, C_2_), 162.8 (2C, C_l_), 152.7 (2C, C_b_), 143.9 (2C, C_g_), 136.7 (2C, C_d_), 131.4 (2C, C_h_), 129.7 (2C, C_i_), 124.6 (2C, C_5_), 123.5 (2C, C_k_), 122.2 (2C, C_c_), 121.5 (2C, C_4_), 120.4 (2C, C_e_), 119.8 (2C, C_j_), 61.5 (1C, C_6_), 54.9 (2C, C_7_), 28.3 (2C, C_8_), 19.1 (2C, C_9_), 18.5 (2C, C_9′_). HRMS (ES^+^): calcd. for C_37_H_40_N_6_Ir 761.2944; found 761.2936 [M − PF_6_^−^]^+^.

*Complex* (**4c**). A mixture of **3c** (41.2 mg, 0.10 mmol), silver oxide (23.8 mg, 0.10 mmol) and [IrCl(ppy)_2_]_2_ (50 mg, 0.05 mmol) in 10 mL of MeCN was refluxed for 10 h. After cooling down to room temperature, an aqueous solution of NH_4_PF_6_ (200 mg in 10 mL deionized water) was slowly added into the reaction mixture under stirring, resulting in a yellow suspension. The suspension was then filtered and the resulting precipitate was washed with deionized water and dried under vacuum at 70 °C for 12 h. The crude product was purified by column chromatography on silica gel with CH_2_Cl_2_/MeOH (20:1) as the eluent, yielding a yellow solid (55 mg, 60% yield). Anal. Calcd. for C_43_H_36_F_6_IrN_6_P: C, 53.03; H, 3.73; N, 8.63. Found: C, 53.01; H, 3.49; N, 8.15. ^1^H NMR (400 MHz, CDCl_3_): *δ* 8.25 (d, 2H, *J* = 5.6 Hz, H_b_), 7.68 (d, 2H, *J* = 7.6 Hz, H_e_), 7.64–7.58 (m, 4H, H_5_/H_d_), 7.52 (d, 2H, *J* = 7.6 Hz, H_k_), 7.15–7.04 (m, 6H, H_bn_), 6.98 (ddd, 2H, *J* = 6.0, 6.0, 1.6 Hz, H_c_), 6.79 (ddd, 2H, *J* = 7.2, 7.2, 1.2 Hz, H_j_), 6.71 (d, 2H, *J* = 2.0 Hz, H_4_), 6.67 (ddd, 2H, *J* = 7.2, 7.2, 1.2 Hz, H_i_), 6.34 (d, 4H, *J* = 7.2 Hz, H_bn_), 6.30 (s, 2H, H_6_), 6.23 (d, 2H, *J* = 7.6 Hz, H_h_), 4.81 (d, 2H, *J* = 15.6 Hz, H_7_), 4.49 (d, 2H, *J* = 15.6 Hz, H_7′_). ^13^C NMR (100 MHz, CDCl_3_): *δ* 169.3 (2C, C_f_), 164.3 (2C, C_2_), 161.8 (2C, C_l_), 152.4 (2C, C_b_), 143.9 (2C, C_g_), 136.6 (2C, C_d_), 135.9 (2C, C_bn_), 131.2 (2C, C_h_), 129.6 (2C, C_i_), 128.3 (4C, C_bn_), 127.3 (2C, C_bn_), 126.2 (4C, C_bn_), 124.5 (2C, C_5_), 124.0 (2C, C_k_), 122.2 (2C, C_c_), 121.6 (2C, C_4_), 121.4 (2C, C_e_), 119.8 (2C, C_j_), 61.7 (1C, C_6_), 52.2 (2C, C_7_). HRMS (ES^+^): calcd. for C_43_H_36_N_6_Ir 829.2631; found 829.2632 [M − PF_6_^−^].

*Complex* (**4d**). A mixture of **3d** (56.9 mg, 0.10 mmol), silver oxide (23.8 mg, 0.10 mmol) and [IrCl(ppy)_2_]_2_ (50 mg, 0.05 mmol) in 10 mL of MeCN was refluxed for 10 h. After cooling down to room temperature, an aqueous solution of NH_4_PF_6_ (200 mg in 10 mL deionized water) was slowly added into the reaction mixture under stirring, resulting in a yellow suspension. The suspension was then filtered and the resulting precipitate was washed with deionized water and dried under vacuum at 70 °C for 12 h. The crude product was purified by column chromatography on silica gel with CH_2_Cl_2_/MeOH (20:1) as the eluent, yielding a yellow solid (35 mg, 36% yield). Anal. Calcd. for C_43_H_36_F_6_IrN_6_PS_2_: C, 49.75; H, 3.50; N, 8.10. Found: C, 50.05; H, 3.42; N, 7.86. ^1^H NMR (400 MHz, CD_3_CN): *δ* 8.54 (d, 2H, *J* = 5.2 Hz, H_b_), 7.82 (ddd, 2H, *J* = 7.2, 7.2, 1.6 Hz, H_d_), 7.72 (d, 2H, *J* = 7.6 Hz, H_e_), 7.51 (d, 2H, *J* = 2.0 Hz, H_5_), 7.23 (d, 2H, *J* = 7.6 Hz, H_k_), 7.12 (ddd, 2H, *J* = 6.4, 6.4, 1.6 Hz, H_c_), 6.96 (d, 2H, *J* = 2.0 Hz, H_4_), 6.59 (ddd, 2H, *J* = 7.2, 7.2, 1.2 Hz, H_j_), 6.54 (d, 4H, *J* = 8.8 Hz, H_Ph_), 6.34–6.24 (m, 8H, H_6_/H_i_/H_Ph_), 5.44 (d, 2H, *J* = 7.6 Hz, H_h_), 2.44 (s, 6H, H_SMe_). ^13^C NMR (100 MHz, CDCl_3_): *δ* 169.8 (2C, C_f_), 164.9 (2C, C_2_), 162.9 (2C, C_l_), 153.1 (2C, C_b_), 142.7 (2C, C_g_), 138.4 (2C, C_Ph_), 136.6 (2C, C_d_), 135.6 (2C, C_Ph_), 130.0 (2C, C_h_), 129.2 (2C, C_i_), 126.2 (4C, C_Ph_), 125.3 (4C, C_Ph_), 124.6 (2C, C_5_), 124.0 (2C, C_k_), 122.8 (2C, C_c_), 121.5 (2C, C_4_), 120.2 (2C, C_e_), 119.7 (2C, C_j_), 61.8 (1C, C_6_), 15.2 (2C, C_SMe_). HRMS (ES^+^): calcd. for C_43_H_36_N_6_S_2_Ir 893.2072; found 891.2051 [M − PF_6_^−^]^+^.

*Complex* (**4e**). Under a nitrogen atmosphere and protection of the light, a flask was charged with **3e** (52.3 mg, 0.10 mmol), [IrCl(ppy)_2_]_2_ (53.5 mg, 0.05 mmol) and Ag_2_O (24.5 mg, 0.10 mmol) in 10 mL of MeCN and the mixture was stirred under reflux for 24 h. After cooling to room temperature, NH_4_PF_6_ (200 mg) was added to the reaction mixture under stirring. The suspension was then filtered over a pad of celite, the resulting precipitate was washed with MeOH and the solvent was evaporated form the filtrate. The crude product was purified by two columns chromatography on silica gel with CH_2_Cl_2_/MeOH (30:1) and (200:1), respectively, as the eluent. The product was still not pure, so it was solubilized in CH_2_Cl_2_ and precipitated with Et_2_O. After drying under vacuum, a yellow solid was obtained (49 mg, 49% yield). Anal. Calcd. for C_44_H_38_F_6_IrN_6_P: C, 53.49; H, 3.88; N, 8.51. Found: C, 53.34; H, 3.32; N, 8.77. ^1^H NMR (500 MHz, CDCl_3_): *δ* 8.50 (d, *J* = 5.5 Hz, 2H, H_b_), 7.60–7.57 (m, 4H, H_d_/H_e_), 7.47 (d, *J* = 6.5 Hz, 2H, H_h_), 7.11 (d, *J* = 2.0 Hz, 2H, H_4_), 7.10 (d, *J* = 1.5 Hz, 2H, H_bn_), 7.05 (dd, *J* = 8.5, 6.5 Hz, 4H, H_bn_), 6.97 (ddd, *J* = 7.0, 7.0, 2.5 Hz, 2H, H_c_), 6.76 (ddd, *J* = 7.0, 7.0, 1.0 Hz, 2H, H_i_), 6.55 (d, *J* = 2.0 Hz, 2H, H_5_), 6.53 (dd, *J* = 7.0, 1.0 Hz, 2H, H_j_), 6.21 (d, *J* = 7.5 Hz, 4H, H_bn_), 6.13 (d, *J* = 6.5 Hz, 2H, H_k_), 4.80 (d, *J* = 16.0 Hz, 2H, H_7_), 4.61 (s, 4H, H_6_), 4.51 (d, *J* = 16.0 Hz, 2H, H_7_). ^13^C NMR (125 MHz, CDCl_3_): *δ* 168.8 (2C, C_f_), 164.5 (2C, C_2_), 160.7 (2C, C_l_), 154.9 (2C, C_b_), 143.8 (2C, C_g_), 136.8 (2C, C_bn_), 136.5 (2C, C_d_), 131.4 (2C, C_k_), 129.1 (2C, C_j_), 128.3 (4C, C_bn_), 127.2 (2C, C_bn_), 126.0 (4C, C_bn_), 125.6 (2C, C_5_), 125.1 (2C, C_4_), 124.6 (2C, C_h_), 122.2 (2C, C_c_), 121.3 (2C, C_i_), 119.8 (2C, C_e_), 53.7 (2C, C_7_), 51.3 (2C, C_6_). HRMS (ES^+^): calcd. for C_44_H_38_N_6_Ir m/z = 843.2783; found m/z = 843.2806 [M − PF_6_^−^]^+^.

*Complex* (**4f**). Under a nitrogen atmosphere, a flask was charged with **3f** (43.6 mg, 0.10 mmol), [IrCl(ppy)_2_]_2_ (50 mg, 0.05 mmol) and K_2_CO_3_ (13 mg, 0.10 mmol) in 10 mL of MeCN and stirred under reflux for 24 h. After cooling to room temperature, the solvent was evaporated and the solid was dissolved in CH_2_Cl_2_. The solution was washed with water and brine, then dried with Na_2_SO_4_. The solution was filtered and the solvent was evaporated to obtain a yellow solid. The solid was treated with a mixture of CH_2_Cl_2_/Et_2_O (3:20) to give a yellow solid (55 mg, 60% yield). Anal. Calcd. for C_38_H_42_F_6_IrN_6_P: C, 49.61; H, 4.60; N, 9.14 Found: C, 49.17; H, 4.27; N, 9.33. ^1^H NMR (400 MHz, CDCl_3_): *δ* 8.43 (d, *J* = 5.6 Hz, 2H, H_b_), 7.91 (d, *J* = 8.0 Hz, 2H, H_e_), 7.81 (ddd, *J* = 7.2, 7.2, 1.6 Hz, 2H, H_d_), 7.57 (d, *J* = 8.0 Hz, 2H, H_h_), 7.15 (d, *J* = 2.0 Hz, 2H, H_4_), 7.06 (ddd, *J* = 7.2, 7.2, 1.6 Hz, 2H, H_c_), 6.83 (ddd, *J* = 7.2, 7.2, 1.2 Hz, 2H, H_i_), 6.77 (d, *J* = 2.0 Hz, 2H, H_5_), 6.69 (ddd, *J* = 7.2, 7.2, 1.2 Hz, 2H, H_j_), 6.21 (d, *J* = 8.0 Hz, 2H, H_k_), 4.58–4.47 (m, 4H, H_6_), 3.21 (dd, *J* = 13.4, 8.4 Hz, 2H, H_7_), 2.87 (dd, *J* = 13.4, 8.4 Hz, 2H, H_7′_), 1.58–1.53 (m, 2H, H_8_), 0.49 (d, *J* = 6.4 Hz, 6H, H_9_), 0.17 (d, *J* = 6.4 Hz, 6H, H_9′_). ^13^C NMR (100 MHz, CDCl_3_): *δ* 169.4 (2C, C_f_), 163.7 (2C, C_2_), 161.3 (2C, C_l_), 154.9 (2C, C_b_), 143.9 (2C, C_g_), 136.7 (2C, C_d_), 134.0 (2C, C_k_), 128.9 (2C, C_j_), 124.7 (2C, C_h_), 124.6 (2C, C_4_), 122.2 (2C, C_c_), 121.3 (2C, C_i_), 120.8 (2C, C_5_), 119.7 (2C, C_e_), 55.8 (2C, C_7_), 50.6 (2C, C_6_), 28.3 (2C, C_8_), 18.7 (2C, C_9_), 18.4 (2C, C_9′_). HRMS (ES^+^): calcd. for C_38_H_42_N_6_Ir 775.3100; found 775.3102 [M − PF_6_^−^]^+^.

*Complex* (**4g**). Under a nitrogen atmosphere and protection of the light, a flask was charged with **3g** (51.5mg, 0.15 mmol), [IrCl(ppy)_2_]_2_ (70,8 mg, 0.07 mmol) and Ag_2_O (18.8 mg, 0.08 mmol) in 10 mL of MeCN and the resulting mixture was stirred under reflux for 24 h. After cooling to room temperature, the mixture was filtered over a pad of celite and the solvent was evaporated from the filtrate. The product was dissolved in CH_2_Cl_2_, precipitated by addition of Et_2_O, filtered and dried under vacuum, yielding a yellow solid (65 mg, 51% yield). Anal. Calcd. for C_43_H_35_IrN_5_Cl: C, 60.80; H, 4.15; N, 8.24. Found: C, 60.54; H, 4.22; N, 8.42. ^1^H NMR (400 MHz, CDCl_3_): *δ* 10.00 (s, 1H, H_5_), 9.55 (*J* = 8.4 Hz, 1H, H_7_), 8.72 (d, *J* = 8.4 Hz, 1H, H_8_), 8.09 (d, *J* = 8.4 Hz, 1H, H_13_), 8.05 (d, *J* = 5.6 Hz, 1H, H_b_), 7.87 (d, *J* = 5.6 Hz, 1H, H_n_), 7.84 (dd, *J* = 8.4, 1.6 Hz, 1H, H_10_), 7.81–7.75 (m, 2H, H_e_/H_d_), 7.72 (d, *J* = 8.4 Hz, 1H, H_q_), 7.65 (dd, *J* = 8.0, 8.0 Hz, 1H, H_p_), 7.55–7.51 (m, 1H, H_x_), 7.41 (ddd, *J* = 8.0, 8.0, 1.2 Hz, 1H, H_11_), 7.14 (ddd, *J* = 8.0, 6.8, 1.2 Hz, 1H, H_12_), 7.09 (dd, *J* = 8.0, 1.2 Hz, 1H, H_h_), 6.98 (s, 1H, H_4_), 6.97–6.90 (m, 4H, H_c_/H_v_/H_w_/H_o_), 6.62 (ddd, *J* = 8.0, 8.0, 1.2 Hz, 1H, H_i_), 6.46–6.40 (m, 2H, H_j_/H_22_), 6.27–6.23 (m, 2H, H_u_/H_19_), 5.90 (dd, *J* = 8.0, 1.2 Hz, 1H, H_k_), 1.95 (s, 6H, H_18_/H_24_), 0.93 (s, 3H, H_21_). ^13^C NMR (100 MHz, CDCl_3_): *δ* 180.4 (1C, C_2_), 169.3 (1C, C_r_), 167.9 (1C, C_f_), 165.6 (1C, C_t_), 154.2 (1C, C_6_), 151.8 (1C, C_n_), 150.7 (1C, C_b_), 146.3 (1C, C_l_), 145.2 (1C, C_14_), 144.0 (1C, C_8_), 143.6 (1C, C_s_), 141.4 (1C, C_g_), 139.0 (1C, C_16_), 137.7 (1C, C_p_), 136.8 (1C, C_d_), 133.9 (1C, C_23_), 133.7 (1C, C_12_), 133.6 (1C, C_19_), 131.5 (1C, C_v_), 130.8 (1C, C_j_), 130.6 (1C, C_20_), 129.5 (1C, C_17_), 129.5 (1C, C_u_), 129.0 (1C, C_k_), 128.7 (1C, C_10_), 128.0 (1C, C_22_), 127.6 (1C, C_9_), 127.6 (1C, C_13_), 127.1 (1C, C_11_), 125.5 (1C, C_4_), 124.7 (1C, C_x_), 123.7 (1C, C_h_), 123.0 (1C, C_o_), 122.5 (1C, C_w_), 122.5 (1C, C_c_), 122.4 (1C, C_5_), 120.2 (1C, C_i_), 119.6 (1C, C_q_), 119.5 (1C, C_e_), 113.9 (1C, C_7_), 20.9 (1C, C_21_), 18.7 (1C, C_24_), 15.9 (1C, C_18_). HRMS (ES^+^): calcd. for C_43_H_35_N_5_Ir 814.2522; found 814.2520 [M − Cl^−^]^+^.

#### 3.1.4. Crystallographic Data for **4a**, **4b**, **4c**, **4e** and **4f**

All data were collected at low temperature using oil-coated shock-cooled crystals at 100(2) K on a Bruker-AXS APEX II diffractometer with MoKa radiation (λ = 0.71073 Å). The structures were solved by direct methods [85] and all non-hydrogen atoms were refined anisotropically using the least-squares method on *F^2^* [86]. The absolute structure parameters have been refined using the Flack method [87]. CCDC 2224756-2224760 contain the supplementary crystallographic data for this paper. These data can be obtained free of charge via http://www.ccdc.cam.ac.uk/conts/retrieving.html (accessed on 06~December 2022) (or from the CCDC, 12 Union Road, Cambridge CB2 1EZ, UK; Fax: +44 1223 336033; e-mail: deposit@ccdc.cam.ac.uk).

**4a**: C_35_H_36_F_6_IrN_6_P, Mr = 877.87, crystal size = 0.20 × 0.10 × 0.10 mm^3^, orthorhombic, space group *P2_1_2_1_2_1_*, *a* = 10.5858(2) Å, *b* = 17.6393(3) Å, *c* = 17.8473(3) Å, V = 3332.6(1) Å^3^, Z = 4, 25,742 reflections collected, 15,373 unique reflections (R_int_ = 0.0285), R1 = 0.0263, wR2 = 0.0553 [I>2σ(I)], R1 = 0.0303, wR2 = 0.0568 (all data), Flack x refined to –0.021(3), residual electron density = 1.720 e Å^−3^.

**4b**: C_37_H_41_Br_0.25_Cl_0.75_IrN_6_O, Mr = 824.52, crystal size = 0.20 x 0.20 x 0.10 mm^3^, monoclinic, space group *C2/c*, *a* = 39.743(2) Å, *b* = 8.954(1) Å, *c* = 22.453(2) Å, *β* = 121.284(2)°, V = 6827.9(6) Å^3^, Z = 8, 51,077 reflections collected, 8390 unique reflections (R_int_ = 0.0465), R1 = 0.0290, wR2 = 0.0524 [I>2σ(I)], R1 = 0.0421, wR2 = 0.0554 (all data), residual electron density = 0.961 e Å^−3^.

**4c**: C_43.5_H_37_ClF_6_IrN_6_O, Mr = 1016.41, crystal size = 0.40 x 0.30 x 0.20 mm^3^, monoclinic, space group *P2_1_*, *a* = 11.03(2) Å, *b* = 24.53(4) Å, *c* = 14.32(2) Å, *β* = 92.13(2)°, V = 3873(10) Å^3^, Z = 4, 64,391 reflections collected, 16,738 unique reflections (R_int_ = 0.0314), R1 = 0.0325, wR2 = 0.0694 [I>2σ(I)], R1 = 0.0378, wR2 = 0.0750 (all data), Flack x refined to 0.482(6), residual electron density = 1.360 e Å^−3^.

**4e**: C_47_H_45.5_F_6_IrN_6_O_0.75_P, Mr = 1043.56, crystal size 0.2 x 0.1 x 0.02 mm^3^, triclinic, space group *P*$\bar{1}$, *a* = 14.94(2) Å, *b* = 15.06(2) Å, *c* = 20.52(3) Å, *α* = 107.64(2)°, *β* = 107.73(2)°, *γ* = 94.99(3)°, V = 4108(10) Å^3^, Z = 4, 53,646 reflections collected, 15,235 unique, R_int_ = 0.0737, R1 = 0.0522, wR2 = 0.0980 [I > 2σ(I)], R1 = 0.1021, wR2 = 0.1156 (all data), residual electron density = 1.562 eA^−3^.

**4f**: C_38_H_42_F_6_IrN_6_P, Mr = 919.94, crystal size 0.4 x 0.2 x 0.2 mm^3^, orthorhombic, *P2_1_2_1_2_1_*, *a* = 10.806(1) Å, *b* = 17.084(2) Å, *c* = 19.875(2) Å, V = 3669.1(4) Å^3^, Z = 4, 54,609 reflections collected, 11,153 unique, R_int_ = 0.0326, R1 = 0.0142, wR2 = 0.0322 [I > 2σ(I)], R1 = 0.0162, wR2 = 0.0328 (all data), Flack x refined to 0.497(3), residual electron density = 0.635 eA^−3^.

#### 3.1.5. Measurement of Lipophilicity

The octanol–water partition coefficients (P) of **4a** to **4g** were determined using a shake-flask method [70]. Water (100 mL, distilled after milli-Q purification) and n-octanol (100 mL, Sigma-Aldrich, St. Louis, MO, USA, ACS spectrophotometric grade, ≥99%) were shaken together for 24 h to allow saturation of both phases. Stock solutions of the four compounds (50 μM) were prepared in the aqueous phase and aliquots (1 mL) of each of these stock solutions were then added to an equal volume of the n-octanol phase. The resultant biphasic solutions were mixed for 1 h and then centrifuged (3000× *g*, 5 min) (Eppendorf Centrifuges 5415R and 5804R) to separate the phases. The concentrations of the complexes in the organic and aqueous phases were then determined using UV absorbance spectroscopy (260 nm). Log P was defined as the logarithm of the ratio of the concentrations of the studied complex in the organic and aqueous phases (Log P = Log {[Ir(org)]/[Ir(aq)]}; values reported are the means of at least two separate determinations).

#### 3.1.6. Photophysical Measurements

UV/Vis absorption spectra were recorded at RT on solutions contained in quartz cuvettes (optical pathlength 1 cm, Hellma^®^ (HELLMA, Müllheim, Germany)) with a PerkinElmer^®^ (PerkinElmer, Waltham, MA, USA) UV/VIS/NIR spectrophotometer Lambda 950 and a PerkinElmer^®^ detector UV/VIS/NIR Accessory 2D Detector Module.

Fluorescence and phosphorescence emission spectra were obtained with a HORIBA JOBIN YVON (Bensheim, Germany) fluromax-4 and spectrofluorometer.

Luminescence quantum yields were measured according to the method of Demas and Crosby [88], on solution samples at RT. The quantum yield is defined as:

$$\Phi_{S}=\Phi_{R}*\frac{\left(A_{s}\right)}{\left(A_{r}\right)}*\;{\frac{\left(\eta_{s}\right)}{{\left(\eta_{r}\right)}^{2}}}^{2}$$

where $\Phi_{S}\;$ is the quantum yield of the sample, $\Phi_{R}$ is the known quantum yield of the reference standard at the area subtended by the emission band (on a wavelength scale) and η is the refractive index of the solvent used for the preparation of the solution. As (related to the sample) and Ar (related to the reference) must be excitation wavelength. Different standards were selected depending on the spectral region of the interest: POPOP in hexane (Φ = 97%) and QUININNE in water (Φ = 54%).

Fluorescence lifetime measurements were performed by an Edinburgh FLS920 spectrofluorometer equipped with a TCC900 card for data acquisition in time-correlated single-photon counting experiments (0.5 ns time resolution) with a D2 lamp and an LDH-P-C-405 pulsed diode laser. The estimated experimental error was 2 nm on the band maximum (5% on the lifetime, 10% on the fluorescence quantum yield).

### 3.2. Biology

#### 3.2.1. Cell Culture and Reagents 

Unless otherwise indicated, cell lines were obtained from DSMZ (Braunschweig, Germany) or ATCC (Molsheim, France). Culture medium, antibiotics and fetal bovine serum were from Fisher Scientific (Illkirch, France). Non-cancerous murine NIH-3T3 fibroblasts and murine MC3T3 osteoblasts were maintained in Dulbecco’s modified eagle medium (DMEM) and MEMα formulated with the addition of 10% FBS, respectively. Human T24 bladder, HeLa cervical and A549 lung cancer cells were maintained in DMEM, supplemented with 10% FBS. PC-3 human prostate cancer cells were maintained in RPMI1640, supplemented with 10% FBS. MCF7 cells stably transfected to express Fas antigen (designated as MCF7/FasR) or Fas and Bcl-xL (designated as MCF7/FasR/Bcl-xL) were donated by Dr. Vishva Dixit (Genentech Inc., San Francisco, CA, USA) and were grown in RPMI1640 containing 10% fetal bovine serum supplemented with 200 µg/mL G418 and 150 µg/mL hygromycin, as previously reported [76]. Cell lines were systematically checked by the following tests: morphological examination, growth analysis and mycoplasma detection (MycoAlert^TM^, Lonza, Basel, Switzerland). All experiments were started with cells with low passages (<25 times).

TNF-α was from Bio-techne (Minneapolis, MN, USA), activating anti-Fas antibody (clone CH-11) was from Sigma-Aldrich and z-VAD-fmk was from Bachem (Bubendorf, Switzerland).

#### 3.2.2. Treatments and Conditions of Irradiation

For all biology experiments, the compounds were solubilized in DMSO to reach a final fixed concentration equal to 0.1% *v/v* to avoid cytotoxicity caused by DMSO [89]. The results are normalized to controls (0.1% *v/v* DMSO). Depending on the cell type, approximatively 1000 to 10,000 cells/cm^2^ were seeded in 48-well plates for MTT, ATP and GSH assays and 60 mm Petri dishes for Western blots. Cells were allowed to attach overnight and then incubated for 4 h with the different complexes (in the dark), then irradiated for 10 min, followed by an incubation in the dark up to 72 h. 

Irradiation was conducted using an in-house device: a series of blue light LEDs selected according to the absorption spectra of the compounds at a 458 nm wavelength with an emitted power of 9.6 watts. The absorbed energy, or irradiance, was measured at 458 nm with a Thorlabs Optical Power Meter PM100A at 5 and 10 cm distance between the cell layer and the light source and expressed as mW/cm^2^.

#### 3.2.3. Cell Viability Assays

A tetrazolium-based MTT reagent (3-(4,5-dimethylthiazol-2-yl)-2,5-diphenyltetrazolium bromide) was used to determine cell viability, as previously described [90]. After 72 h of treatment at 37 °C and 5% CO_2_, cells were incubated with 25 μL MTT solution (5 mg/mL; Sigma-Aldrich) for 4 h. After solubilization with 500 μL of lysis buffer (DMSO), formazan was quantified by spectrophotometry with a microplate reader at 570 nm absorbance (ClarioSTAR, BMG LabTech, Champigny-sur-Marne, France). GI_50_ values corresponding to the concentration that caused 50% inhibition of cell proliferation were calculated from dose–response curves obtained by nonlinear regression analysis. All results were calculated from data obtained in at least three independent experiments.

#### 3.2.4. ATP and GSH Quantification

ATP and glutathione (GSH) contents were measured with the Promega CellTiter-Glo (Cat#G7570) and GSH-Glo (cat#6911) luminescent assays, respectively, according to the manufacturer’s recommendations.

#### 3.2.5. Preparation of Cellular Extracts and Western Blot Analysis

Cell lysate preparation and Western blotting were carried out as previously described [91]. Briefly, at the end of the experiment, the cells were washed with cold PBS and the pellets suspended in the following lysis buffer: RIPA lysis buffer system (Santa Cruz, cat#sc-24948), cOmplete^TM^ mini EDTA-free protease inhibitor cocktail (Sigma-Aldrich), anti-phosphatase (Phosphatase Inhibitor Cocktail Set II from Millipore). The samples were then sonicated for 20 sec. After centrifugation at 4 °C for 15 min, the supernatant was recovered and the proteins assayed by the Bradford method. Protein separation was performed on 10% or 15% polyacrylamide cross-linked SDS-PAGE gels, depending on the molecular weight of the protein of interest. Mouse anti-actin (Sigma-Aldrich, Cat#A2228), rabbit full-length anti-PARP (CST, Cat#9542), rabbit cleaved anti-PARP (Abcam, Cat#ab32064), mouse anti-caspase-7 (CST, Cat #9494), mouse anti-caspase-8 (CST, Cat#9746) and rabbit anti-caspase-3 (CST, Cat#4220) were used as primary antibodies. Proteins were visualized by ECL (Clarity Western ECL Substrate, Bio-Rad, Marnes-la-Coquette, France) using anti-rabbit or anti-mouse HRP-conjugated IgG (Bio-Rad); analysis of Western blots was assessed with a ChemiDoc imaging system (Bio-Rad).

#### 3.2.6. Mitochondrial Subcellular Localization

Cell imaging was performed on an LSM 710 NLO-Meta confocal microscope with spectral detection (Zeiss, Göttingen, Germany). Images were taken through a 40×/1.2 W objective. Microscopy imaging experiments were performed with PC-3 cells that were cultured in 6-well plates and on glass slides (180,000 cells per well) for 18 h before incubation with complex **4a** at different concentrations (0.25 μM, 0.5 μM and 1 μM) for various times. After 24 h, the MitoTracker deep red probe (Fisher Scientific, Illkirch, France) was incubated on live cells at a final concentration of 200 nM during 15 min at 37 °C in a 5% CO_2_ humidified incubator and then observed in one pass two channels 440–544 nm for complex **4a** (two-photon excitation at 720 nm) and 644–700 nm for MitoTracker (excitation at 633 nm) at the same time. Merged images were generated to evaluate colocalization of the mitochondrial probe with complex **4a**.

#### 3.2.7. Statistical Analysis

With the exception of experiments performed less than three times, for which no statistical analysis was performed, all reported *p* values were obtained from two-tailed tests at the 0.05 level of significance. All graphs represent the means, with the error bars representing SD. Statistical analysis was performed using GraphPad Prism 9.3 software (San Diego, CA, USA).

## 4. Conclusions

In conclusion, three families of cyclometalated iridium(III) complexes bearing different NHC ligands have been prepared and characterized. The first two families contain a bis-carbene ligand in which the two NHCs are bridged by a methylene (C1) and an ethylene (C2), giving rise to cationic complexes **4a** to **4f**. The other one (C0) contains only one mono-carbene ligand and one chloride, leading to neutral complex 4g. All the iridium complexes displayed high anti-proliferative activity against PC-3 and T24 cancer cells. Moreover, they exhibited selectivity between cancer cells (PC-3 and T24) and normal cells (NIH-3T3). The charge on the iridium complexes has no effect on their anti-proliferative activity and selectivity. Moreover, complexes **4a** to **4d** (C1 system) can act as efficient photosensitizers. The cytotoxicity of **4a** to **4d** against PC-3 and T24 was increased substantially upon irradiation at 458 nm.

Notably, complex **4a** can be rapidly and efficiently taken up into PC-3 cells and specifically localized into mitochondria. Different types of cell death have been reported with iridium(III) complexes, from classical apoptosis to non-apoptotic forms of cell death that could be related notably to paraptosis, although this modality of cell death has not yet been characterized by indisputable biochemical markers. 

Our data clearly suggest that complex **4a** does not require activation of both intrinsic and extrinsic pathways to induce cancer cell death but may rather rely on a mitochondrial-dependent necrotic cell death. A key feature of human cancers is intrinsic or acquired resistance to apoptosis, including upregulation of anti-apoptotic proteins (e.g., Bcl-2, Bcl-xL, IAPs) and pro-survival signaling pathways (e.g., PI3K/Akt, NF-kB) and/or downregulation of pro-apoptotic proteins (e.g., Bax, Bad, Bim). This bypass of apoptosis can contribute to carcinogenesis, cancer progression and resistance to treatment, since most current cancer therapies act primarily by activating apoptosis. By inducing apoptosis-independent cell death in cancer cells, the complex **4a** described here is among the potential therapeutic agents that might improve the efficacy of cancer therapeutics by overcoming apoptosis resistance though alternative types of cell death.

Our preliminary studies demonstrated that these iridium(III) complexes have high potential to be mitochondria-targeted theranostic and photodynamic anticancer agents, showing a non-conventional type of cell death.

## Figures and Tables

**Table 1:** Photophysical data of iridium complexes **4a**–**4g**.

Complex	λ_em_ (nm) ^a^	Φ_PL_(%) ^b^	τ_1_/ns
**4a**	469, 505	1.2	191
**4b**	470, 503	1.6	166
**4c**	469, 499	1.6	180
**4d**	471, 506	1.1	167
**4e**	480, 510	0.2	-
**4f**	485, 510	0.2	-
**4g**	425, 535	0.2	-

^a^ λ_em_ were measured in CH_2_Cl_2_ at 298 K, λ_ex_ = 360 nm. ^b^ The emission phosphorescence quantum yields (Φ_PL_) were determined at 298 K using POPOP in hexane (Φ_R_ = 97%) and quinine sulfate (Φ_R_ = 54%) as the references.

**Table 2:** Cytotoxicity and selectivity of complexes **4a** to **4d** on a panel of cancer cells (T24 bladder, PC-3 prostate, MCF7 breast, A549 lung and HeLa cervix) versus non-cancerous NIH-3T3 fibroblasts and MC3T3 osteoblasts (GI_50_ (µM), 72 h, MTT assay).

		4a	4b	4c	4d	Precursor
NON TUMORAL CELLS						
**NIH-3T3**	**Dark**	**3.67**	**1.95 ^[a]^**	**2.73**	**2.33**	**40**
Fibroblasts	**Blue**	**2.79**	**1.52 ^[a]^**	**2.67**	**2.78**	**14**
	*p* value	0.1117 (NS)	0.1658 (NS)	0.8524 (NS)	0.3882 (NS)	
	*PI ^[b]^*	*1.3*	*1.3*	*1.0*	*0.8*	*2.9*
**MC3T3**	**Dark**	**2.46**	**0.979**	**1.16**	**2.08**	
Osteoblasts	**Blue**	**1.74**	**0.704**	**1.33**	**1.80**	
	*p* value	0.1008 (NS)	0.2273 (NS)	0.4313 (NS)	0.1856 (NS)	
	*PI*	*1.3*	*1.3*	*1.0*	*0.8*	
**TUMORAL CELLS**						
**T24**	**Dark**	**0.288**	**0.259**	**0.358**	**0.501**	**26**
Bladder	*SI ^[c]^ vs NIH*	*12.7*	*7.5*	*7.6*	*4.7*	*1.5*
	*SI ^[c]^ vs MC3T3*	*8.5*	*3.8*	*3.2*	*4.2*	
	**Blue**	**0.082**	**0.090**	**0.091**	**0.165**	**22**
	*SI vs NIH*	*34.0*	*16.7*	*29.3*	*16.8*	*0.7*
	*SI vs MC3T3*	*21.2*	*7.7*	*14.6*	*10.9*	
	*p* value	0.0080 (**)	0.0027 (**)	0.0027 (**)	0.0007 (***)	
	*PI*	*3.5*	*2.8*	*3.9*	*3.0*	*1.2*
**PC-3**	**Dark**	**0.233**	**0.257**	**0.336**	**0.509**	**29**
Prostate	*SI vs NIH*	*15.8*	*7.6*	*8.1*	*4.6*	*1.4*
	*SI vs MC3T3*	*10.6*	*3.8*	*3.5*	*4.1*	
	**Blue**	**0.057**	**0.071**	**0.116**	**0.157**	**14**
	*SI vs NIH*	*48.9*	*21.4*	*23.0*	*17.7*	*1.0*
	*SI vs MC3T3*	*30.5*	*9.9*	*11.5*	*11.5*	
	*p* value	<0.0001 (***)	<0.0001 (***)	0.0001 (***)	<0.0001 (***)	
	*PI*	*4.1*	*3.6*	*2.9*	*3.2*	*2.1*
**MCF7**	**Dark**	**0.213**	**0.211**	**0.248**	**0.365**	
Breast	*SI vs NIH*	*17.2*	*9.2*	*11.0*	*6.4*	
	*SI vs MC3T3*	*11.5*	*4.6*	*4.7*	*5.7*	
	**Blue**	**0.050**	**0.053**	**0.061**	**0.117**	
	*SI vs NIH*	*55.8*	*28.7*	*43.8*	*23.8*	
	*SI vs MC3T3*	*34.8*	*13.3*	*21.8*	*15.4*	
	*p* value	0.0003 (***)	0.0003 (***)	0.0029 (**)	<0.0001 (***)	
	*PI*	*4.3*	*4.0*	*4.1*	*3.1*	
**A549**	**Dark**	**0.355**	**0.354**	**0.407**	**1.51**	
Lung	*SI vs NIH*	*10.3*	*5.5*	*6.7*	*1.5*	
	*SI vs MC3T3*	*6.9*	*2.8*	*2.9*	*1.4*	
	**Blue**	**0.107**	**0.084**	**0.171**	**0.617**	
	*SI vs NIH*	*26.1*	*18.1*	*15.6*	*4.5*	
	*SI vs MC3T3*	*16.3*	*8.4*	*7.8*	*2.9*	
	*p* value	0.0039 (**)	0.0043 (**)	0.0005 (***)	<0.0001 (***)	
	*PI*	*3.3*	*4.2*	*2.4*	*2.4*	
**HeLa**	**Dark**	**1.29**	**0.625**	**0.494**	**1.54**	
Cervix	*SI vs NIH*	*2.8*	*3.1*	*5.5*	*1.5*	
	*SI vs MC3T3*	*1.9*	*1.6*	*2.3*	*1.4*	
	**Blue**	**0.525**	**0.294**	**0.275**	**0.817**	
	*SI vs NIH*	*5.3*	*5.2*	*9.7*	*3.4*	
	*SI vs MC3T3*	*3.3*	*2.4*	*4.8*	*2.2*	
	*p* value	0.0029 (**)	0.0009 (***)	0.0038(**)	0.0048 (***)	
	*PI*	*2.5*	*2.1*	*1.8*	*1.9*	
**AVERAGE SI ^[d]^**						
**Blue light**	*SI vs NIH*	*34.0*	*18.0*	*24.3*	*13.2*	
	*SI vs MC3T3*	*21.2*	*8.3*	*12.1*	*8.6*	

^[a]^ GI_50_ values represent the concentration of the complex causing 50% inhibition of cell growth. Results are means ± SD of at least 3 independent experiments. *p* value calculated between GI_50_ from non-irradiated and irradiated cells (***, *p* < 0.0001, **, *p* < 0.005, NS—not significant). ^[b]^ Photosensitizing index (PI = GI_50_ of non-irradiated cells/GI_50_ of irradiated cells). ^[c]^ Selectivity index (SI = GI_50_ of NIH-3T3 or MC3T3 cells/GI_50_ of a given cancer cell line). ^[d]^ Average selectivity index = GI_50_ of NIH-3T3 or MC3T3 cells/GI_50_ of all irradiated cancer cell lines.

## Data Availability

The structure parameters of obtained compounds were deposited with the Cambridge Structural Database. CCDC 2224756-2224760 contain the supplementary crystallographic data for this paper. These data can be obtained free of charge via http://www.ccdc.cam.ac.uk/conts/retrieving.html (accessed on 06 December 2022) (or from the CCDC, 12 Union Road, Cambridge CB2 1EZ, UK; Fax: +44-1223-336033; E-mail: deposit@ccdc.cam.ac.uk).

## Supplementary Materials

The following supporting information can be downloaded at: https://www.mdpi.com/article/10.3390/molecules28020691/s1, Table S1. Cytotoxic activity of iridium(III) NHC complexes **4a**–**4g** and NMR spectra of proligands **3a**–**3f** and iridium(III) NHC complexes **4a**–**4g**. References [92,93] are cited in the Supplementary Materials.

## Sample Availability

Samples of the compounds are available from the authors.
