# Supplementary material for: N-Heterocyclic Carbene-Iridium Complexes as Photosensitizers for In Vitro Photodynamic Therapy to Trigger Non-Apoptotic Cell Death in Cancer Cells
Source: Molecules. 2023 Jan 10;28(2):691. doi: 10.3390/molecules28020691 (PMC9861386; doi:10.3390/molecules28020691)
Supplement: Supplementary file 1 [file molecules-28-00691-s001.zip › molecules-2132297-supplementary-final.pdf]

Supplementary Materials

N-Heterocyclic Carbene-Iridium Complexes as Photosensitizers for In Vitro Photodynamic Therapy to Trigger Non-Apoptotic Cell Death in Cancer Cells

Xing Wang [1,†], Chen Zhang [1,2,†], Ryma Madji [1,2,†], Camille Voros [1], Serge Mazères [2], Christian Bijani [1], Céline Deraeve [1], Olivier Cuvillier [1,2,*], Heinz Gornitzka [1,*], Marie-Lise Maddelein [1,2,*] and Catherine Hemmert [1,*]

Table S1. Cytotoxic activity of iridium(III) NHC complexes **4a−4g**…….………..…………S1

^{1}H and ^{13}C NMR spectra of proligands **3a−3f** ……….……………………….……….S2−S8

^{1}H and ^{13}C NMR spectra of iridium(III) NHC complexes **4a−4g**......................................S9−S15

2D NMR spectra of iridium(III) NHC complex **4a**……………………………………S16−S17

Table S1. Cytotoxic activity of iridium(III) NHC complexes **4a**–**4g** expressed as GI_{50} (µM) values towards selected cancer and normal cell lines as determined by MTT assay (48 h).[a]

Complex	PC-3	T24	NIH3T3	SI (PC-3)	SI (T24)
**4a**	0.48	0.31	3.3	6.9	10.6
**4b**	0.51	0.38	3.5	6.9	9.2
**4c**	0.41	0.43	7.1	17.3	16.5
**4d**	0.71	0.95	8.5	12.0	8.9
**4e**	0.25	0.29	2.5	10.0	8.6
**4f**	0.91	0.91	4.1	4.5	4.5
**4g**	0.27	0.25	2.7	10.0	10.8
$[IrCl(ppy)_2]_2$	>20	14.8	>20	>1	>1.4

[a] GI_{50} values correspond to the concentration of complexes causing 50 % inhibition of cell growth. Data were obtained from three independent experiments.

The cytotoxic effects of the NHC precursors, the imidazolium salts, which represents the potential hydrolysis and/or degradation products of the complexes, have been published before [SI-1, SI-2].

SI-1 Hemmert, C.; Fabié, A.; Fabre, A.; Benoit-Vical, F.; Gornitzka, H. Synthesis, structures, and antimalarial activities of some silver(I), gold(I) and gold(III) complexes involving N-heterocyclic carbene ligands. *Eur. J. Med. Chem*. **2013**, *60*, 64–75. https://doi.org/10.1016/j.ejmech.2012.11.038.

SI-2 Hemmert, C.; Ramadani, A.P.; Boselli, L.; Álvarez, Á.F.; Paloque, L.; Augereau, J.-M.; Gornitzka, H.; Benoit-Vical, F. An-tiplasmodial activities of gold(I) complexes involving functionalized N-heterocyclic carbenes. *Bioorg. Med. Chem*. **2016**, *24*, 3075–3082. https://doi.org/10.1016/j.bmc.2016.05.023.

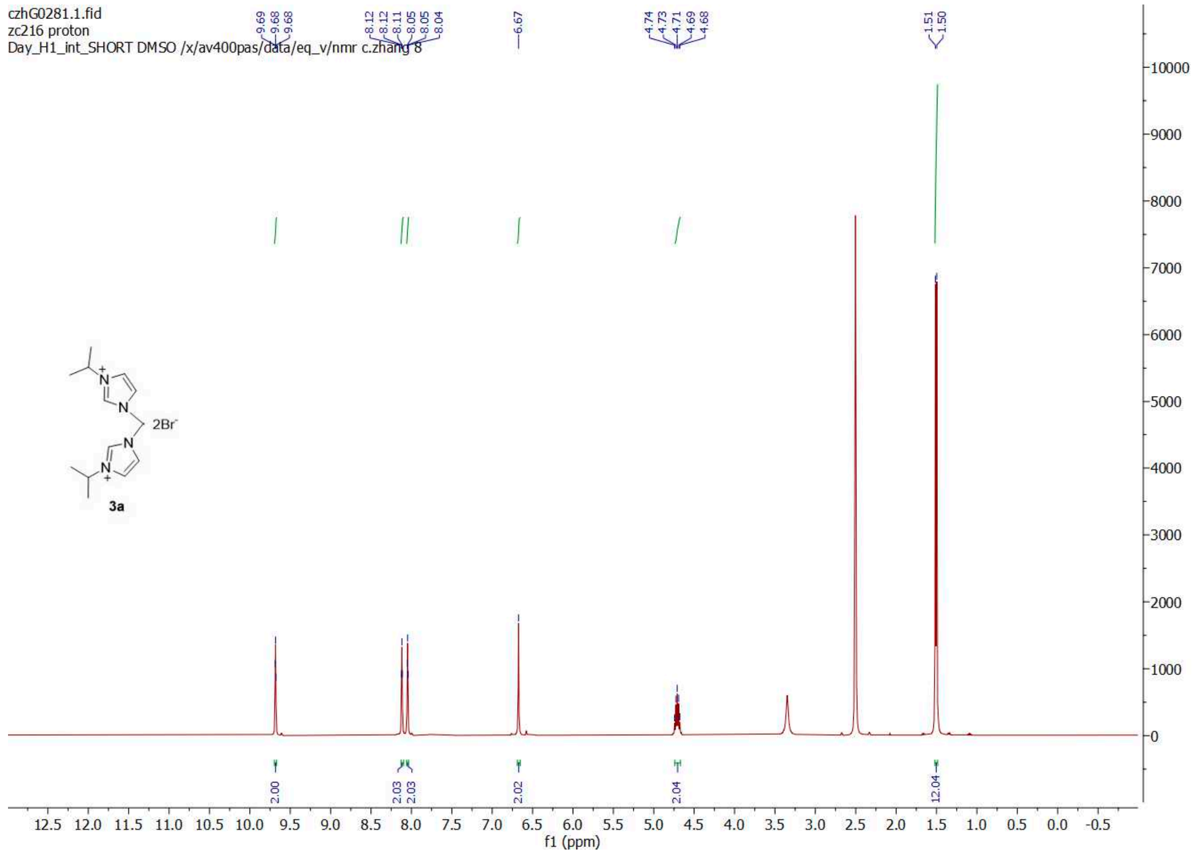
czhG0281.1.fid
zc216 proton
Day_H1_int_SHORT DMSO /x/av400pas/data/eq_v/nmr c.zhang 8
9.69
9.68
9.68
8.12
8.12
8.11
8.05
8.05
8.04
6.67
4.74
4.73
4.71
4.69
4.68
1.51
1.50
2Br⁻
3a
2.00
2.03
2.03
2.02
2.04
12.04
f1 (ppm)

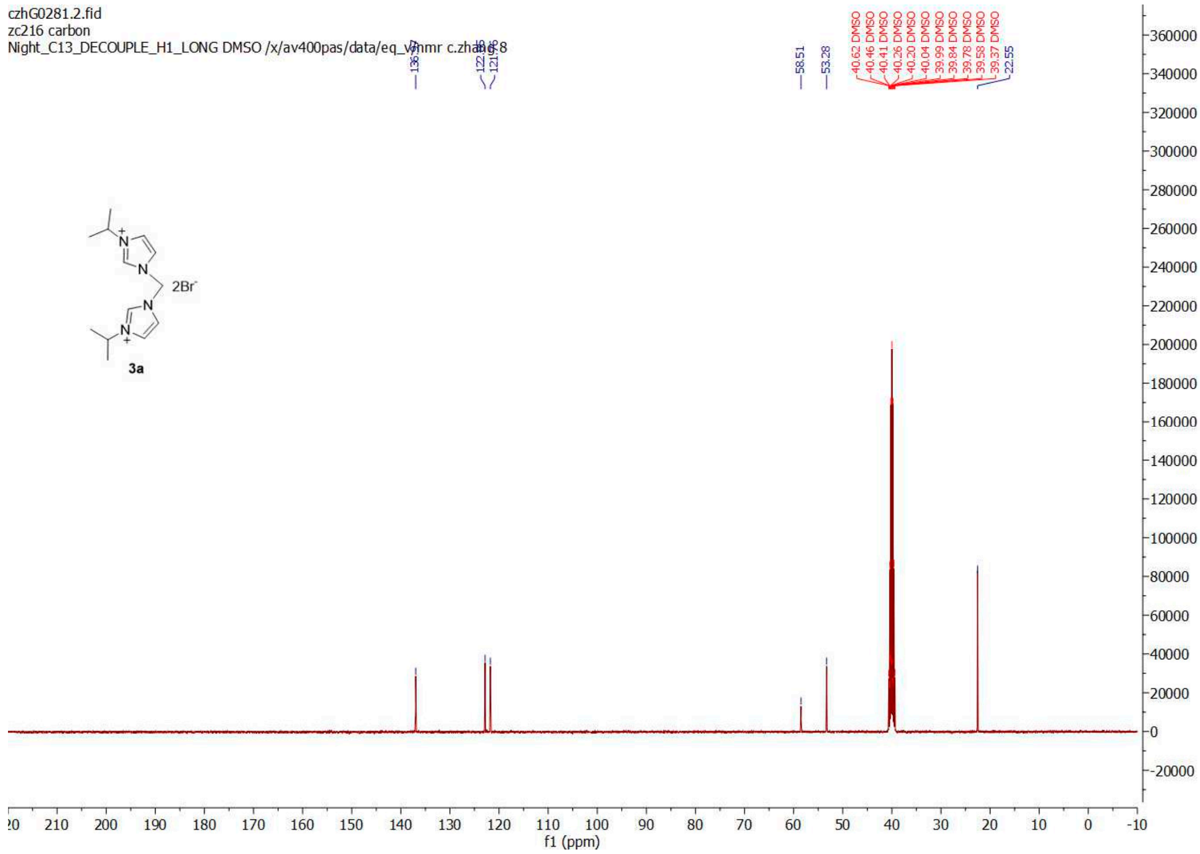
czhG0281.2.fid
zc216 carbon
Night_C13_DECOUPLE_H1_LONG DMSO /x/av400pas/data/eq_v/nmr c.zhang 8
58.51
53.28
40.62 DMSO
40.46 DMSO
40.41 DMSO
40.26 DMSO
40.20 DMSO
40.04 DMSO
39.99 DMSO
39.84 DMSO
39.78 DMSO
39.58 DMSO
39.37 DMSO
22.55
2Br⁻
3a
f1 (ppm)

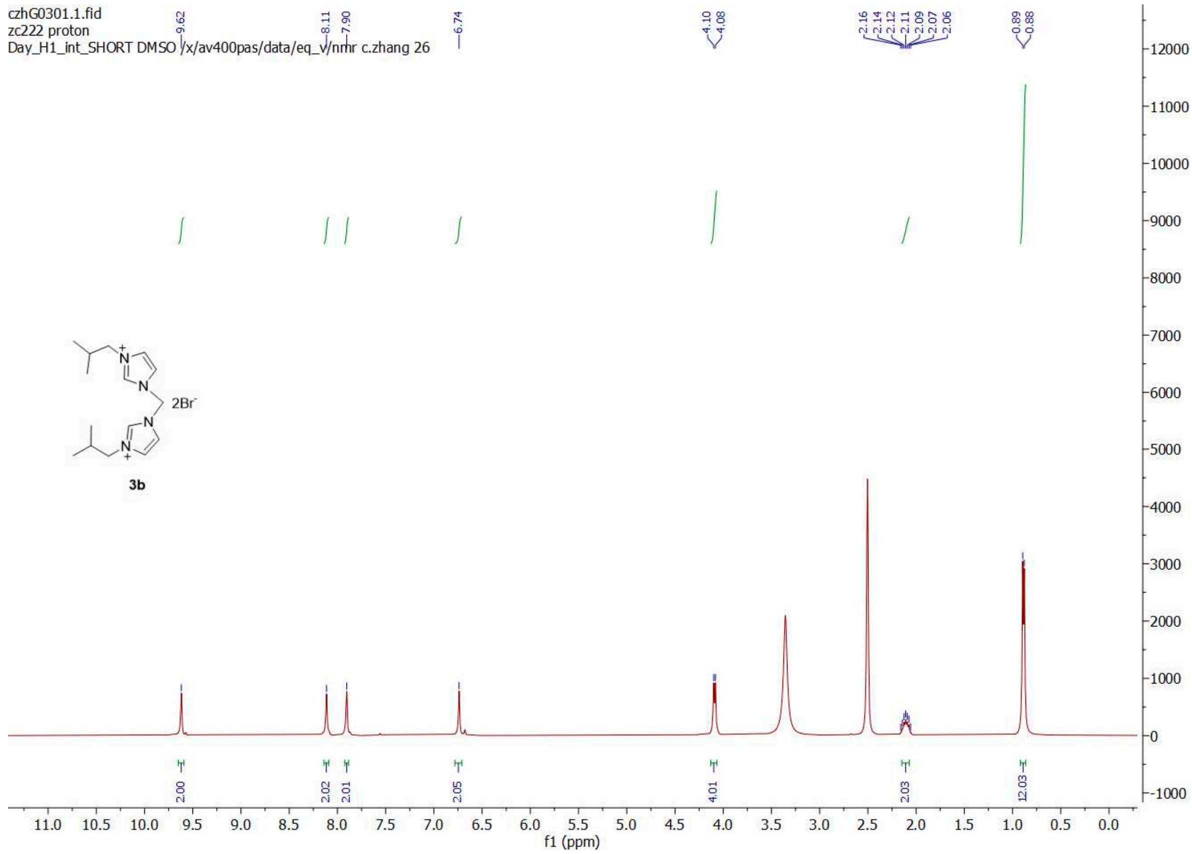
czhG0301.1.fid
zc222 proton
Day_H1_int_SHORT DMSO /x/av400pas/data/eq_v/nmr c.zhang 26
9.62
8.11
7.90
6.74
4.10
4.08
2.16
2.14
2.12
2.11
2.09
2.07
2.06
0.89
0.88
2.00
2.02
2.01
2.05
4.01
2.03
12.03
2Br⁻
3b
f1 (ppm)

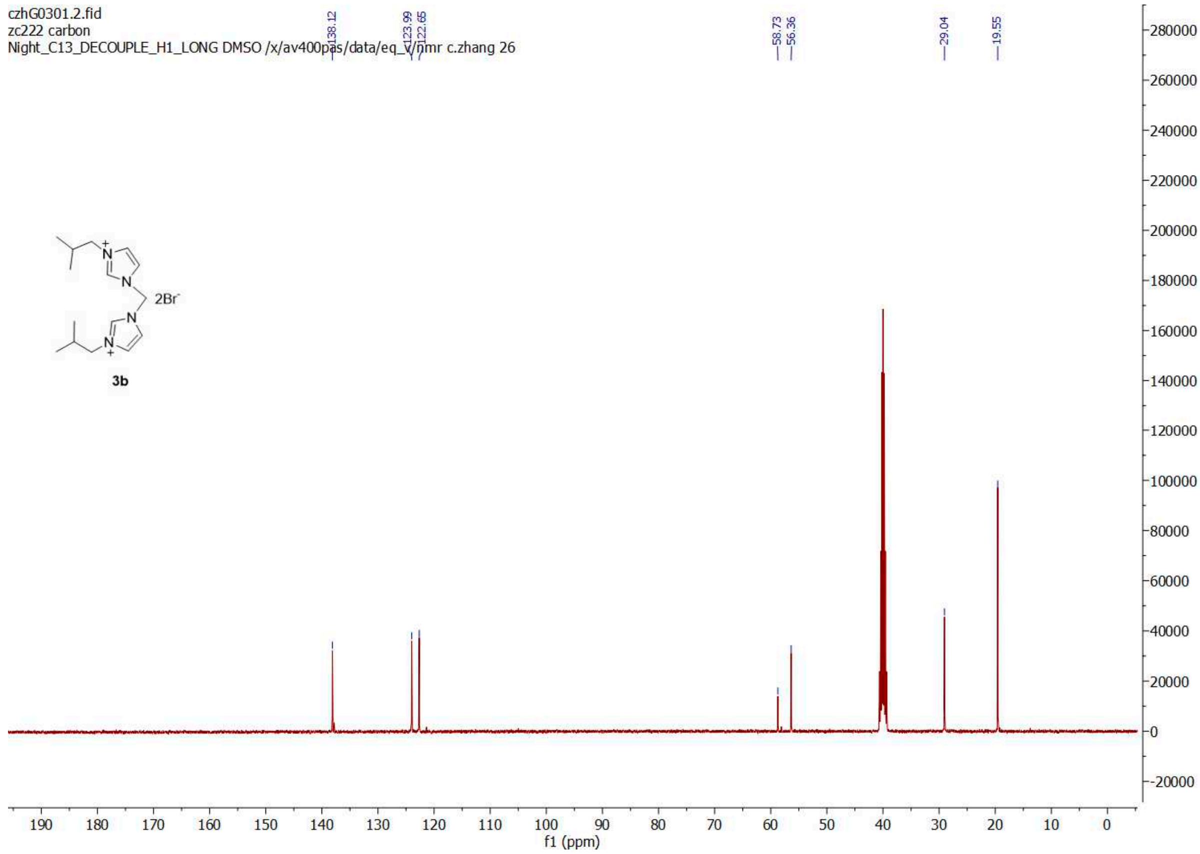
czhG0301.2.fid
zc222 carbon
Night_C13_DECOUPLE_H1_LONG DMSO /x/av400pas/data/eq_v/nmr c.zhang 26
138.12
123.99
122.65
58.73
56.36
29.04
19.55
2Br⁻
3b
f1 (ppm)

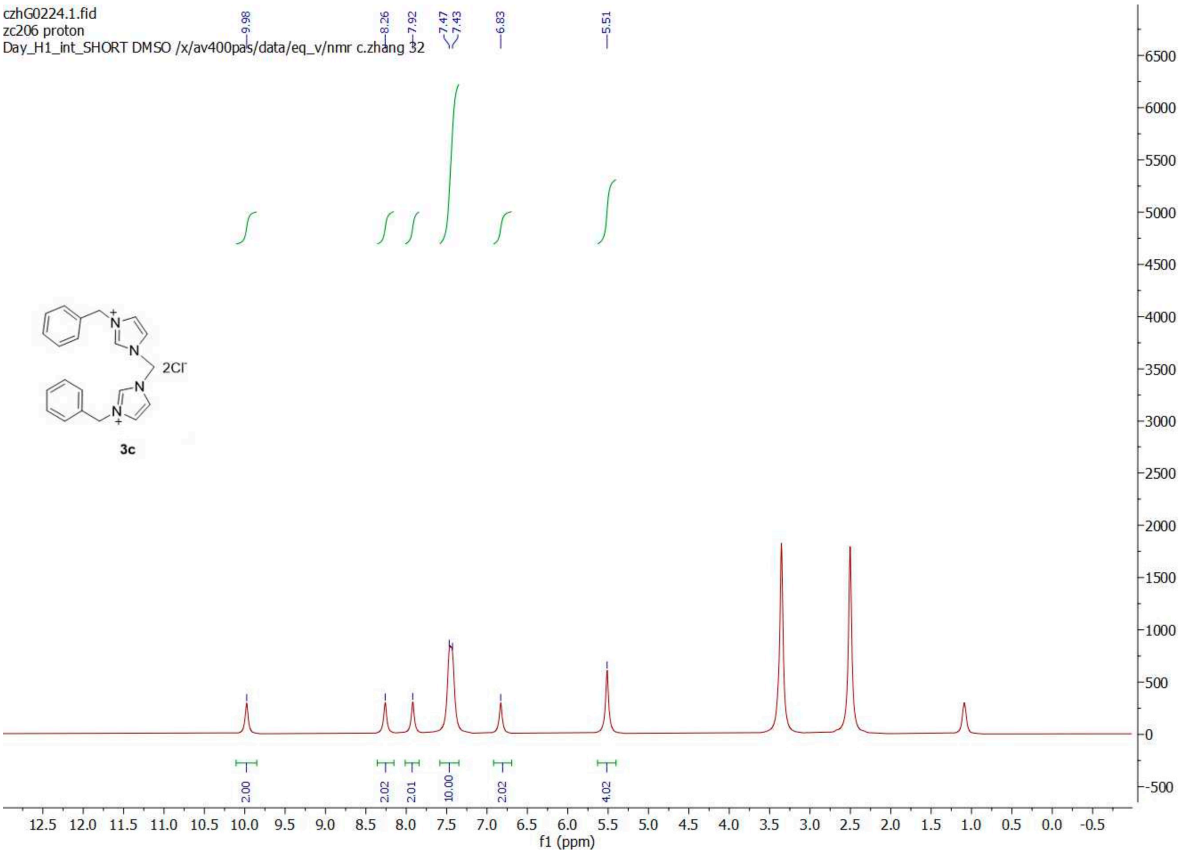
czhG0224.1.fid
zc206 proton
Day_H1_int_SHORT DMSO /x/av400pas/data/eq_v/nmr c.zhang 32
9.98
8.26
7.92
7.47
7.43
6.83
5.51
2Cl⁻
3c
2.00
2.02
2.01
10.00
2.02
4.02
f1 (ppm)

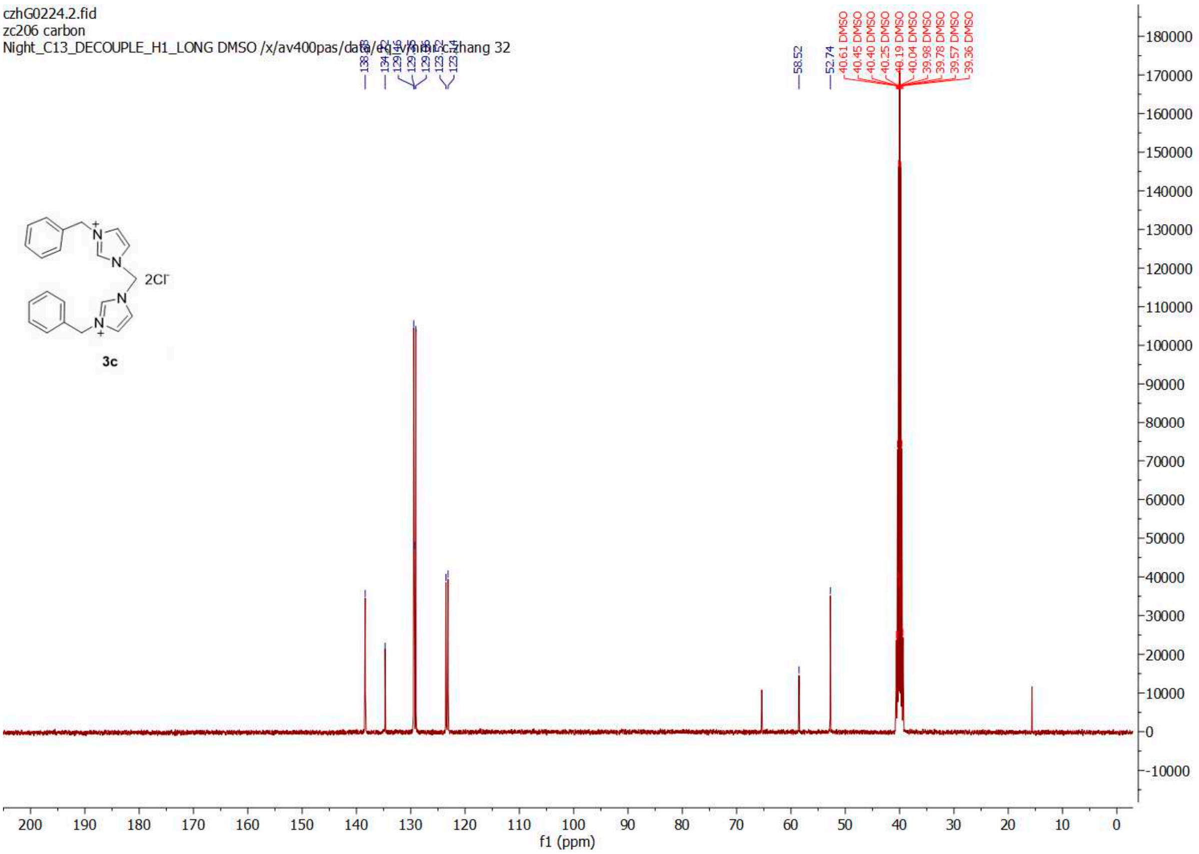
czhG0224.2.fid
zc206 carbon
Night_C13_DECOUPLE_H1_LONG DMSO /x/av400pas/data/eq_v/nmr c.zhang 32
58.52
52.74
40.61 DMSO
40.45 DMSO
40.40 DMSO
40.25 DMSO
40.19 DMSO
40.04 DMSO
39.98 DMSO
39.78 DMSO
39.57 DMSO
39.36 DMSO
2Cl⁻
3c
f1 (ppm)

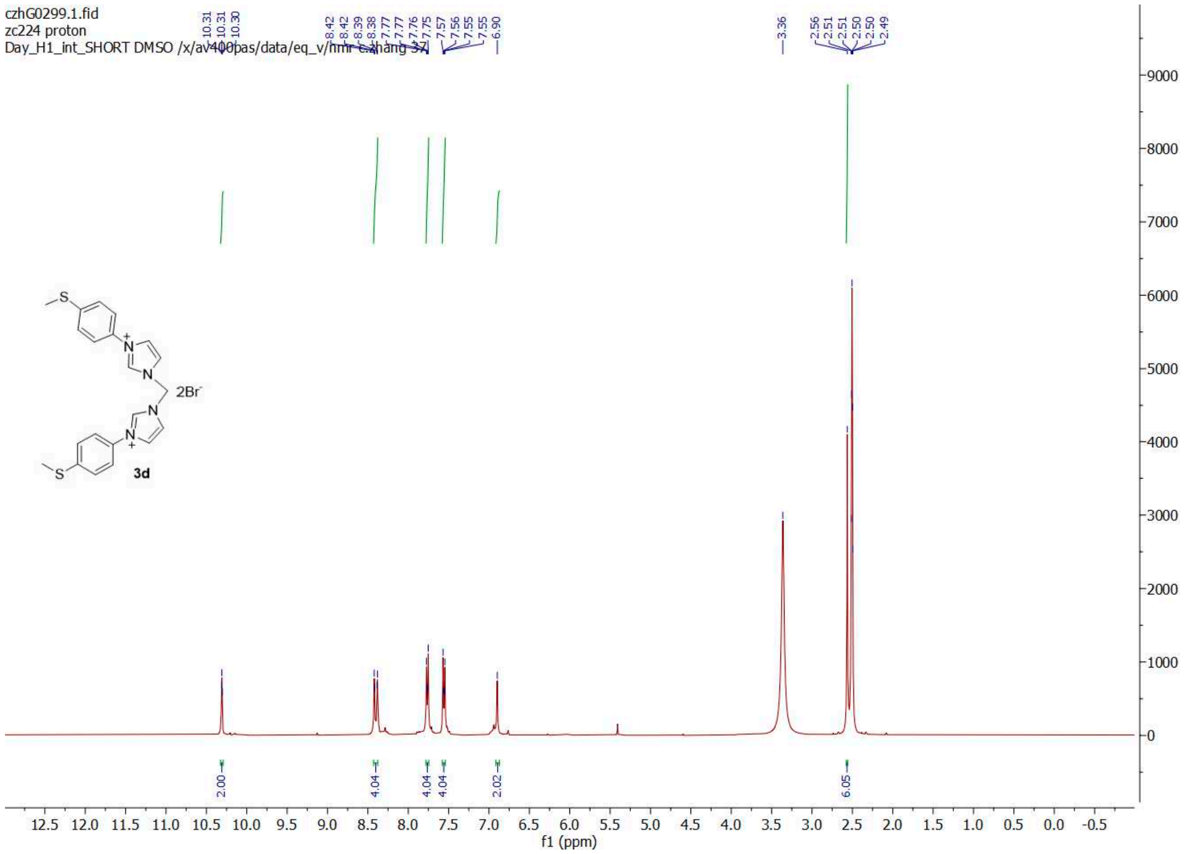
czhG0299.1.fid
zc224 proton
Day_H1_int_SHORT DMSO /x/av400pas/data/eq_v/nmr c.zhang 37
10.31
10.31
10.30
8.42
8.42
8.39
8.38
7.77
7.77
7.76
7.75
7.57
7.56
7.55
7.55
6.90
3.36
2.56
2.51
2.51
2.50
2.50
2.49
2Br-
3d
2.00
4.04
4.04
4.04
2.02
6.05
f1 (ppm)

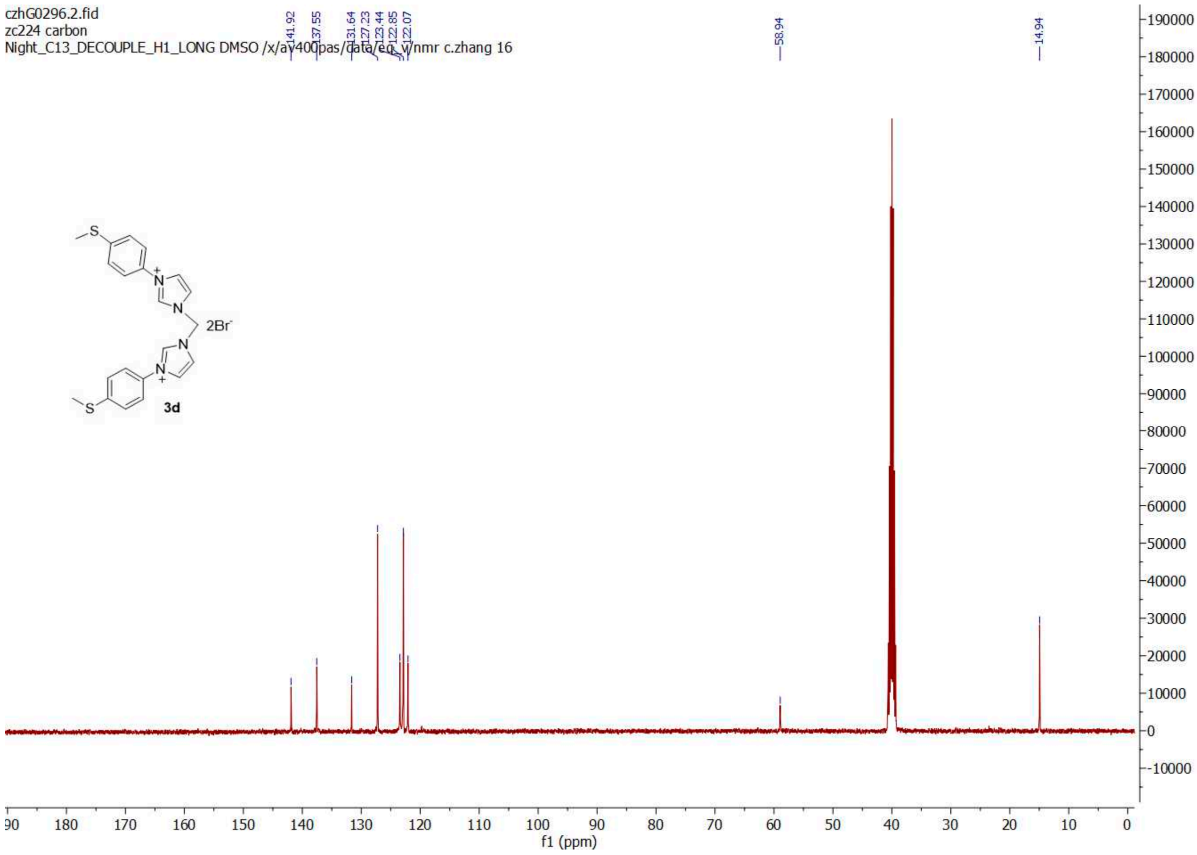
czhG0296.2.fid
zc224 carbon
Night_C13_DECOUPLE_H1_LONG DMSO /x/av400pas/data/eq_v/nmr c.zhang 16
141.92
137.55
131.64
127.23
123.44
122.85
122.07
58.94
14.94
2Br-
3d
f1 (ppm)

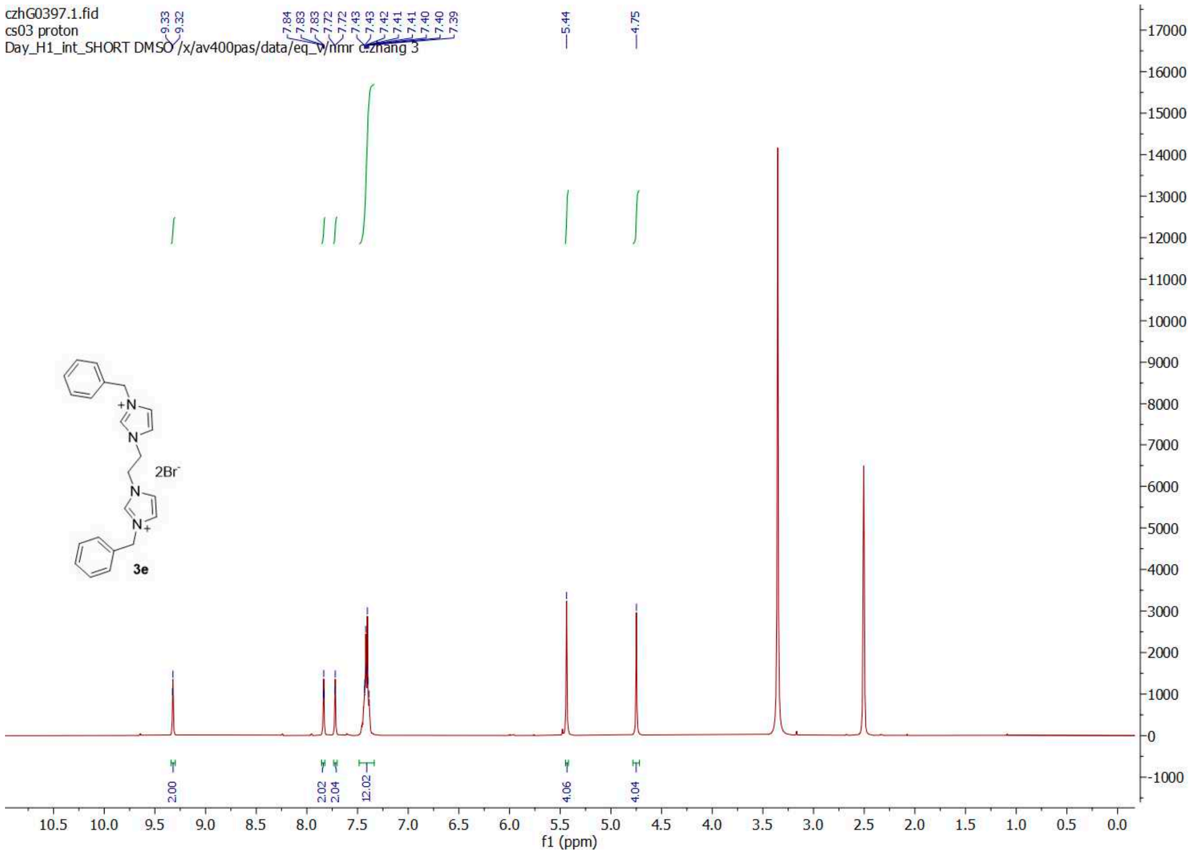
czhG0397.1.fid
cs03 proton
Day_H1_int_SHORT DMSO /x/av400pas/data/eq_v/nmr c.zhang 3
9.33
9.32
7.84
7.83
7.83
7.72
7.72
7.43
7.43
7.42
7.41
7.41
7.40
7.40
7.39
5.44
4.75
2Br-
3e
2.00
2.02
2.04
12.02
4.06
4.04
f1 (ppm)

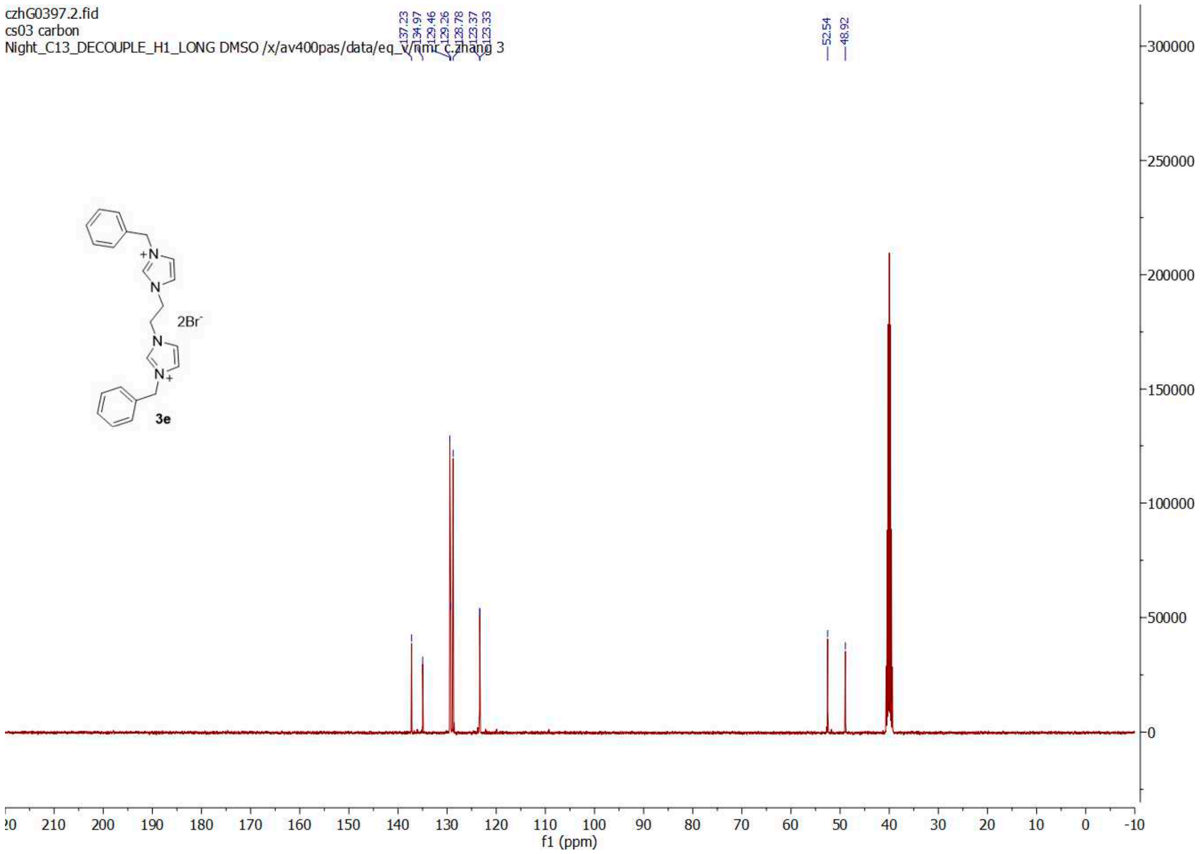
czhG0397.2.fid
cs03 carbon
Night_C13_DECOUPLE_H1_LONG DMSO /x/av400pas/data/eq_v/nmr c.zhang 3
137.23
134.97
129.46
129.26
128.78
123.37
123.33
52.54
48.92
2Br-
3e
f1 (ppm)

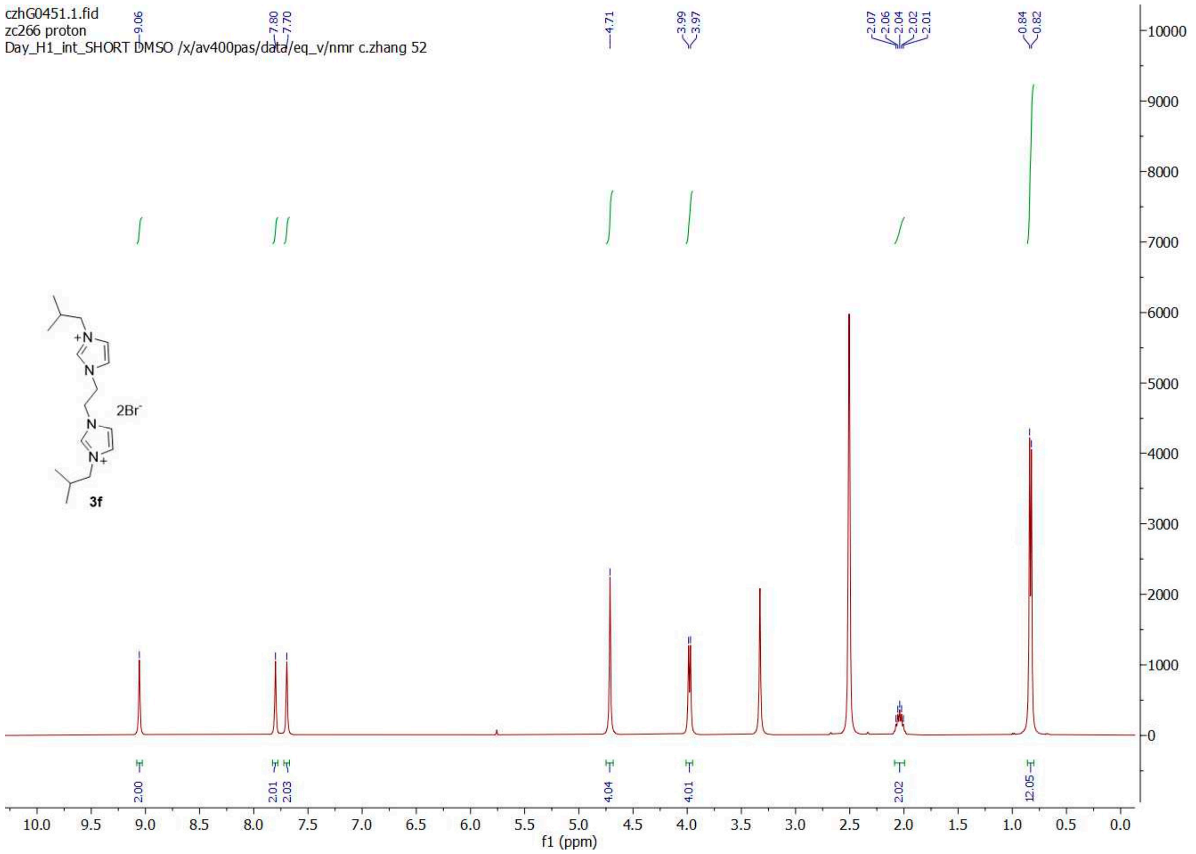
czhG0451.1.fid
zc266 proton
Day_H1_int_SHORT DMSO /x/av400pas/data/eq_v/nmr c.zhang 52
9.06
7.80
7.70
4.71
3.99
3.97
2.07
2.06
2.04
2.02
2.01
0.84
0.82
2Br-
3f
2.00
2.01
2.03
4.04
4.01
2.02
12.05
f1 (ppm)

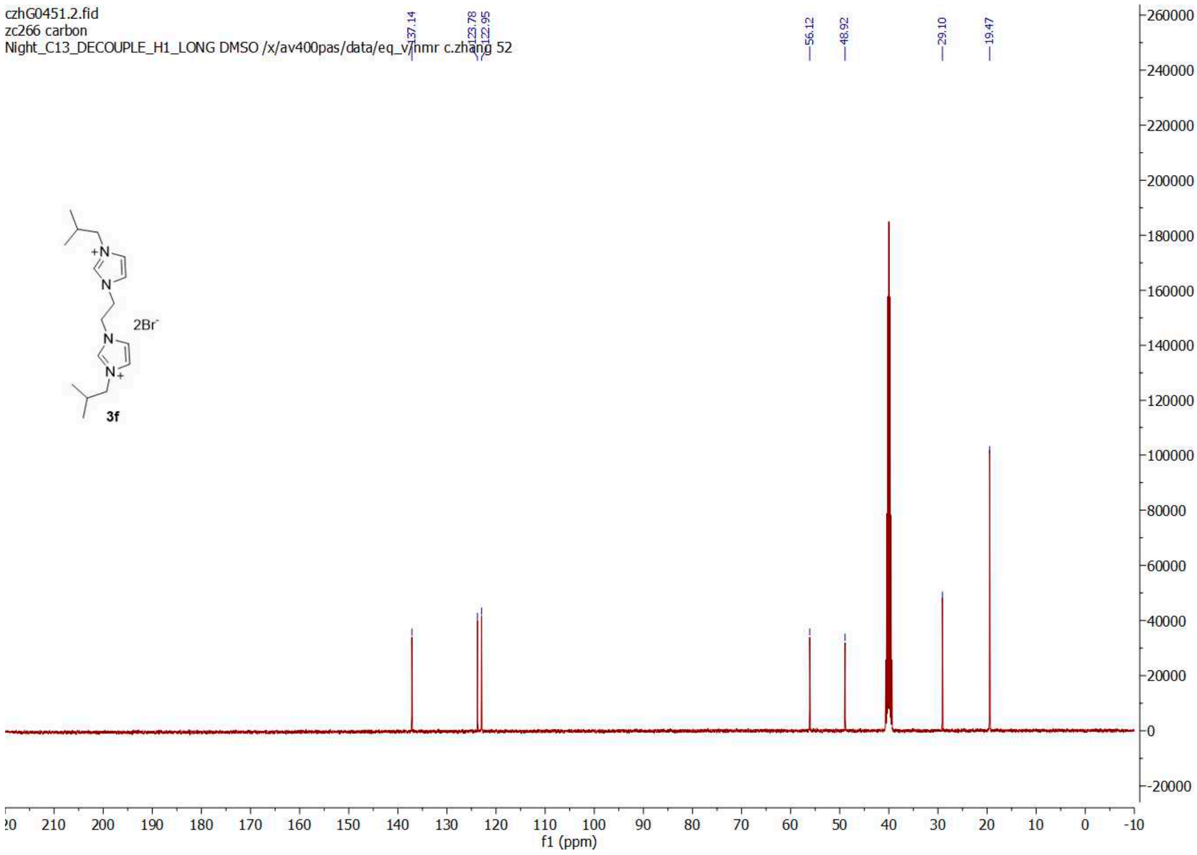
czhG0451.2.fid
zc266 carbon
Night_C13_DECOUPLE_H1_LONG DMSO /x/av400pas/data/eq_v/nmr c.zhang 52
137.14
123.78
122.95
56.12
48.92
29.10
19.47
2Br-
3f
f1 (ppm)

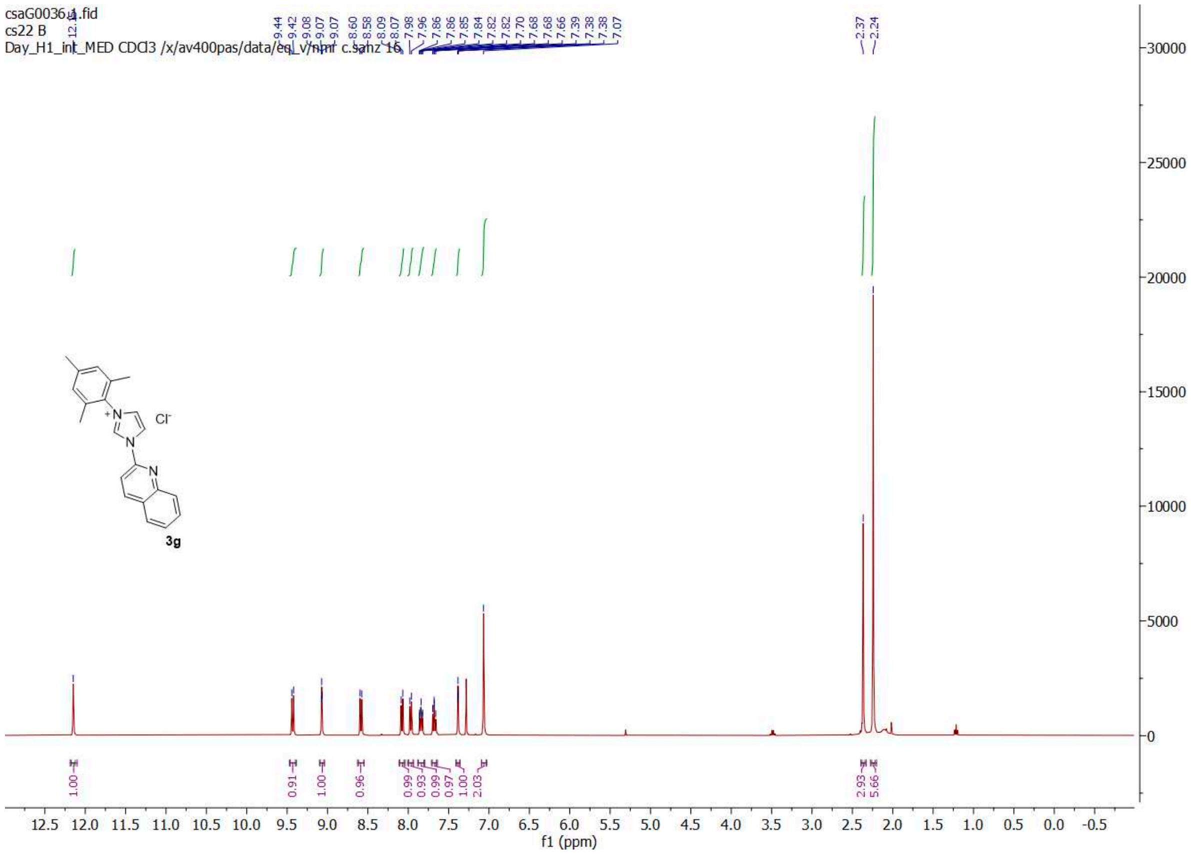
csaG0036.1.fid
cs22 B
Day_H1_int_MED CDCl3 /x/av400pas/data/eq_v/nmr c.sanz 16
9.44
9.42
9.08
9.07
9.07
8.60
8.58
8.09
8.07
7.98
7.96
7.86
7.86
7.85
7.84
7.82
7.82
7.70
7.68
7.68
7.66
7.39
7.38
7.38
7.07
2.37
2.24
Cl⁻
N
3g
1.00
0.91
1.00
0.96
0.99
0.93
0.99
0.97
1.00
2.03
2.93
5.66
f1 (ppm)

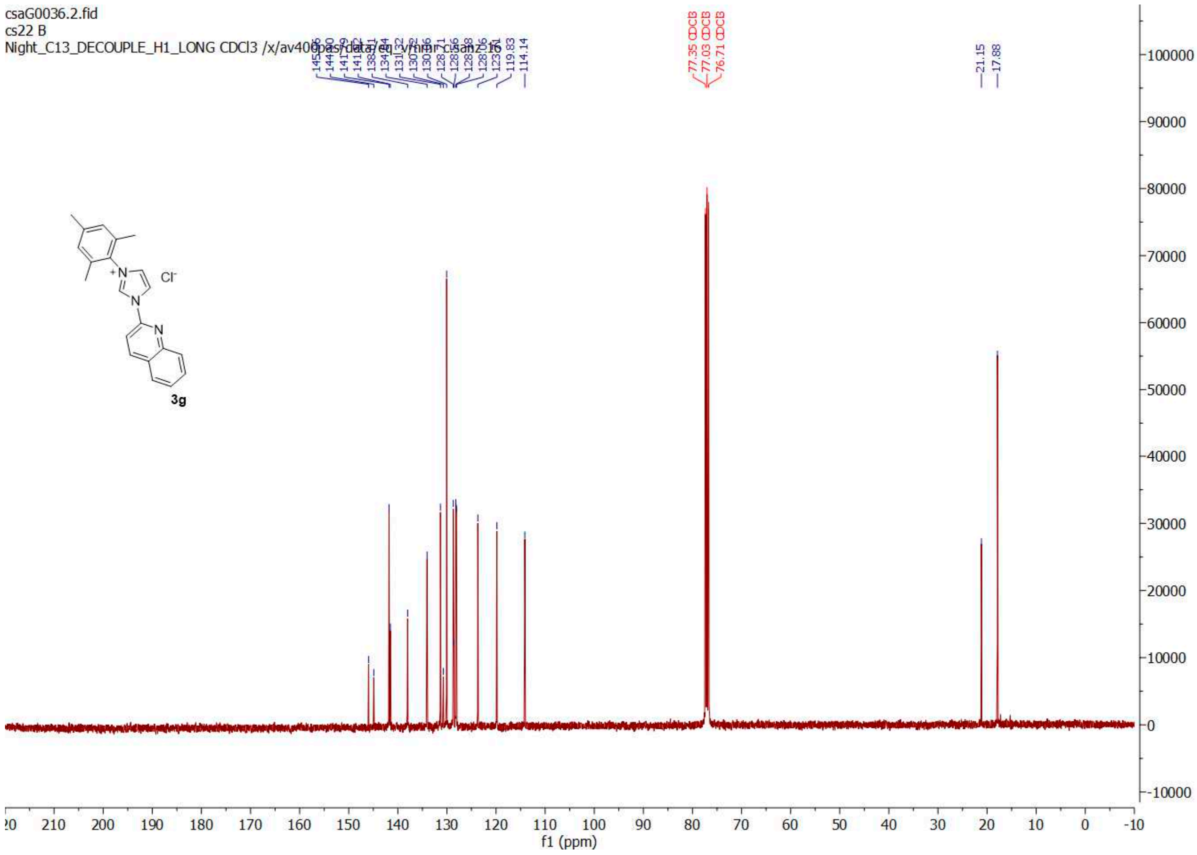
csaG0036.2.fid
cs22 B
Night_C13_DECOUPLE_H1_LONG CDCl3 /x/av400pas/data/eq_v/nmr c.sanz 16
119.83
114.14
77.35 CDCl3
77.03 CDCl3
76.71 CDCl3
21.15
17.88
Cl⁻
N
3g
f1 (ppm)

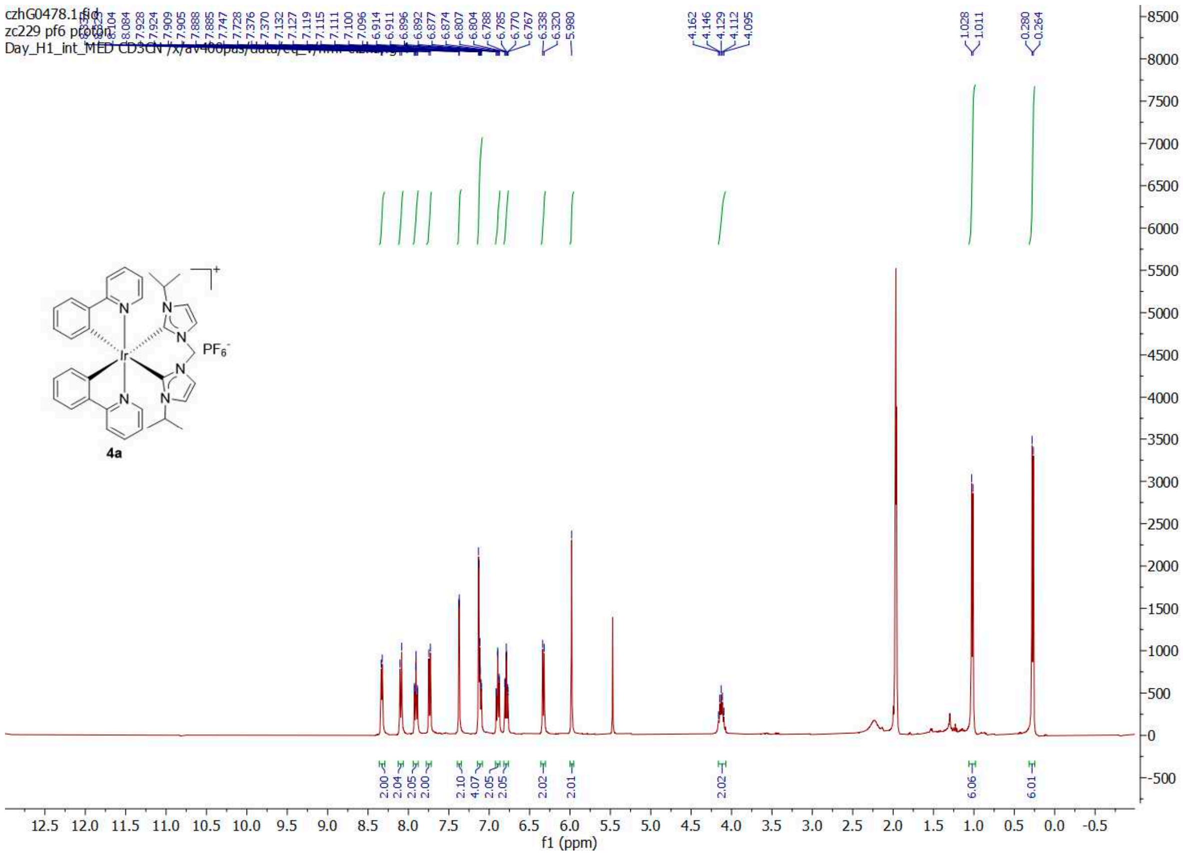
czhG0478.1.fid
zc229 pf6 proton
PF6-
4a
f1 (ppm)

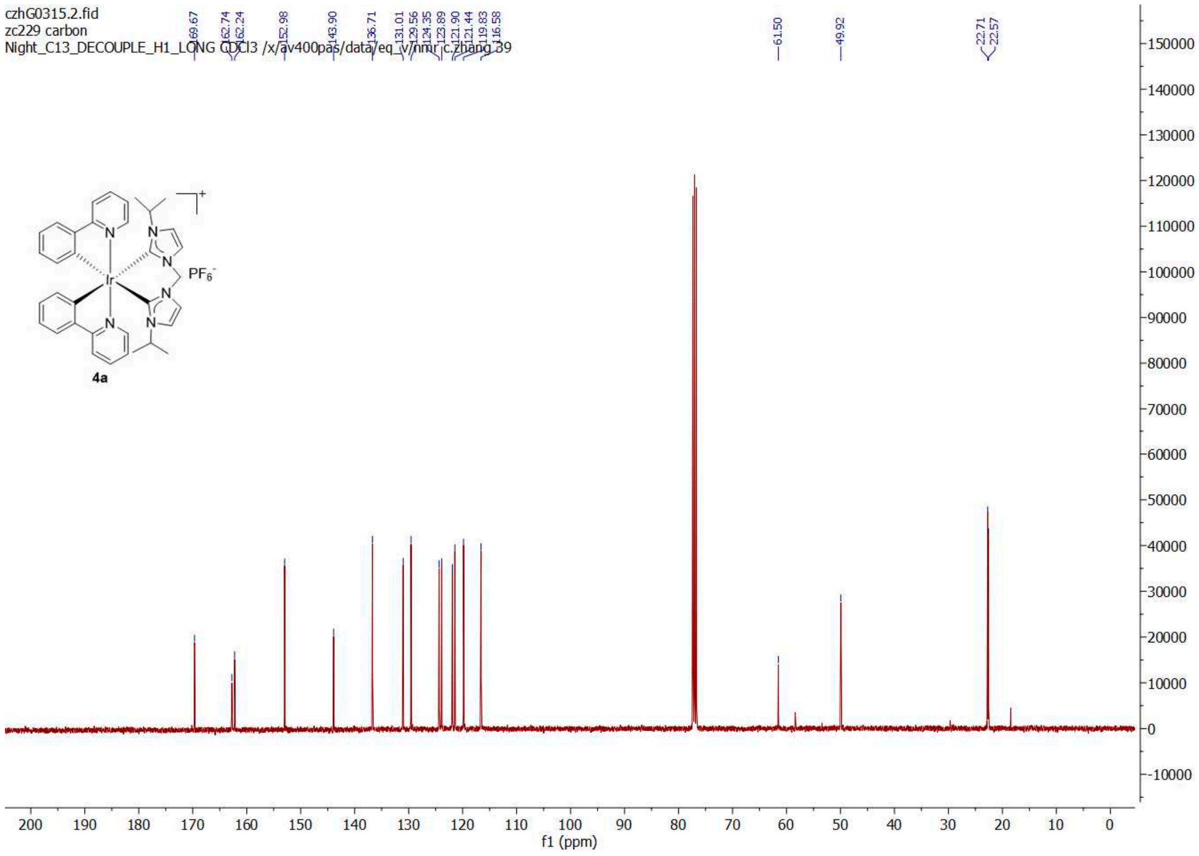
czhG0315.2.fid
zc229 carbon
Night_C13_DECOUPLE_H1_LONG CDCl3 /x/av400pas/data/eq_v/nmr c.zhang 39
PF6-
4a
f1 (ppm)

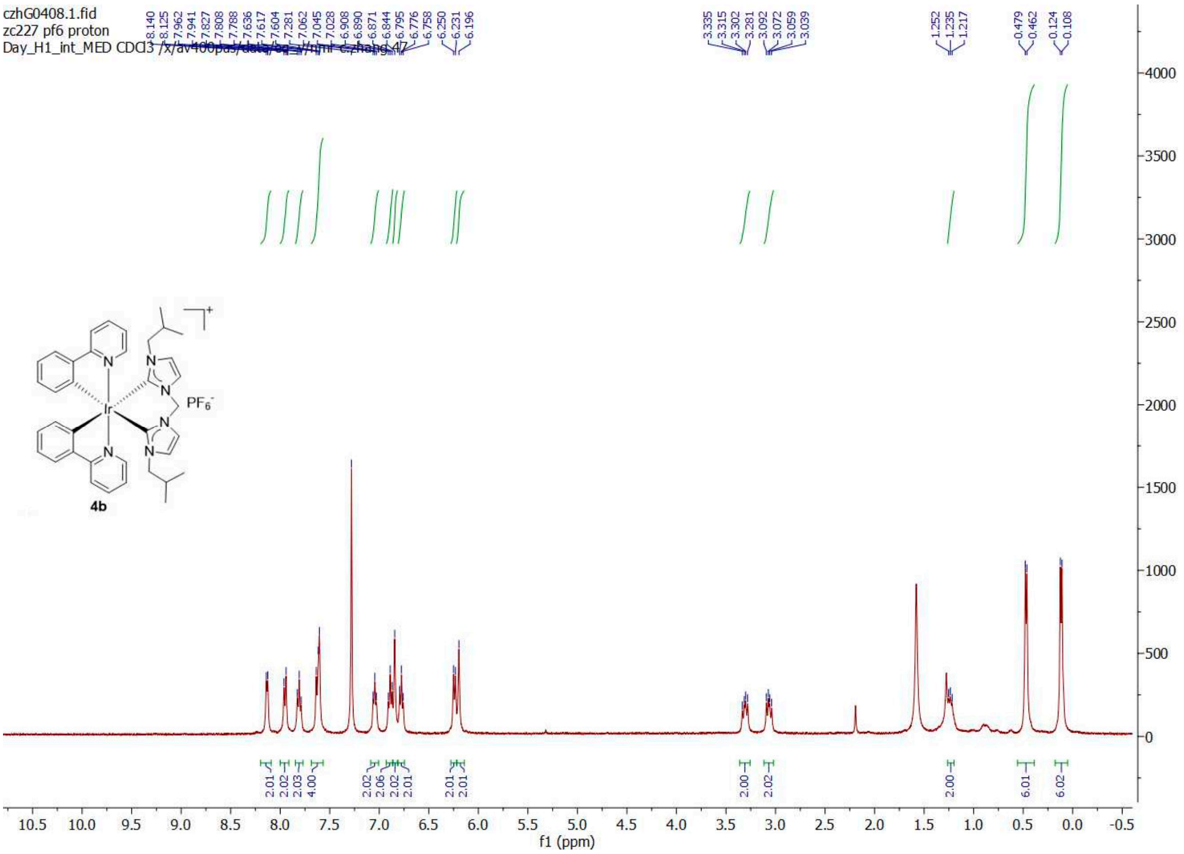
czhG0408.1.fid
zc227 pf6 proton
Day_H1_int_MED CDCl3 /x/av400pas/data/eq_v/nmr c.zhang 47
8.140 8.125 7.962 7.941 7.827 7.808 7.788 7.636 7.617 7.604 7.281 7.062 7.045 7.028 6.908 6.890 6.871 6.844 6.795 6.776 6.758 6.250 6.231 6.196
3.335 3.315 3.302 3.281 3.092 3.072 3.059 3.039
1.252 1.235 1.217
0.479 0.462 0.124 0.108
2.01 2.02 2.03 4.00 2.02 2.06 2.02 2.01 2.01 2.01 2.00 2.02 2.00 6.01 6.02
Ir
PF6-
4b
f1 (ppm)

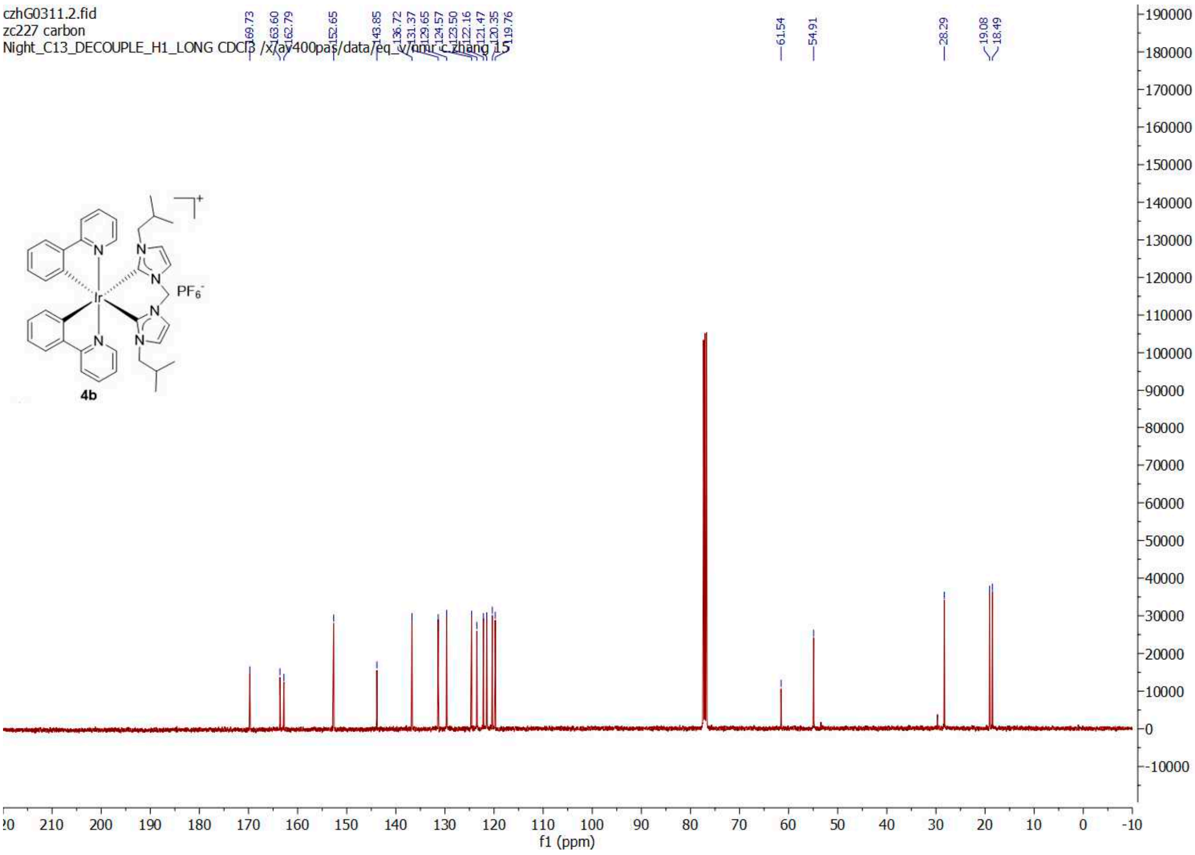
czhG0311.2.fid
zc227 carbon
Night_C13_DECOUPLE_H1_LONG CDCl3 /x/av400pas/data/eq_v/nmr c.zhang 15
169.73 163.60 162.79 152.65 143.85 136.72 131.37 129.65 124.57 123.50 122.16 121.47 120.35 119.76
61.54 54.91 28.29 19.08 18.49
Ir
PF6-
4b
f1 (ppm)

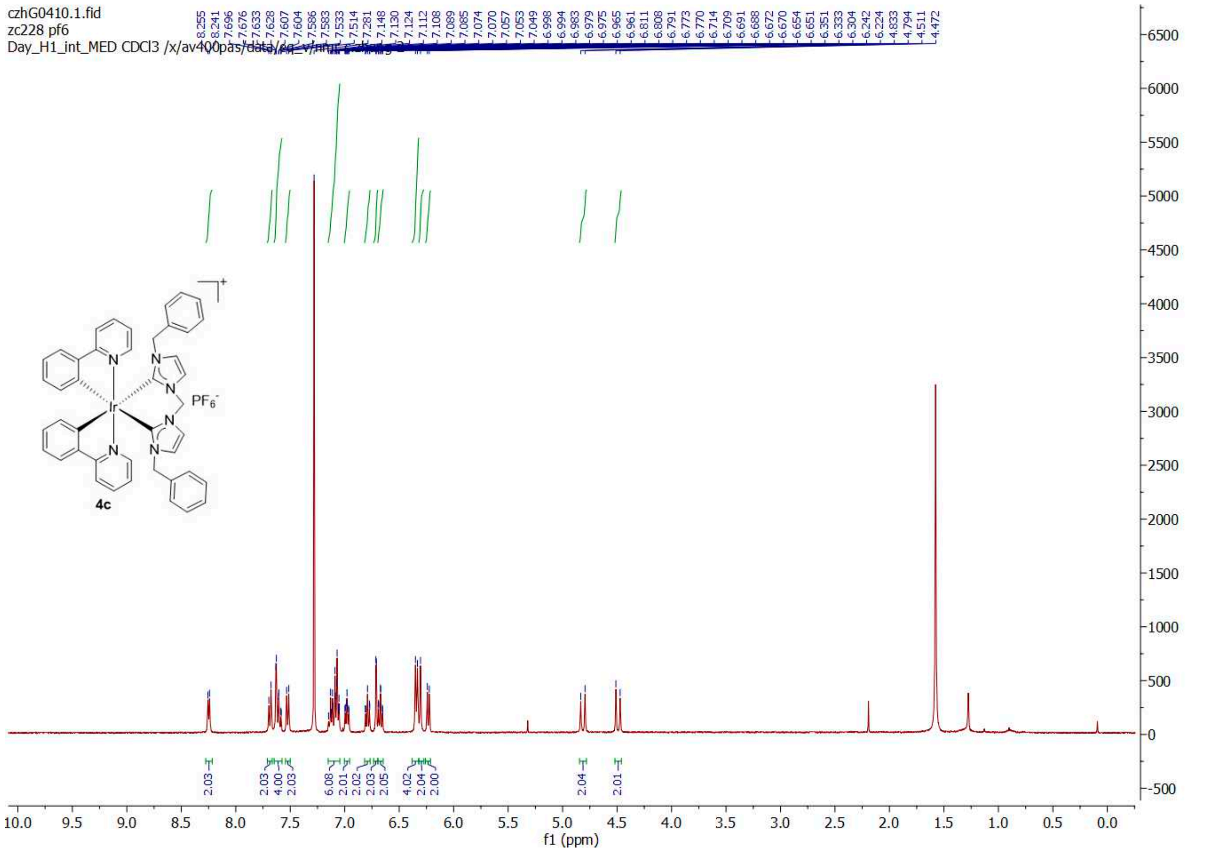
czhG0410.1.fid
zc228 pf6
Day_H1_int_MED CDCl3 /x/av400pas/data/eq_v/nmr
8.255 8.241 7.696 7.676 7.633 7.628 7.607 7.604 7.586 7.583 7.533 7.514 7.281 7.130 7.148 7.124 7.112 7.108 7.089 7.085 7.074 7.070 7.057 7.053 7.049 6.998 6.994 6.983 6.979 6.965 6.961 6.811 6.808 6.791 6.773 6.770 6.714 6.709 6.691 6.688 6.672 6.670 6.654 6.651 6.351 6.333 6.304 6.242 6.224 4.833 4.794 4.511 4.472
PF6-
4c
2.03 2.03 4.00 2.03 6.08 2.01 2.02 2.03 2.05 4.02 2.04 2.00 2.04 2.01
f1 (ppm)

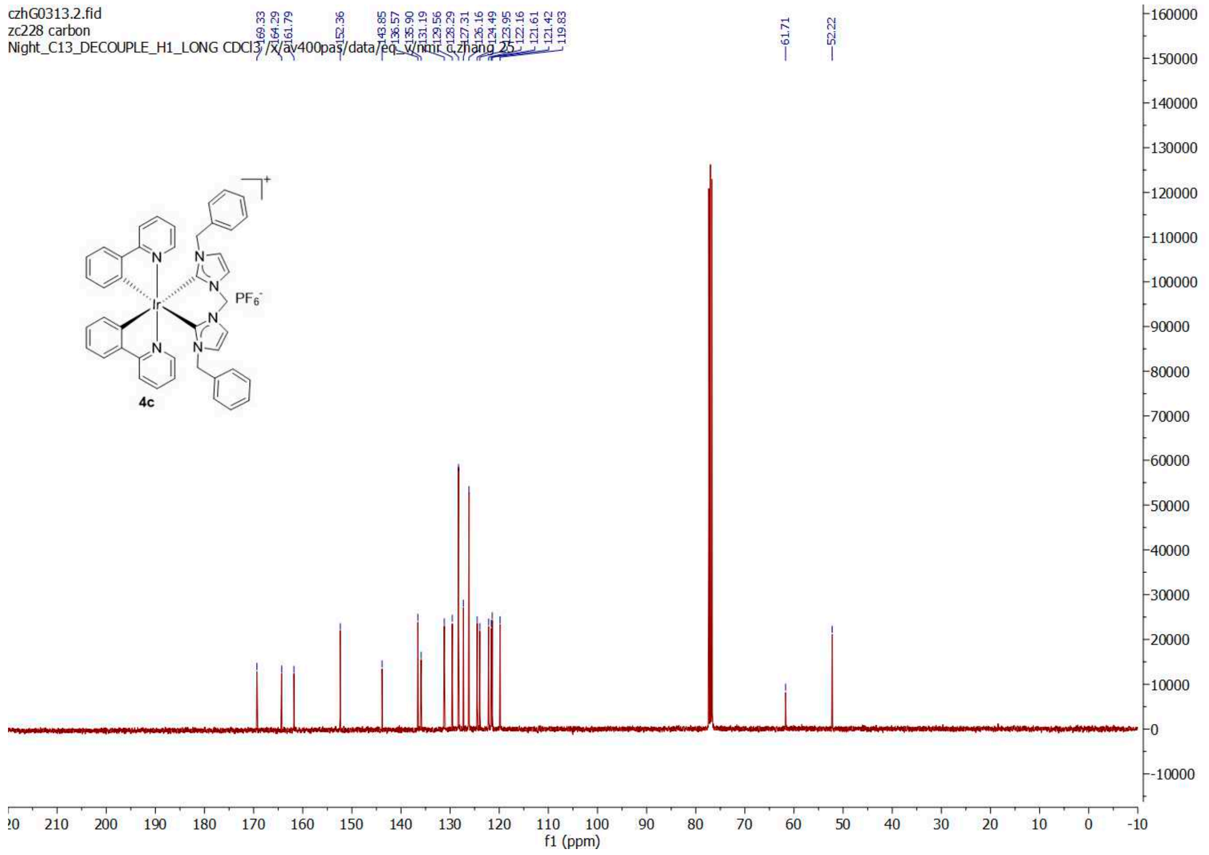
czhG0313.2.fid
zc228 carbon
Night_C13_DECOUPLE_H1_LONG CDCl3 /x/av400pas/data/eq_v/nmr c.zhang 25
169.33 164.29 161.79 152.36 143.85 136.57 135.90 131.19 129.56 128.29 127.31 126.16 124.49 123.95 122.16 121.61 121.42 119.83 61.71 52.22
PF6-
4c
f1 (ppm)

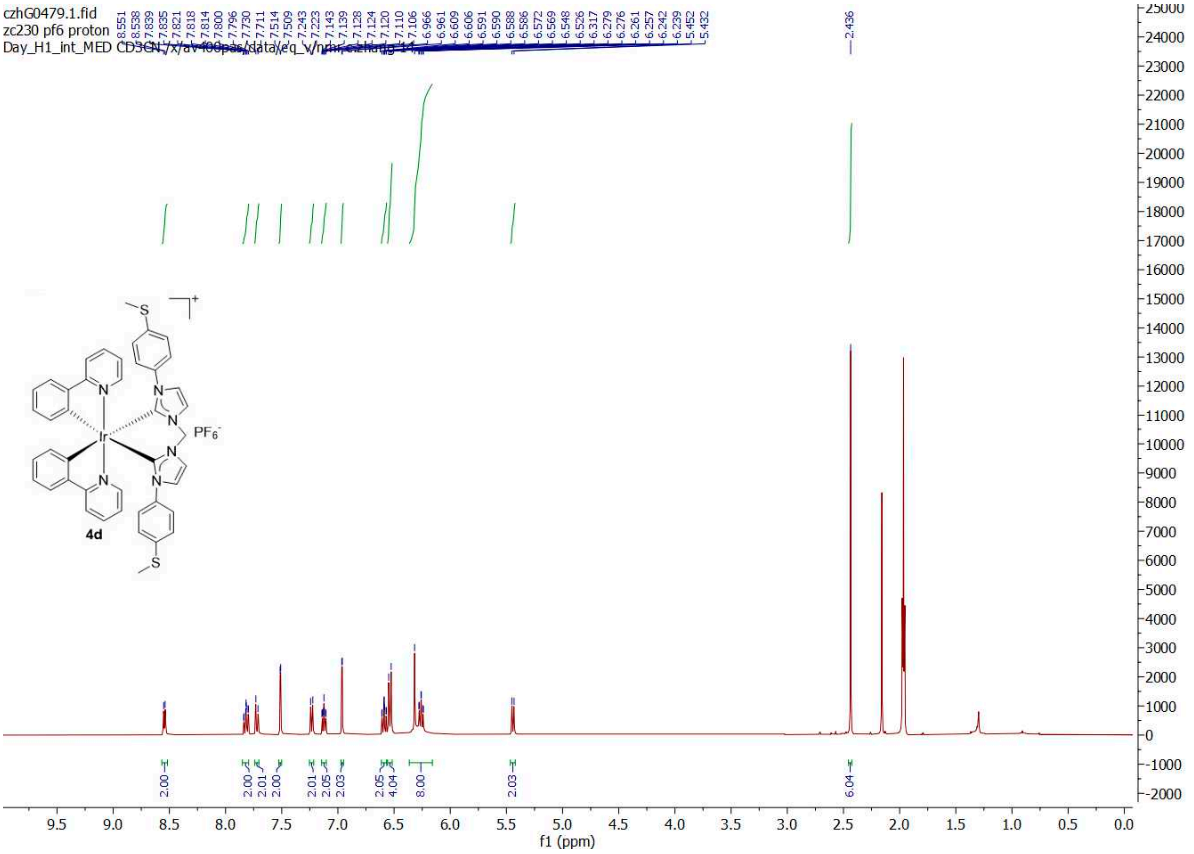

czhG0479.1.fid
zc230 pf6 proton
Day_H1_int_MED CD3CN /x/av400pas/data/eq_v/nmr c.zhang 14
8.551 8.538 7.839 7.835 7.821 7.818 7.814 7.800 7.796 7.730 7.711 7.514 7.509 7.243 7.223 7.143 7.139 7.128 7.124 7.120 7.110 7.106 6.966 6.961 6.609 6.606 6.591 6.590 6.588 6.586 6.572 6.569 6.548 6.526 6.317 6.279 6.276 6.261 6.257 6.242 6.239 5.452 5.432
2.436
2.00 2.00 2.01 2.00 2.01 2.05 2.03 2.05 4.04 8.00 2.03 6.04
PF6
4d
f1 (ppm)

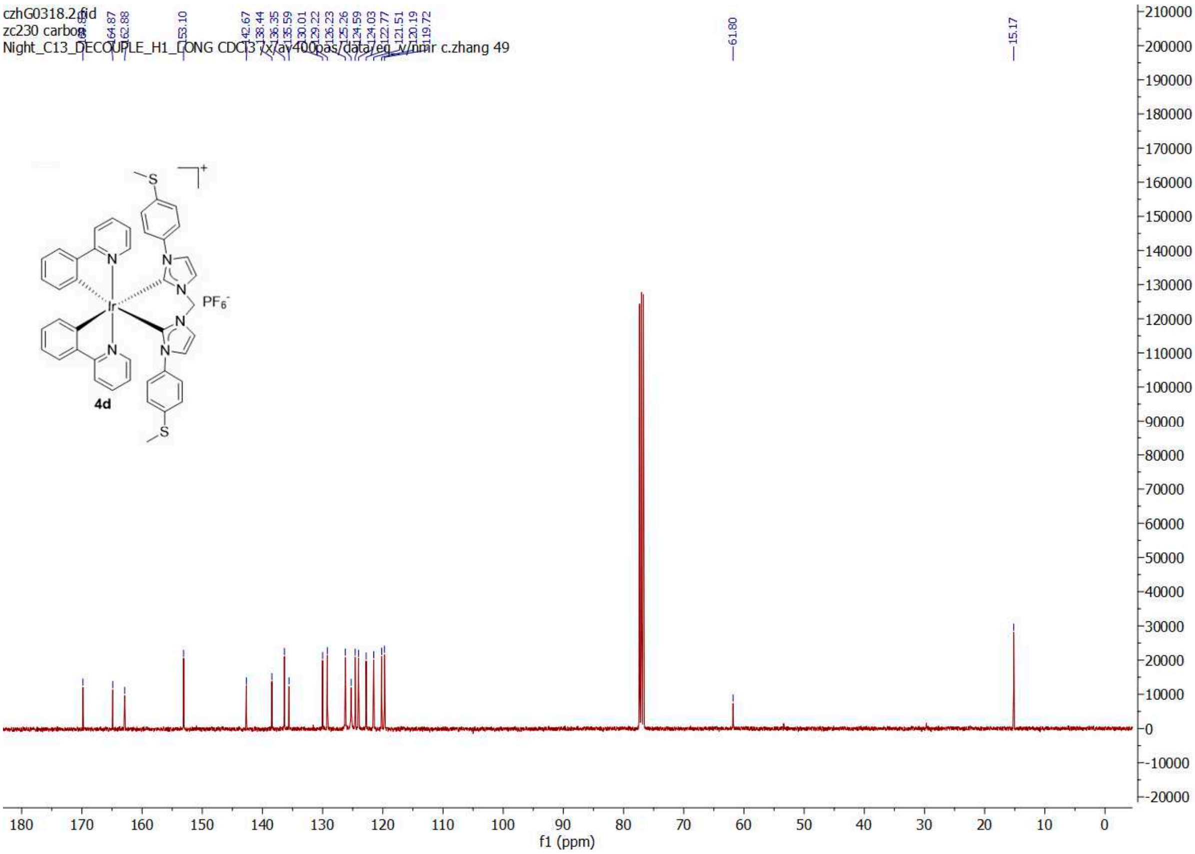

czhG0318.2.fid
zc230 carbon
Night_C13_DECOUPLE_H1_LONG CDCl3 /x/av400pas/data/eq_v/nmr c.zhang 49
164.87 162.88 153.10 42.67 38.44 36.35 35.59 30.01 29.22 26.23 25.26 24.59 24.03 22.77 21.51 20.19 19.72
61.80
15.17
PF6
4d
f1 (ppm)

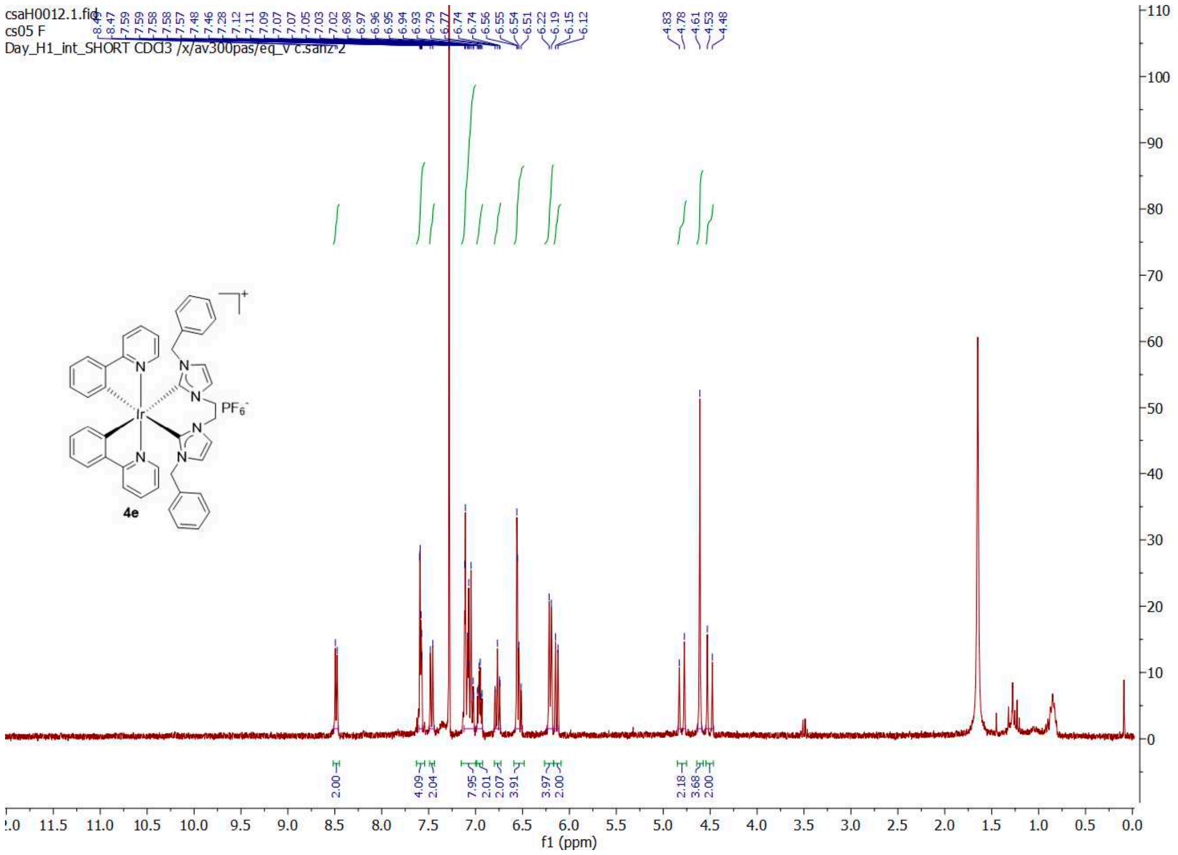
csaH0012.1.fid
cs05 F
Day_H1_int_SHORT CDCl3 /x/av300pas/eq_v c.sanz 2
[illegible] 8.47 7.59 7.59 7.58 7.57 7.48 7.46 7.28 7.12 7.11 7.09 7.07 7.07 7.05 7.03 7.02 6.98 6.97 6.96 6.95 6.94 6.93 6.79 6.77 6.74 6.74 6.56 6.55 6.54 6.51 6.22 6.19 6.15 6.12
4.83 4.78 4.61 4.53 4.48
PF6-
4e
2.00 4.09 2.04 7.95 2.01 2.07 3.91 3.97 2.00 2.18 3.68 2.00
f1 (ppm)

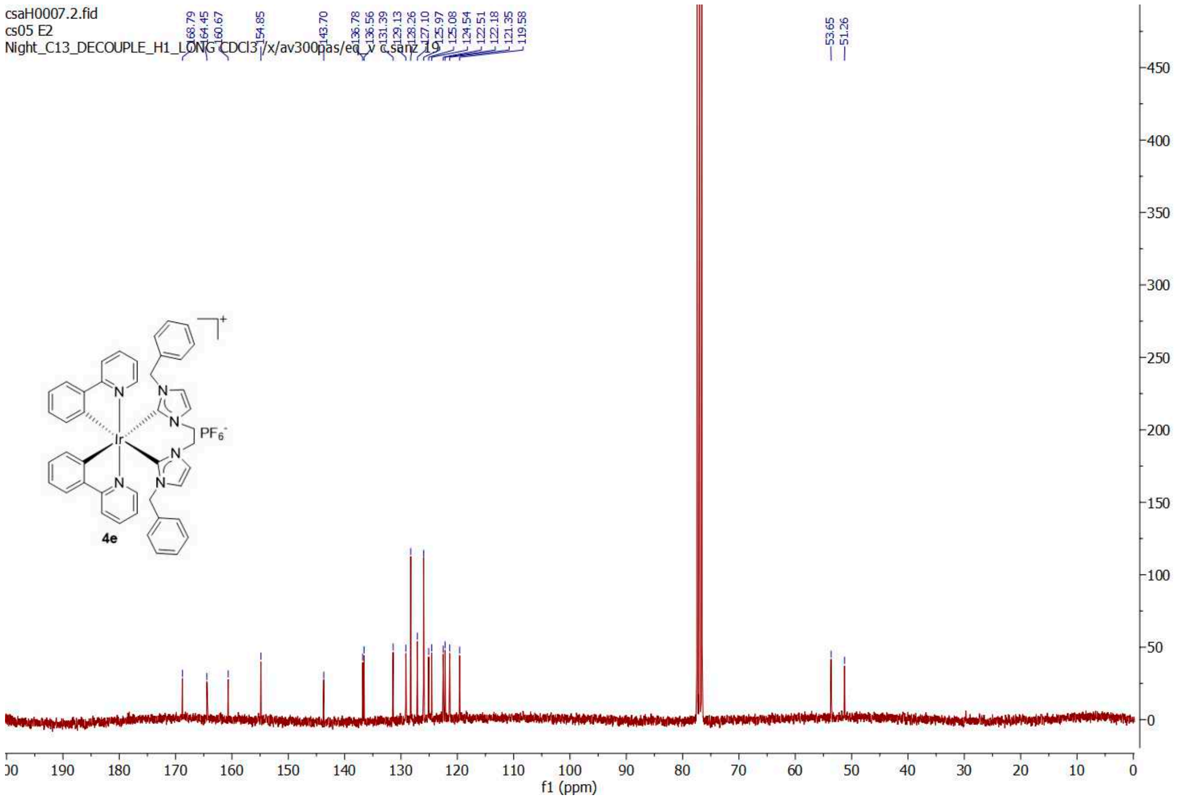
csaH0007.2.fid
cs05 E2
Night_C13_DECOUPLE_H1_LONG CDCl3 /x/av300pas/eq_v c.sanz 19
168.79 164.45 160.67 154.85 143.70 136.78 136.56 131.39 129.13 128.26 127.10 125.97 125.08 124.54 122.51 122.18 121.35 119.58
53.65 51.26
PF6-
4e
f1 (ppm)

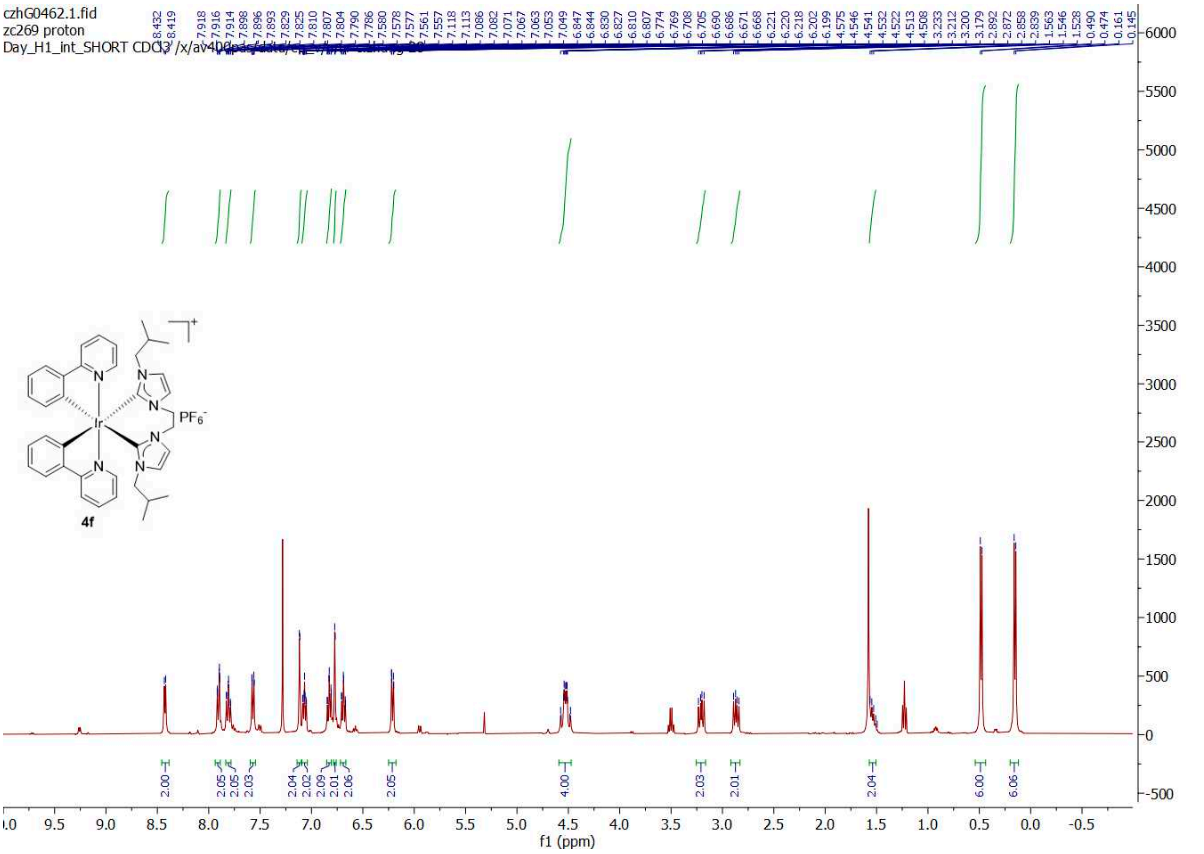
czhG0462.1.fid
zc269 proton
Day_H1_int_SHORT CDCl3 /x/av400pas/data/eq_v/nmr czhang 20
8.432
8.419
7.918
7.916
7.914
7.898
7.896
7.893
7.829
7.825
7.810
7.807
7.804
7.790
7.786
7.580
7.578
7.577
7.561
7.557
7.118
7.113
7.086
7.082
7.071
7.067
7.063
7.053
7.049
6.847
6.844
6.830
6.827
6.810
6.807
6.774
6.769
6.708
6.705
6.690
6.686
6.671
6.668
6.221
6.220
6.218
6.202
6.199
4.575
4.546
4.541
4.532
4.522
4.513
4.508
3.233
3.212
3.200
3.179
2.892
2.872
2.858
2.839
1.563
1.546
1.528
0.490
0.474
0.161
0.145
PF6-
Ir
N
4f
2.00
2.05
2.05
2.03
2.04
2.02
2.09
2.01
2.06
2.05
4.00
2.03
2.01
2.04
6.00
6.06
f1 (ppm)

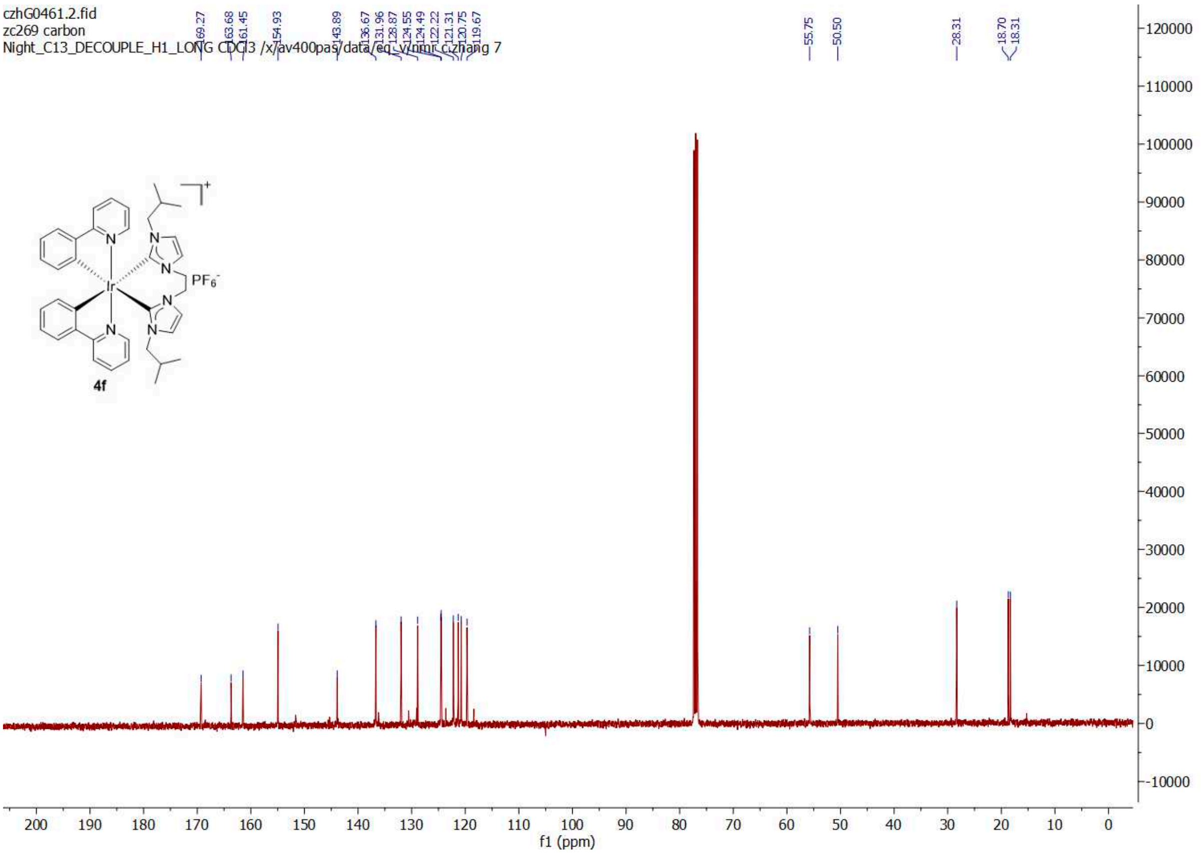
czhG0461.2.fid
zc269 carbon
Night_C13_DECOUPLE_H1_LONG CDCl3 /x/av400pas/data/eq_v/nmr czhang 7
169.27
163.68
161.45
154.93
143.89
136.67
131.96
128.87
124.55
124.49
122.22
121.31
120.75
119.67
55.75
50.50
28.31
18.70
18.31
PF6-
Ir
N
4f
f1 (ppm)

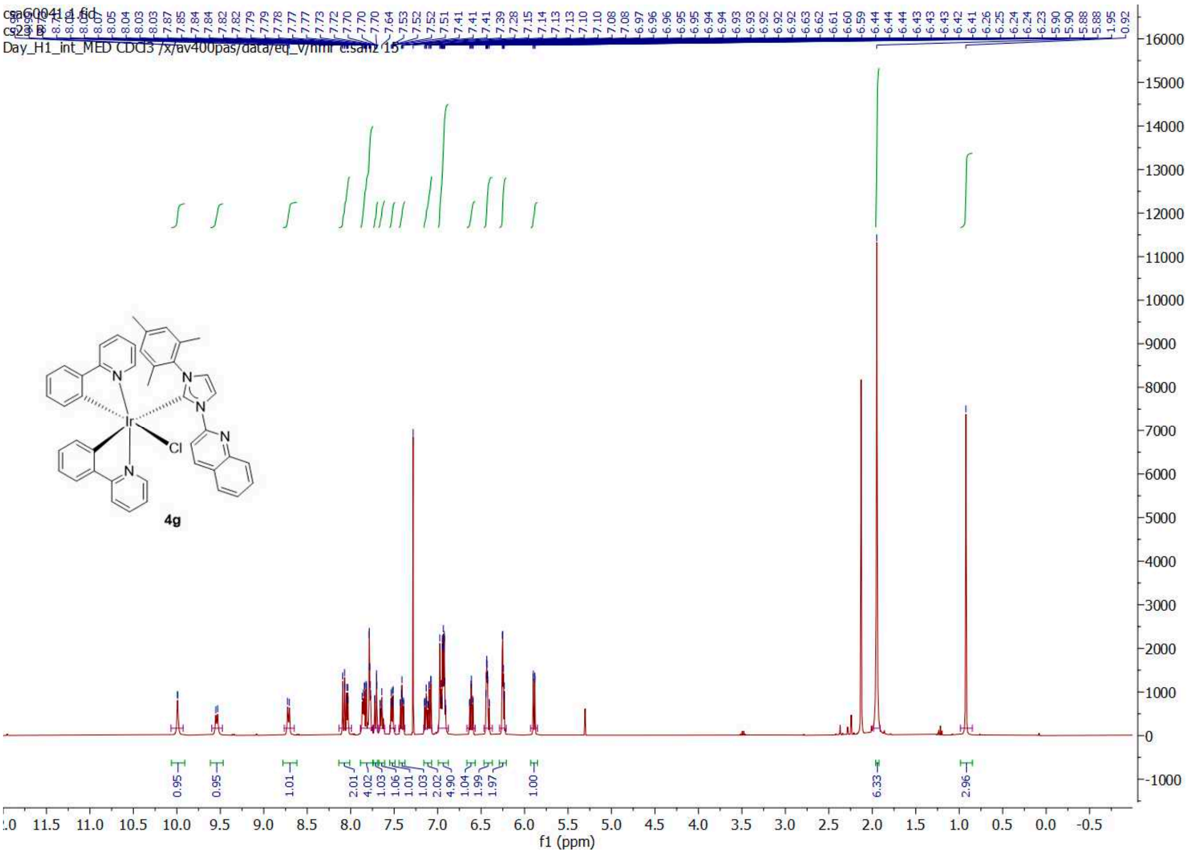
csaG0041.1.fid
cs23 B
Day_H1_int_MED CDCl3 /x/av400pas/data/eq_v/nmr c.sanz 15
4g
f1 (ppm)

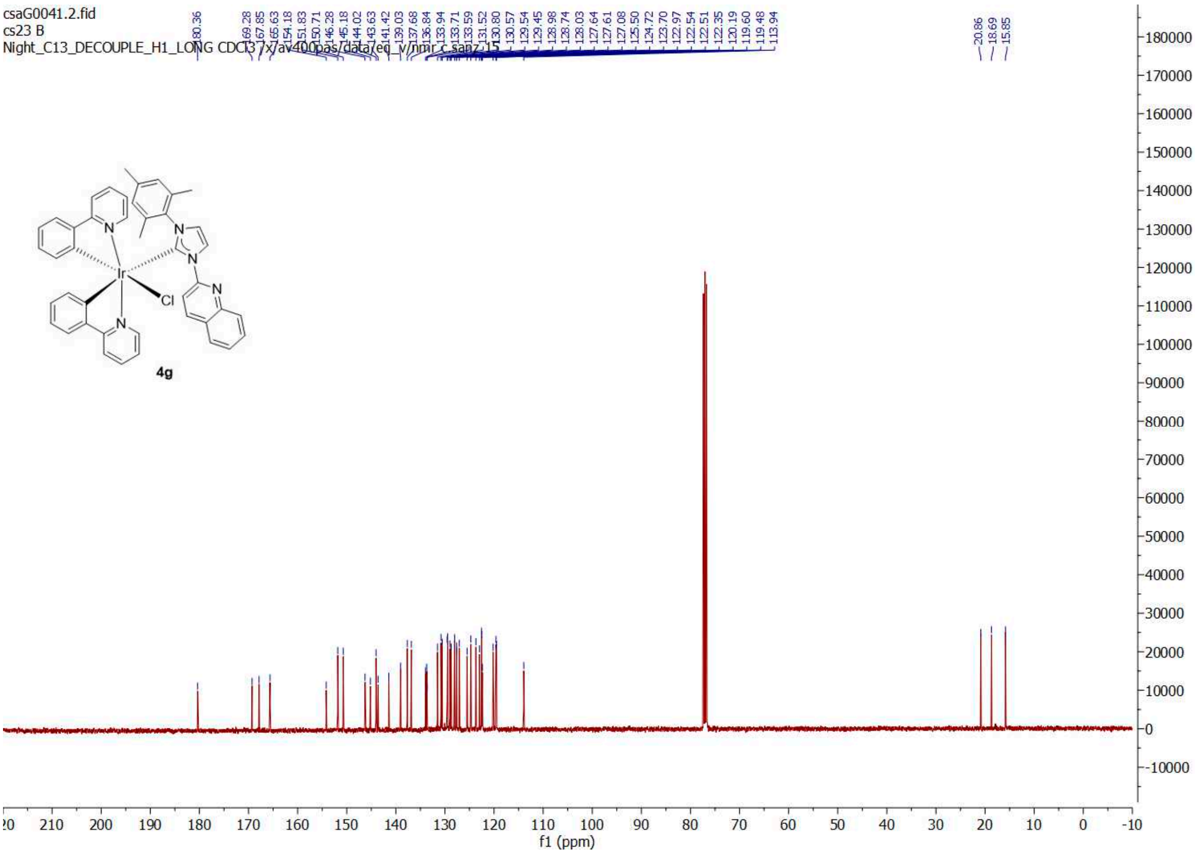
csaG0041.2.fid
cs23 B
Night_C13_DECOUPLE_H1_LONG CDCl3 /x/av400pas/data/eq_v/nmr c.sanz 15
4g
f1 (ppm)

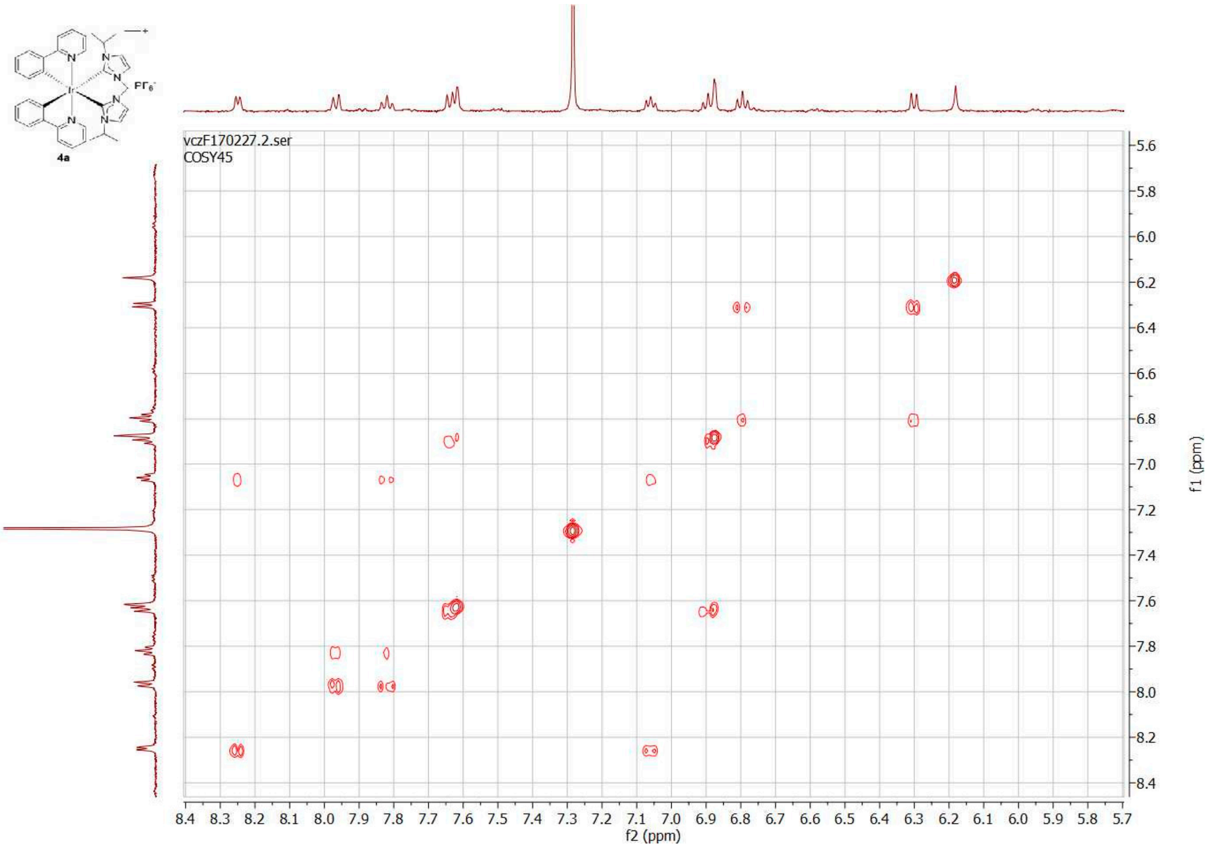
4a
vczF170227.2.ser
COSY45
f2 (ppm)
f1 (ppm)

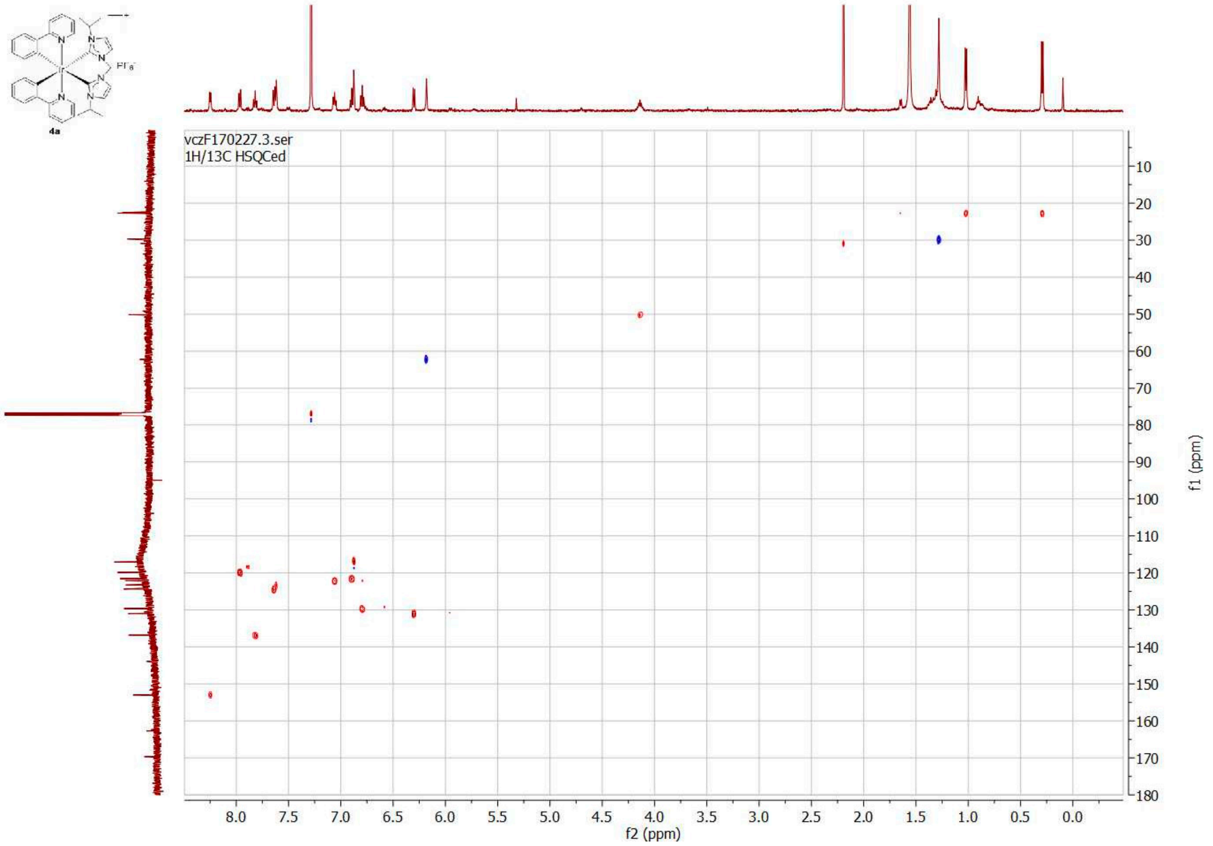
4a
vczF170227.3.ser
1H/13C HSQCed
f2 (ppm)
f1 (ppm)

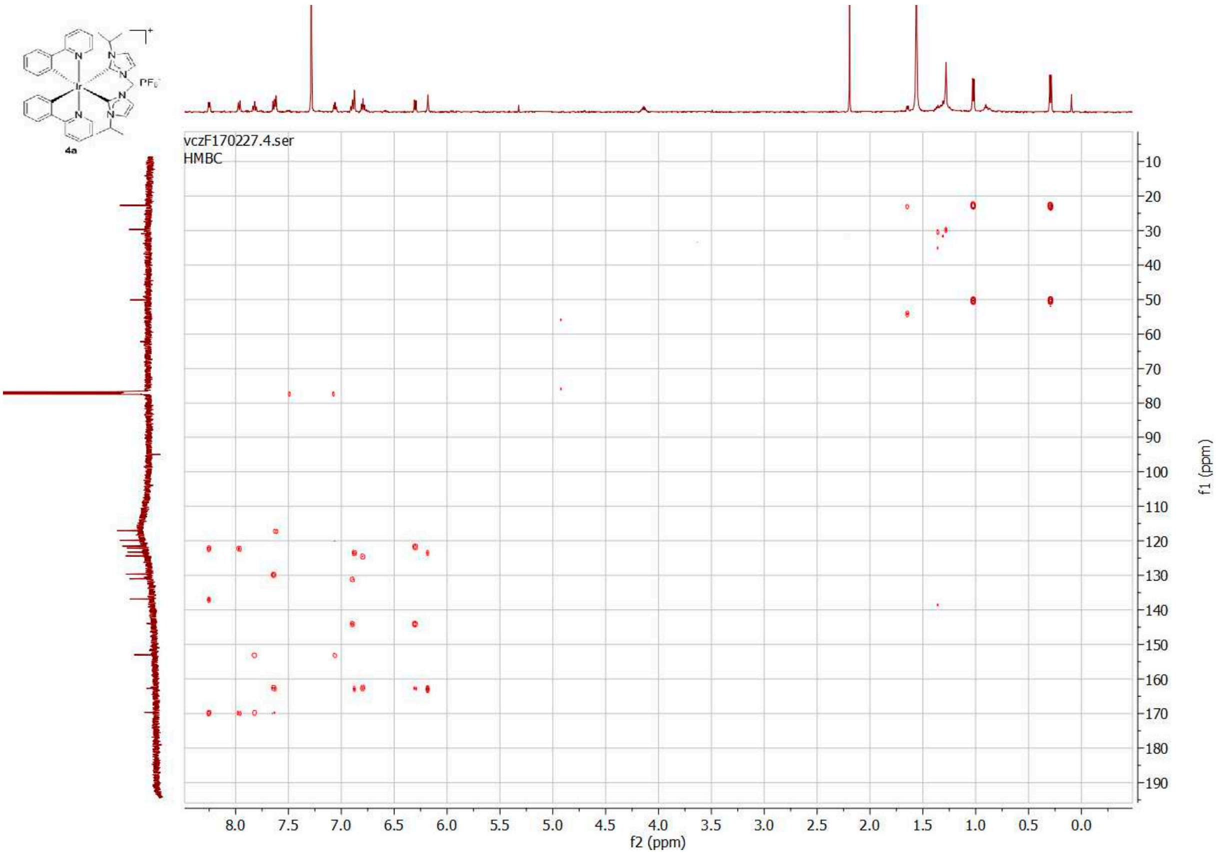

4a
PF6
vczF170227.4.ser
HMBC
f1 (ppm)
f2 (ppm)
